# Supplementary material for: Collateral hypersensitivity between ZY19489 and piperaquine neutralizes PfCRT-mediated drug efflux and Plasmodium falciparum resistance
Source: Nat Commun. 2026 Apr 1;17:5441. doi: 10.1038/s41467-026-70914-1 (PMC13280021; doi:10.1038/s41467-026-70914-1)
Supplement: Supplementary file 1 — Supplementary Information [file 41467_2026_70914_MOESM1_ESM.pdf]

Supplementary Figures and Tables

Collateral hypersensitivity between ZY19489 and piperaquine neutralizes PfCRT-mediated drug efflux and *Plasmodium falciparum* resistance

John Okombo, Tolla Ndiaye, Tarrick Qahash, Igor M. R. Moura, Eva Gil-Iturbe, Laura M. Hagenah, Jessica L. Bridgford, Vinicius Bonatto, Kurt E. Ward, Tomas Yeo, Sunil K. Narwal, Lily V. Orta, Isla Anderson, Satish K. Dhingra, Charisse Flerida A. Pasaje, Heekuk Park, Jonathan Kim, Rafael V. C. Guido, Maria Belén Jiménez-Diaz, Iñigo Angulo-Barturen, Jacquin C. Niles, Filippo Mancia, Anne-Catrin Uhlemann, Sachel Mok, Matthias Quick, Elizabeth A. Winzeler, Didier Leroy, Manuel Llinás, Vandana Thathy, and David A. Fidock

TABLE OF CONTENTS

File name **page**

Supplementary Fig. 1: Conditional knockdown (cKD) of PfV1-D...4

Supplementary Fig. 2: Schematic detailing timelines and experimental approach used to select a ZY19489-resistant mutant *P. falciparum* line..5

Supplementary Fig. 3: Schematic of *pfcrt* and *pfapc10* gene editing. 6

Supplementary Fig. 4: Chemical structures of antimalarial compounds included in this study...................7

Supplementary Fig. 5: Molecular dynamics simulations showing intramolecular configurations for 7G8 (parental) and 7G8+N246H (mutant) PfCRT isoforms that differentially impact transporter interactions with ZY19489 8

Supplementary Fig. 6: Molecular dynamics simulations with 7G8 and 7G8+N246H PfCRT....................9

Supplementary Fig. 7: Parasitemia profiles in NSG mice engrafted with human erythrocytes (TADhuMouse®) treated with ZY19489-based combinations..18

Supplementary Fig. 8: Heatmap of log_2 fold changes of 3D7 and Dd2 treated with ZY19489 atovaquone...19

Supplementary Fig. 9: Inhibition of recombinant *P. falciparum* cGMP-dependent protein kinase (PfPKG). 20

Supplementary Fig. 10: PfHDP expression levels do not impact ZY19489 activity against *P. falciparum* asexual blood stages..21

Supplementary Fig. 11: Evidence that falcipain 2A or 2B do not modulate parasite susceptibility to ZY19489...22

Supplementary Fig. 12: Gating strategy for flow cytometry-based quantification of *P. falciparum* asexual blood stage parasite proliferation in drug susceptibility and growth rate assays..................................23

Supplementary Table 1: Haplotypes of known drug resistance genes of the edited and control *P. falciparum* lines used in this study...24

Supplementary Table 2: *In vitro* activity of ZY19489 against PfV1-D cKD lines...............................25

Supplementary Table 3: *In vitro* 72-hour susceptibility of drug-selected bulk parasites and clones tested against ZY19489...26

Supplementary Table 4: Genes within the amplified ~80kb locus of chromosome 5 in clone C8...............27

Supplementary Table 5: *In vitro* 72-hour susceptibility of edited parasites clones against ZY19489..28

Supplementary Table 6: *In vitro* 72-hour potency of APC10 and ubiquitin ligase inhibitors and chloroquine as a control inhibitor against $Dd2^{Dd2}$ and $Dd2^{Dd2crt+apc10\ D233N}$ parasites.................................29

Supplementary Table 7: 72-hour *in vitro* susceptibility of FCB and $FCB\text{-}KD^{mdr1}$ parasites against ZY19489, mefloquine (MFQ) and lumefantrine (LMF) 30

Supplementary Table 8: List of 34 genes in the chromosome 7 QTL peak harboring non-synonymous mutations between RF7 and NF54..31

Supplementary Table 9: Chemical Pairwise Linear Potential (ChemPLP) docking scores for ZY19489 and

solvent-accessible surface area (SASA) values..**35**

Supplementary Table 10: *In vitro* 72-hour susceptibility of Dd2^{Dd2crt}, Dd2$^{Dd2crt\ N246H}$, Dd2^{GB4crt} and Dd2^{GB4crtN246H} against 7 clinically important antimalarials……………………………………………**36**

Supplementary Table 11: 72-hour *in vitro* activity of ZY19489 against *pfcrt* and *pfmdr1* mutants harboring contemporary drug-resistant alleles……………………………………………………….…..**37**

Supplementary Table 12: 72-hour *in vitro* activity of ZY19489 and M5717 against M5717-resistant *Pf*eEF2 mutant parasites recovered from humanized NSG mice……………………..................................**38**

Supplementary Table 13: Stage-specific activity against 3D7 parasites of ZY19489 and control antimalarial compounds, chloroquine, MMV390048 and dihydroartemisinin after 12-hour incubation intervals and after 72 hours.………………...**39**

Supplementary Table 14: Averaged $\log_2$ fold change of the baseline metabolite levels in synchronized trophozoite stage 3D7 and Dd2 parasites treated for 2.5 hours with 10× atovaquone or 10× ZY19489 IC_{50} compared to untreated controls. ………...**40**

Supplementary Table 15: Characteristic features of the short hemoglobin (Hb)-derived peptides identified from synchronized trophozoite stage 3D7 and Dd2 parasites treated for 2.5 hours with 10× ZY19489 IC_{50} (100 nM)………………………………………………………………………………………………**43**

Supplementary Table 16: Hemoglobin, 'free' heme and hemozoin levels in synchronized NF54 parasites treated for 30 hours with various concentrations of ZY19489, chloroquine and pyrimethamine…………**44**

Supplementary Table 17: Names and sequences of oligonucleotides used in the editing of *pfcrt* N246H and *pfapc10* D233N mutations……………………………………………………………….………**45**

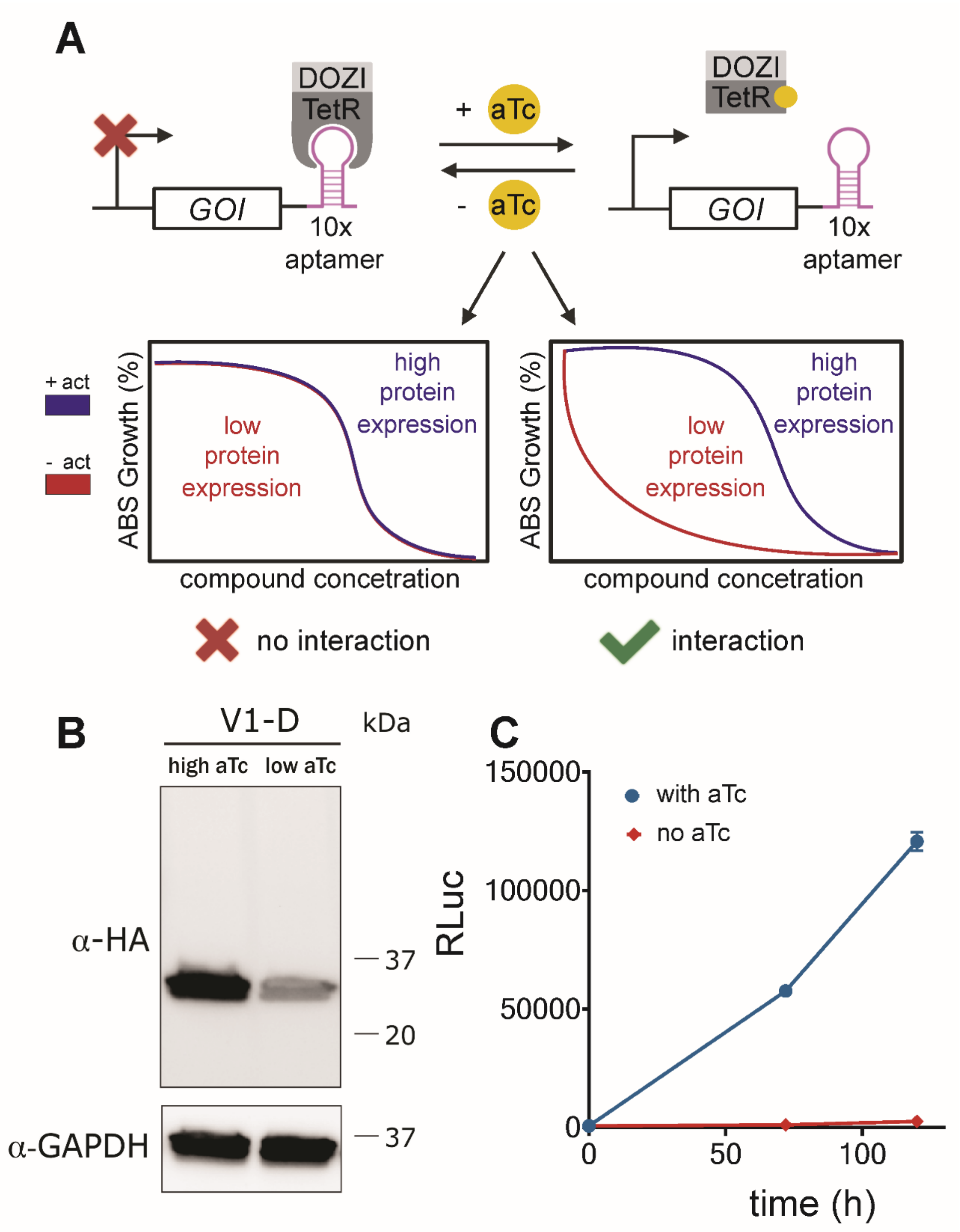

Supplementary Fig. 1: Conditional knockdown (cKD) of PfV1-D. (**A**) Schematic representation of protein translation regulation using the TetR-DOZI-RNA aptamer module. Figure was generated on BioRender software (https://www.biorender.com/). (**B**) Western blot assessment of the ~28 kDa PfV1-D protein using anti-HA antibodies that target the 3×HA tag introduced together with the cKD machinery. Protein expression was maintained in the presence of 500 nM anhydrotetracycline (aTc), contrasting with low levels upon aTC removal. GAPDH (PF3D7_1462800; ~37 kDa) was a loading control. (**C**) Renilla luciferase (RLuc) was integrated downstream of the cKD construct. The loss of its activity upon removal of aTC shows that PfV1-D expression is essential, contrasting with very high RLuc values in the presence of 500 nM aTc. Data represents mean of 2 biological repeats with technical duplicates.

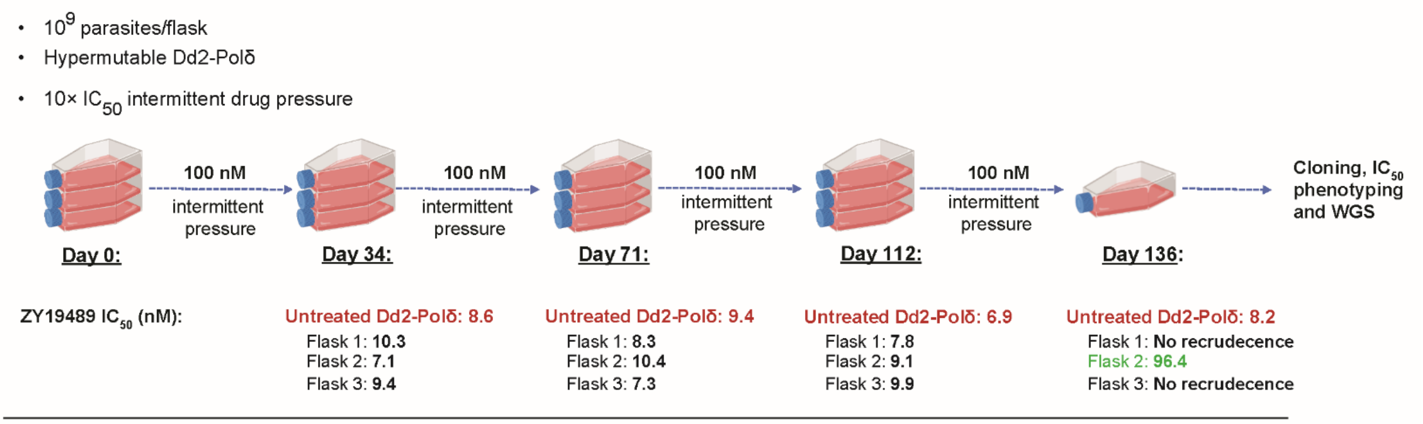

Supplementary Fig. 2: Schematic detailing timelines and experimental approach used to select a ZY19489-resistant mutant *P. falciparum* line. The Dd2-Polδ clone was set up in triplicate flasks with intermittent drug pressure corresponding to 10× IC_{50} (100 nM) and tracked over time. Parasites in drug-pressured flasks were tested for shifts in IC_{50} values on days 34, 71, 112 and 136, and each time compared to untreated Dd2-Polδ parent (IC_{50} values highlighted in red). The final IC_{50} shift indicating the presence of a resistant parasite population was observed on day 136 in parasites from flask 2 and is highlighted in green. No recrudescence was observed in ZY19489-pressured flasks 1 or 3 up to day 160, at which time selections were stopped. Figure was generated on BioRender software for biological images (https://www.biorender.com/).

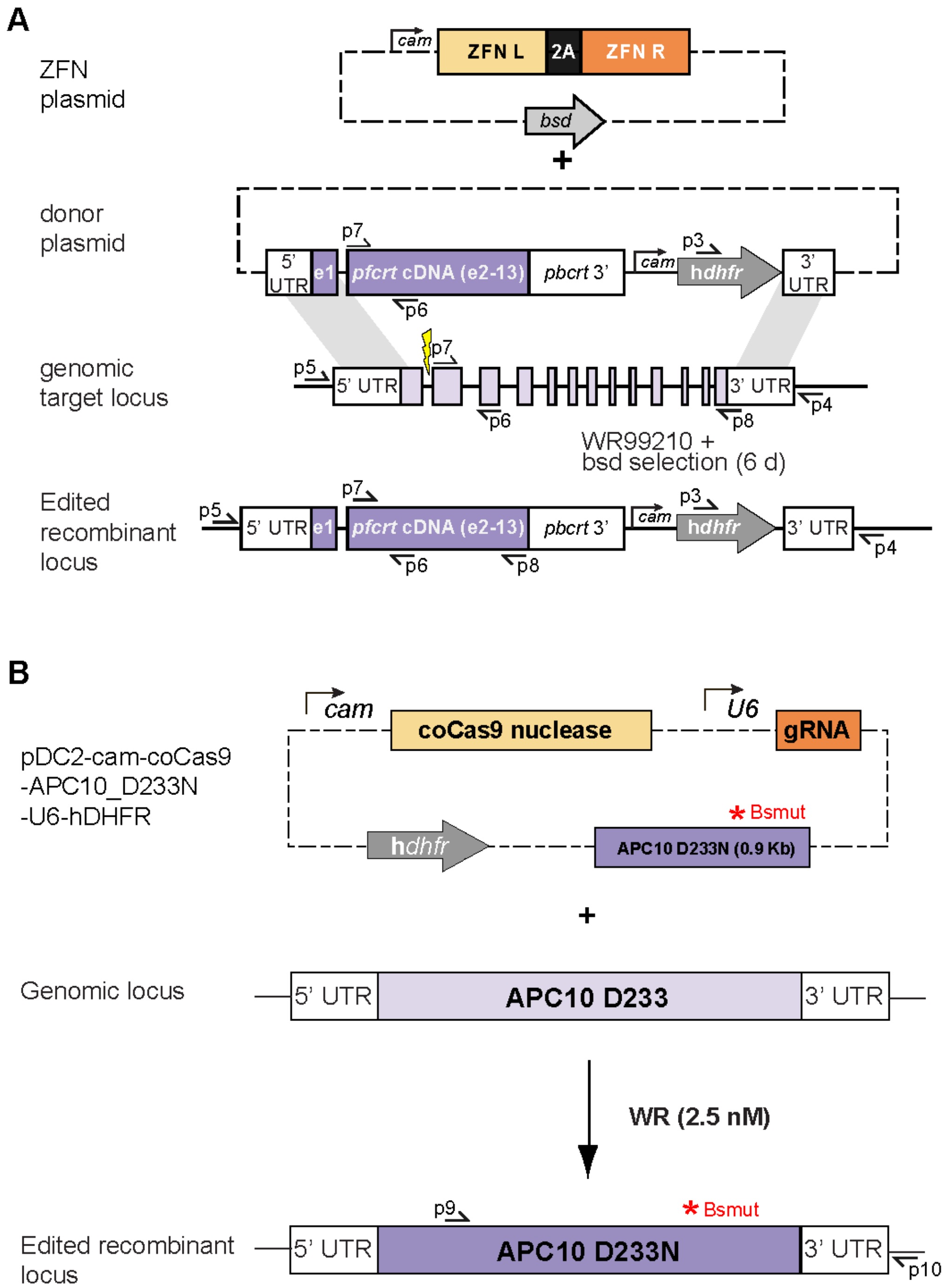

Supplementary Fig. 3: Schematic of *pfcrt* and *pfapc10* gene editing. (**A**) Zinc-Finger Nuclease (ZFN)-mediated editing of *pfcrt* used a two-plasmid approach: one containing the donor and one containing *pfcrt*-specific ZFNs. Parasites were selected for expression of the human *dihydrofolate reductase* (h*dhfr*) selectable marker with 2.5 nM of the *P. falciparum* DHFR-specific inhibitor WR99210 and expression of the blasticidin S-deaminase selectable marker with a 6-day pulse of 2 µg/ml blasticidin hydrochloride. (**B**) Editing of *pfapc10* was achieved using a CRISPR/Cas9 approach, including a homologous donor sequence harboring the mutant 233N codon. This donor fragment also contained synonymous mutations in the gRNA recognition site in the chromosomal locus, shown as the Bsmut site, which precluded further Cas9 editing of the edited locus.

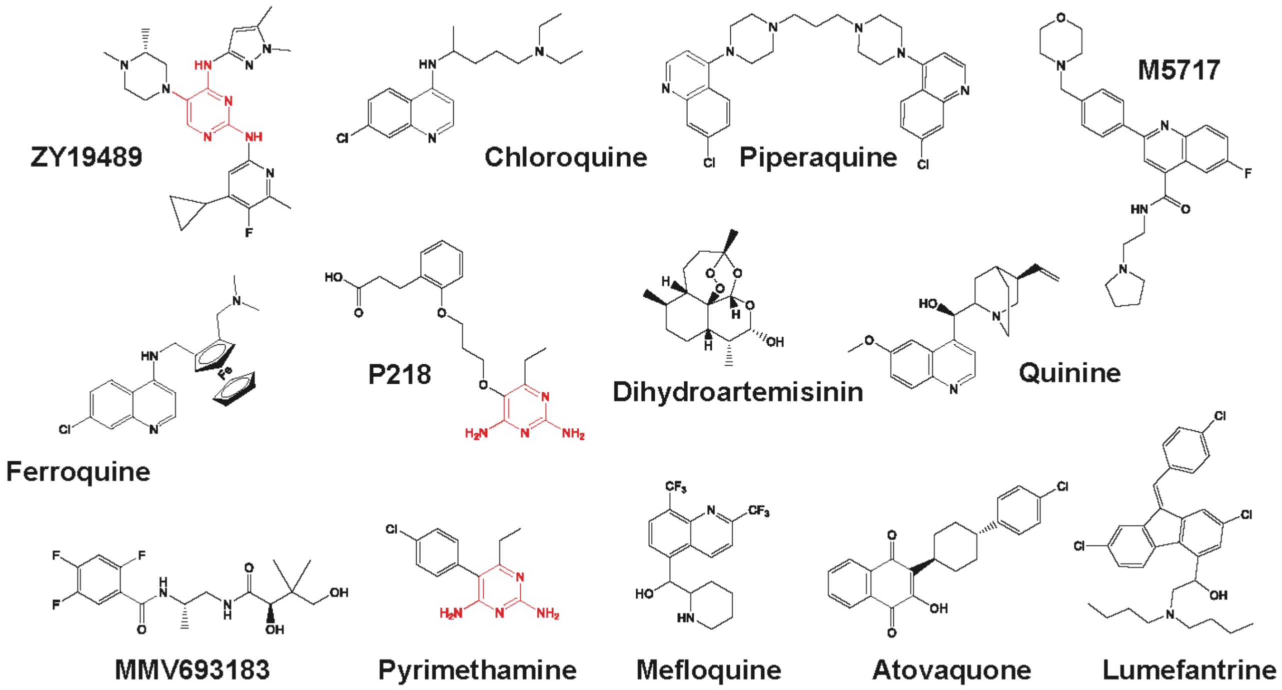

Supplementary Fig. 4: Chemical structures of antimalarial compounds included in this study. The red substructure illustrates the aminopyrimidine scaffold in ZY19489 and shared by the *P. falciparum* dihydrofolate reductase (DHFR) inhibitors P218 and pyrimethamine.

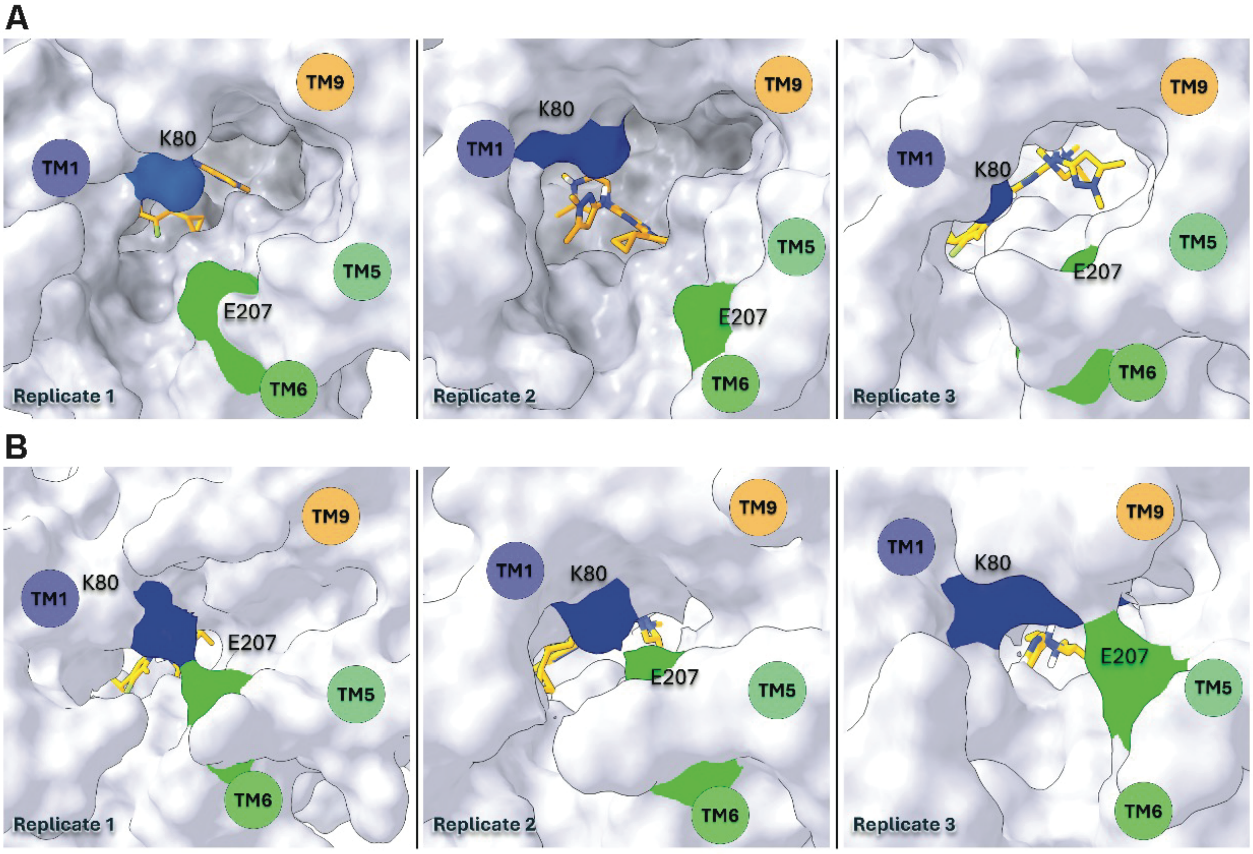

Supplementary Fig. 5: Molecular dynamics simulations showing intramolecular configurations for (A) 7G8 (parental) and (B) 7G8+N246H (mutant) PfCRT isoforms that differentially impact transporter interactions with ZY19489. The ZY19489 structure is shown as a stick figure). (**A**) Surface representations of 7G8 PfCRT, across three simulation replicates, indicate that the cavity remains open, facilitating ligand entry or exit. (**B**) The 7G8+N246H variant consistently transitions to a closed-to-DV conformation, marked by the formation of a salt bridge between residues K80 and E207 or nearby residues. We postulate that this modified configuration in the 7G8+N246H variant results in restricted access of ZY19489 to the drug binding and transport cavity.

A. Open-to-DV 7G8 PfCRT

Replicate 1

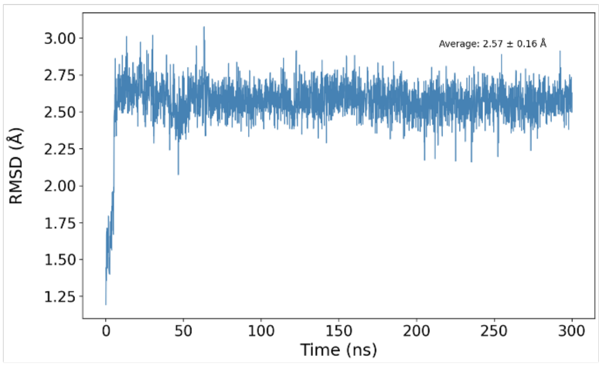

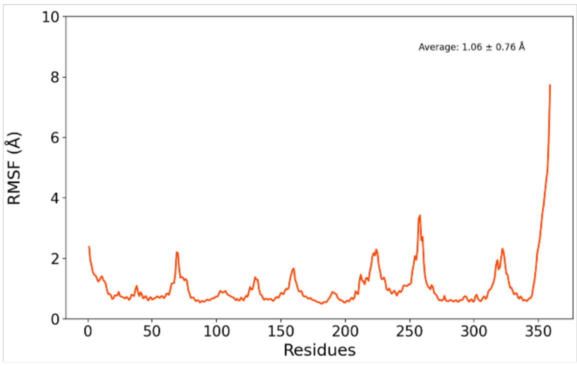

Replicate 2

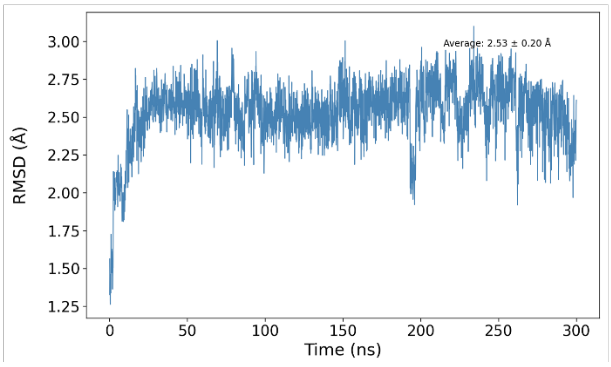

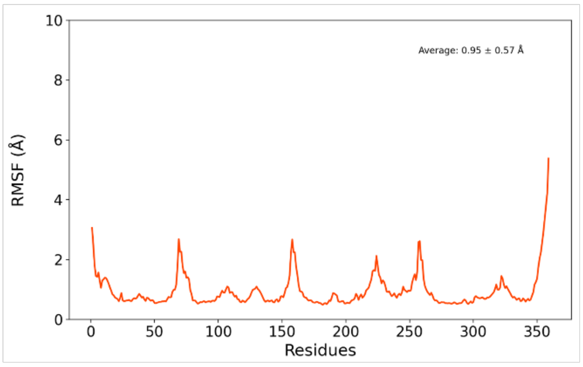

Replicate 3

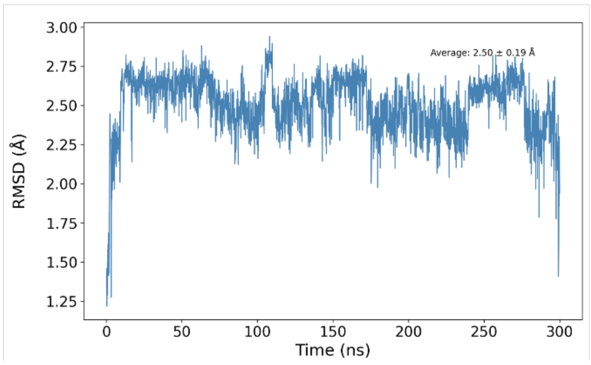

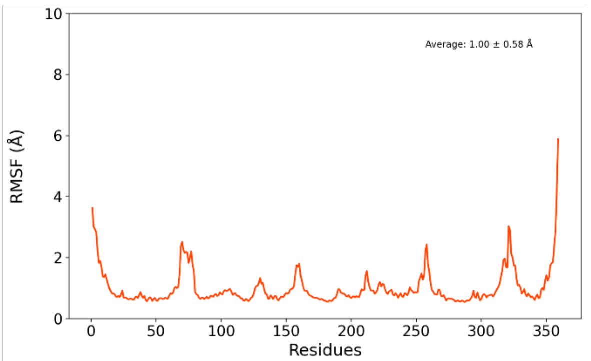

B. Open-to-cytosol 7G8 PfCRT

Replicate 1

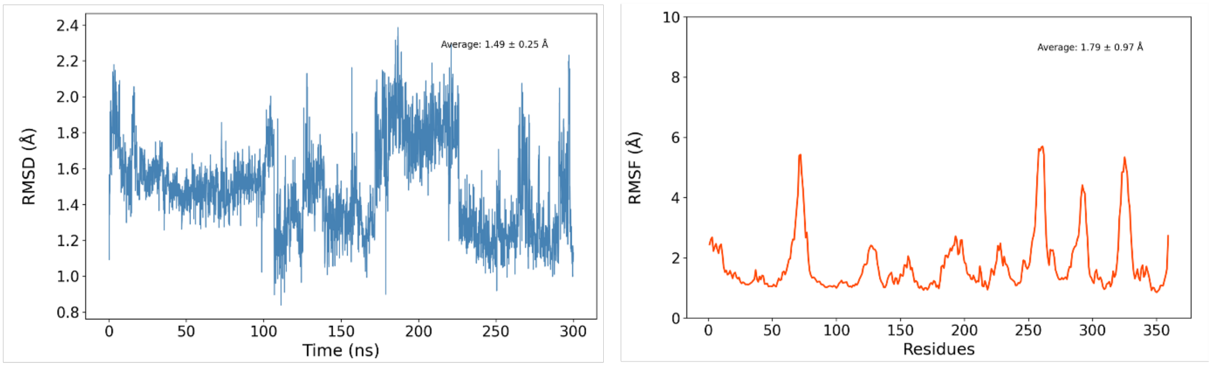

Replicate 2

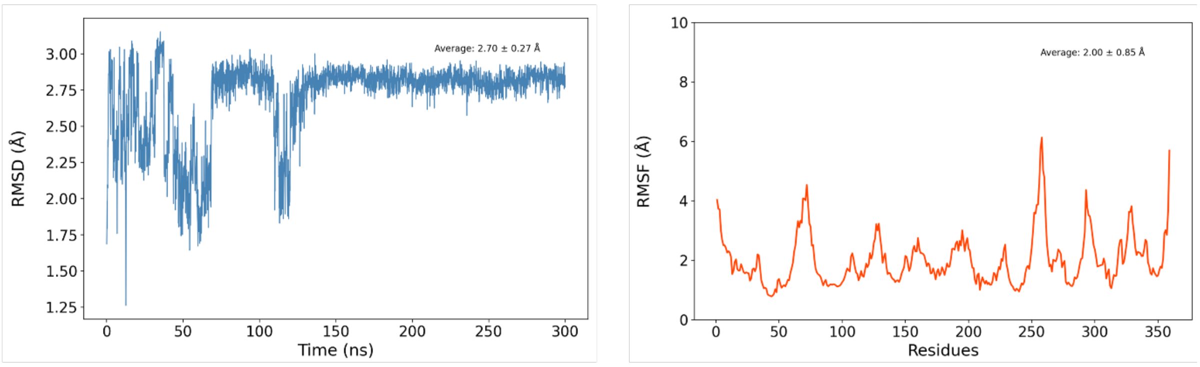

Replicate 3

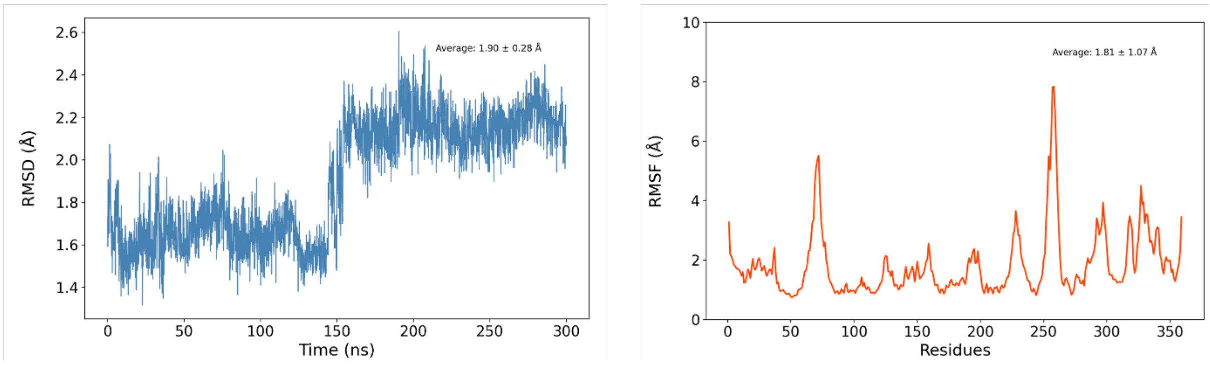

C. Open-to-DV 7G8+N246H PfCRT

Replicate 1

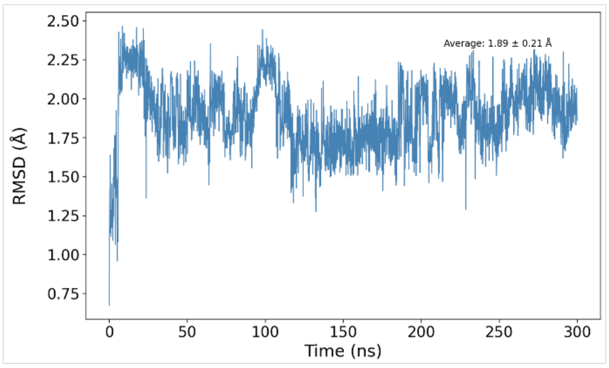

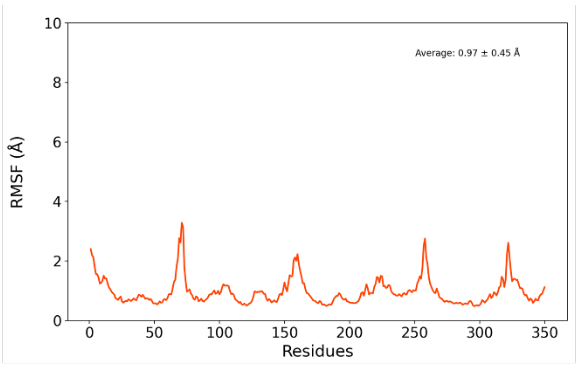

Replicate 2

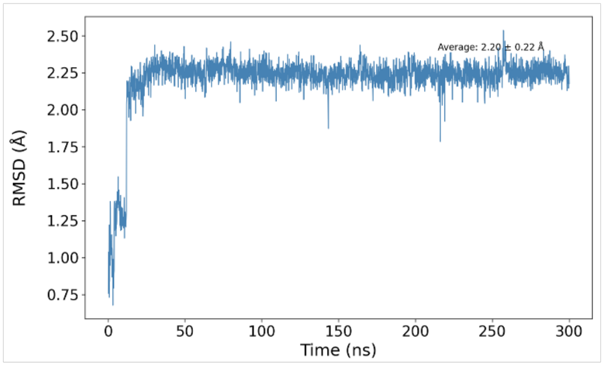

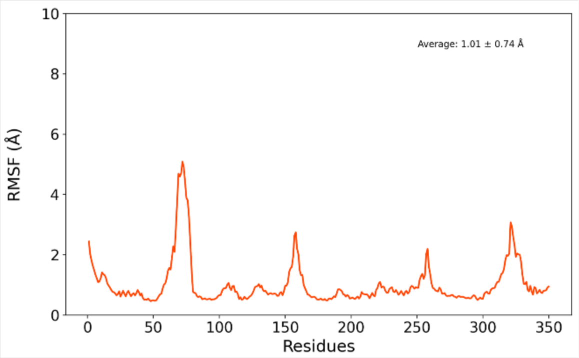

Replicate 3

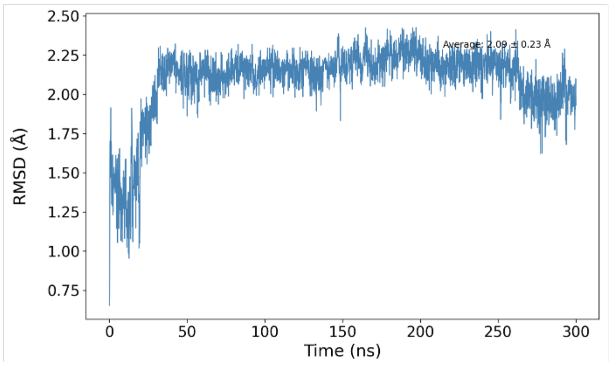

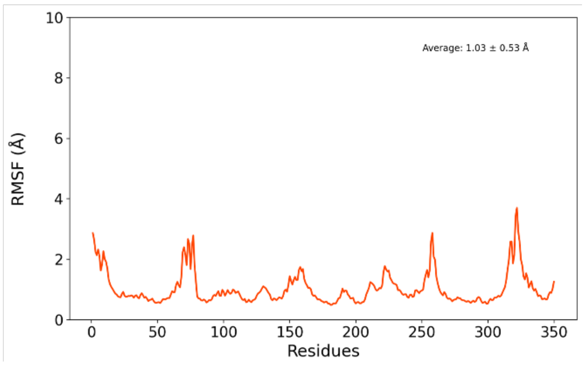

D. Open-to-cytosol 7G8+N246H PfCRT

Replicate 1

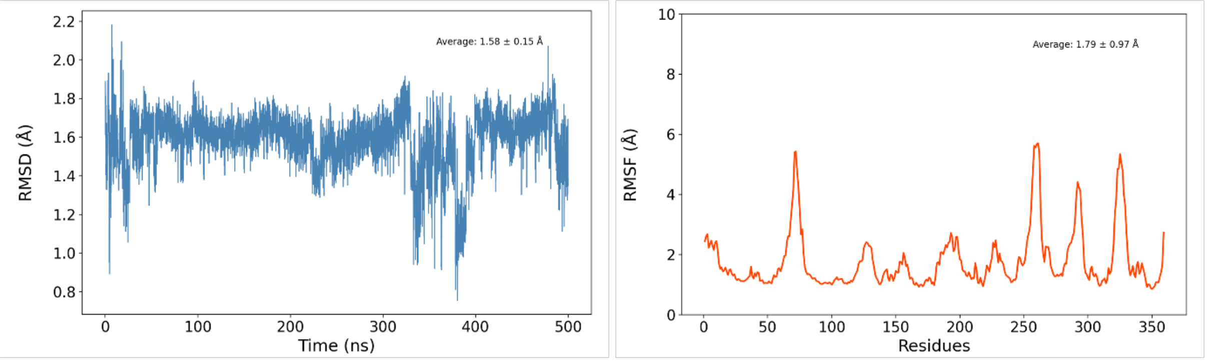

Replicate 2

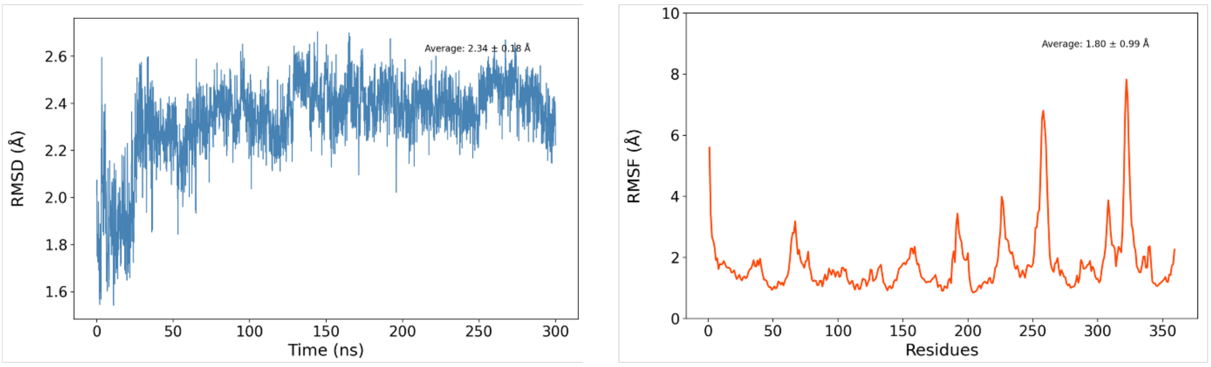

Replicate 3

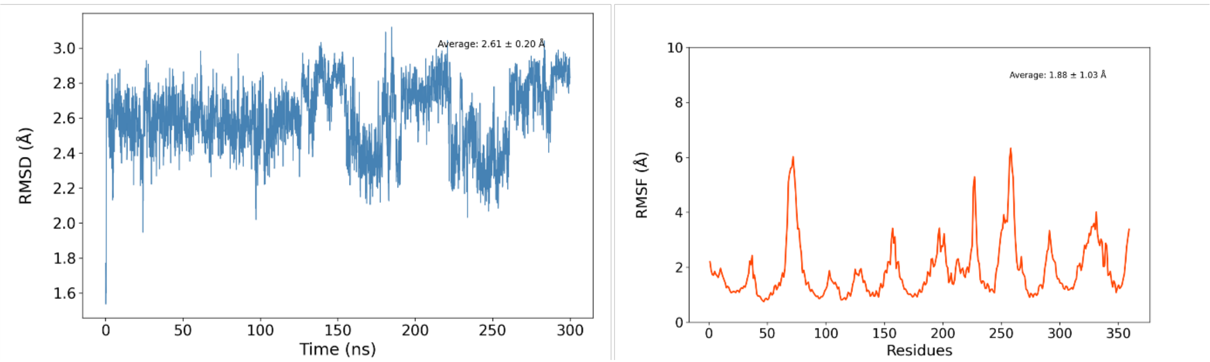

E. Open-to-DV 7G8 PfCRT (distance between ZY19489-S140 and ZY19489-D326)

Replicate 1

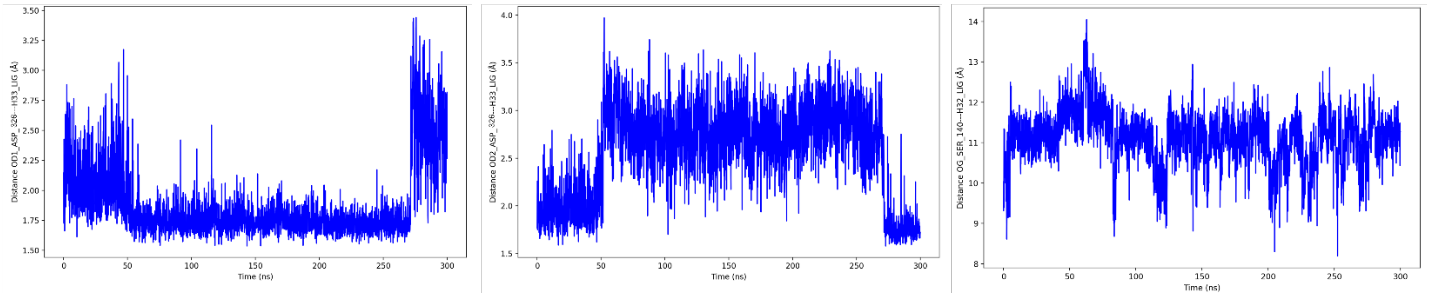

Replicate 2

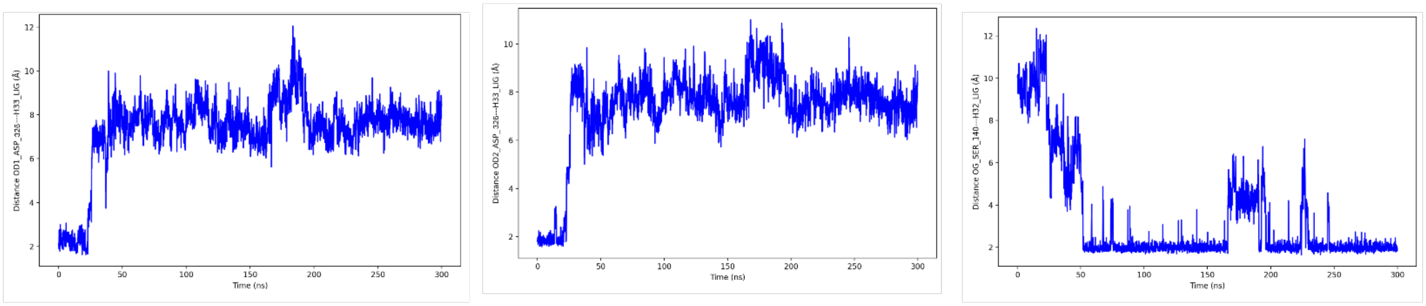

Replicate 3

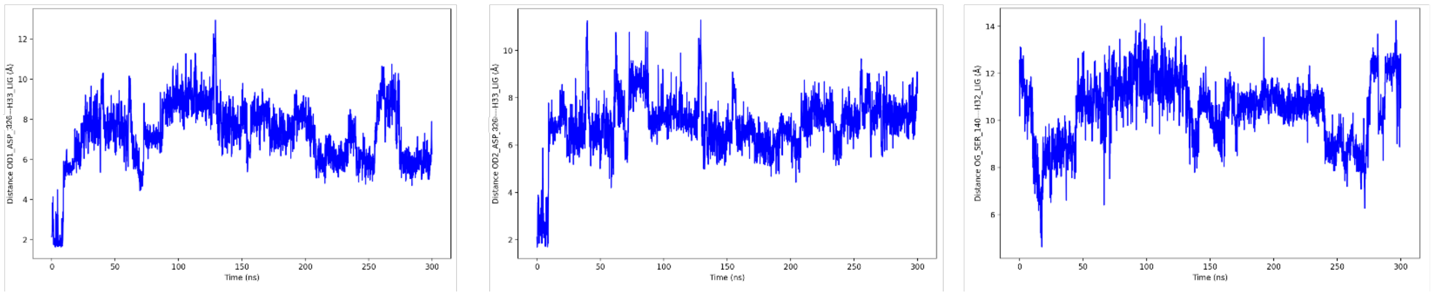

F. K80-E207 interaction Open-to-DV 7G8 PfCRT

Replicate 1

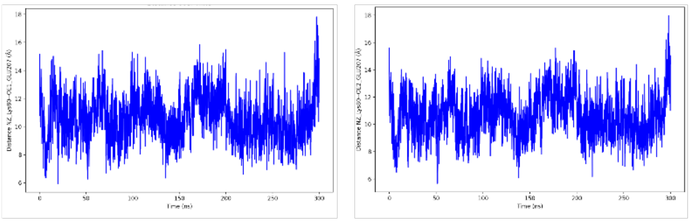

Replicate 2

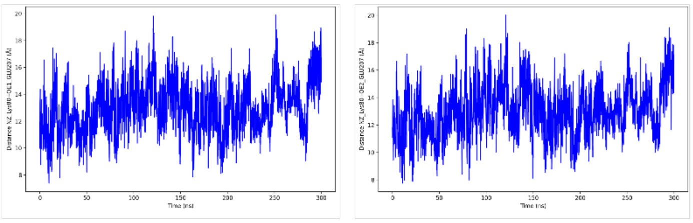

Replicate 3

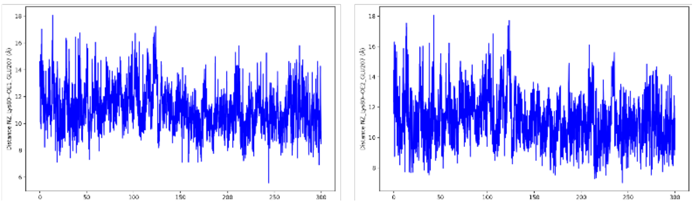

G. K80-E207 interaction Open-to-DV 7G8+N246H PfCRT

Replicate 1

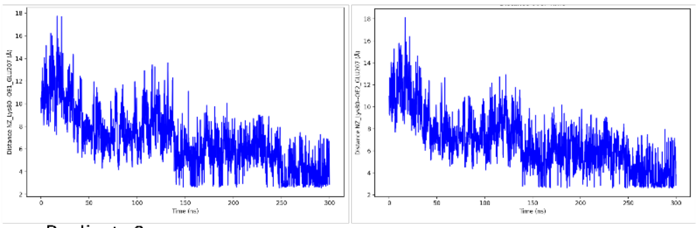

Replicate 2

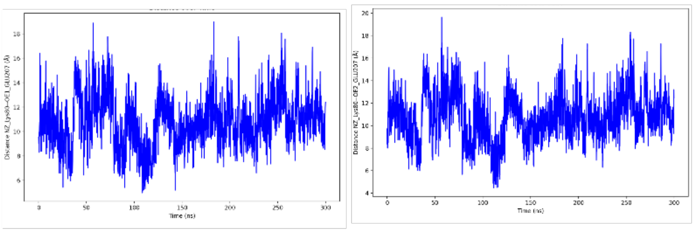

Replicate 3

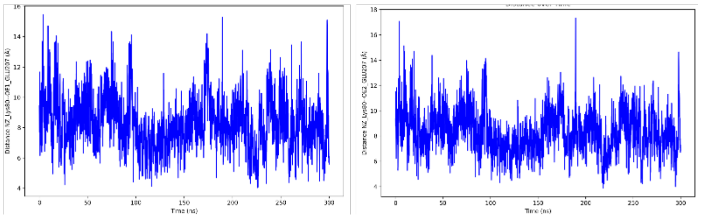

H. TM1-TM4, TM4-TM9, TM5-TM9 interactions Open-to-DV 7G8 PfCRT

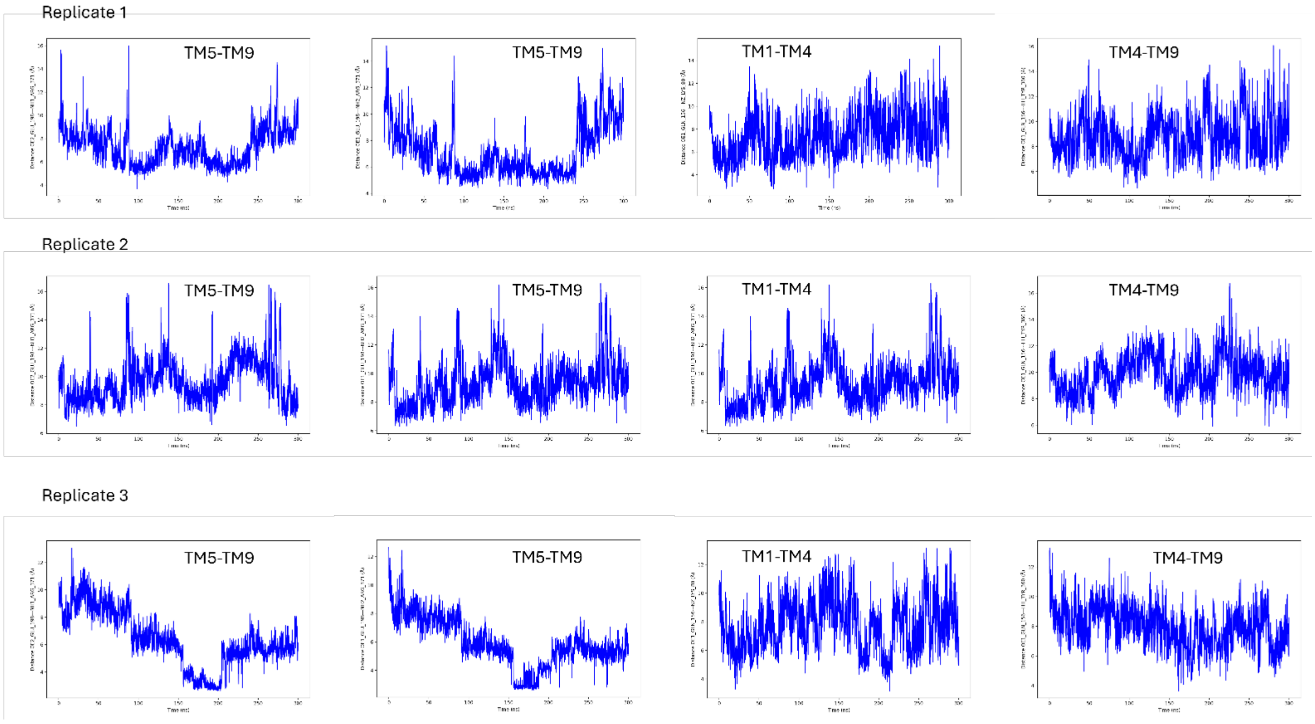

I. TM1-TM4, TM4-TM9, TM5-TM9 interactions Open-to-DV 7G8+N246H PfCRT

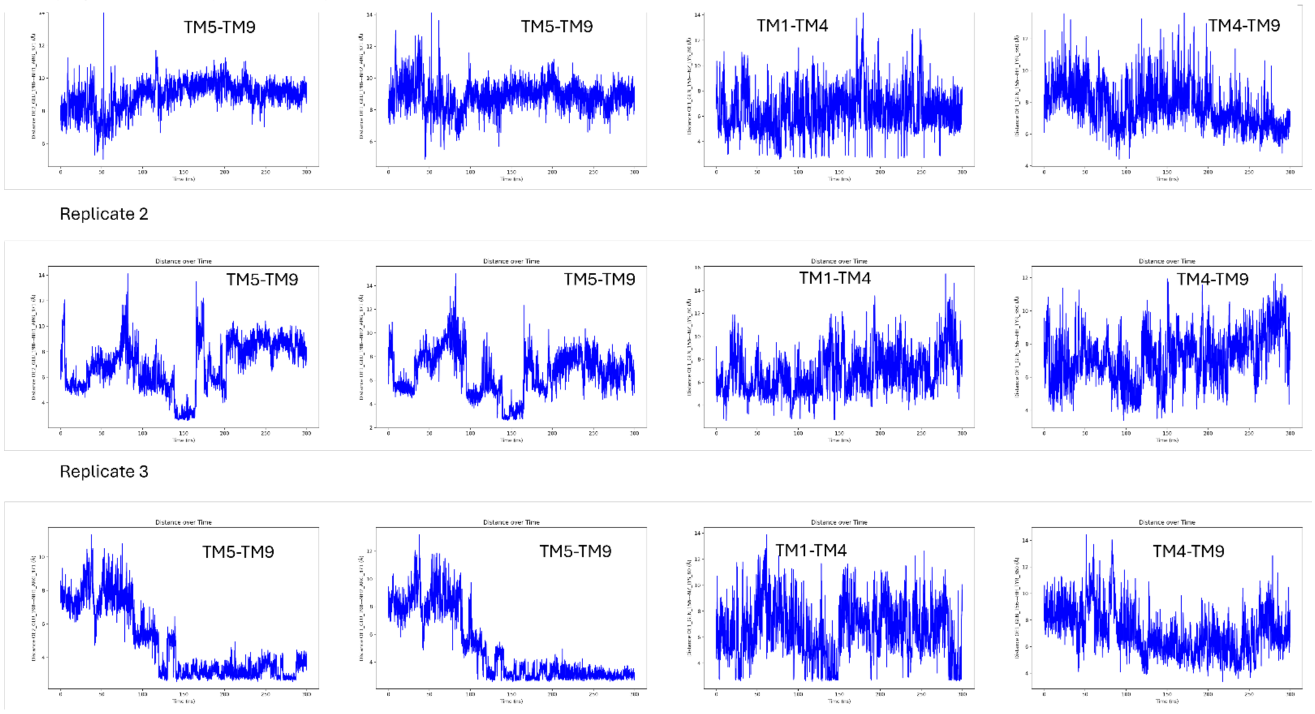

J. Open-to-cytosol 7G8 PfCRT (distance between ZY19489-E232 and ZY19489-L245

Replicate 1

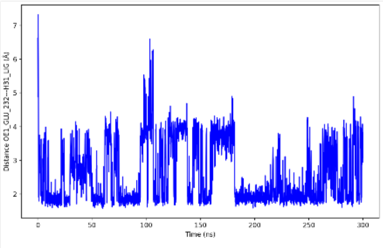
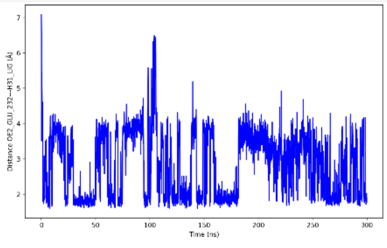
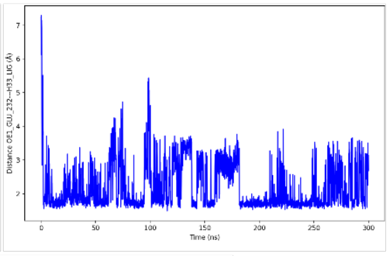
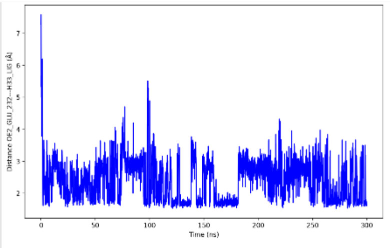
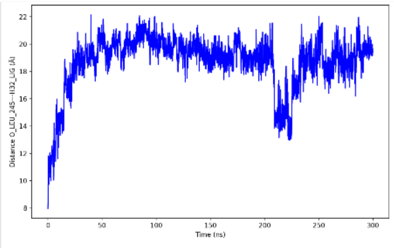

Replicate 2

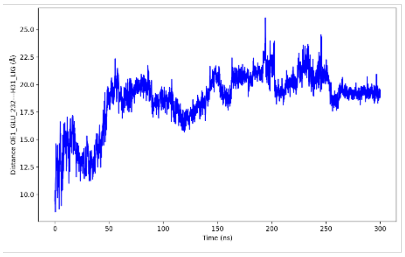
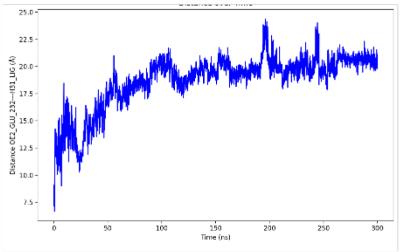
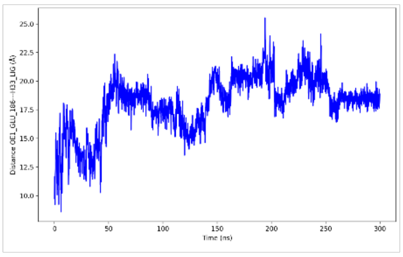
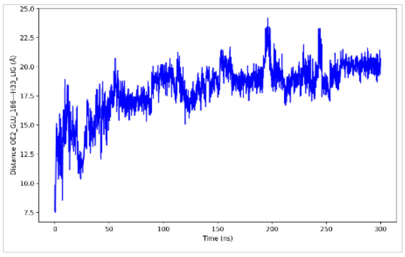
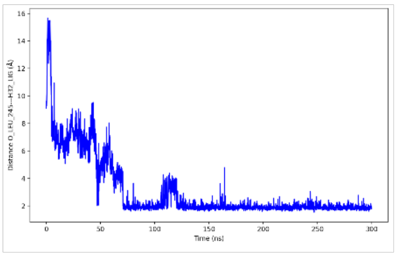

Replicate 3

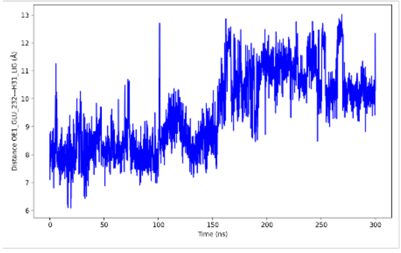
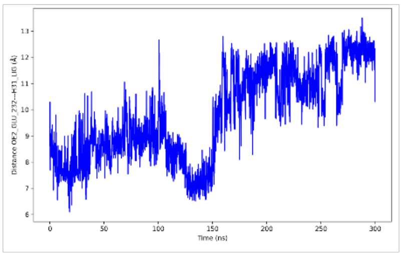
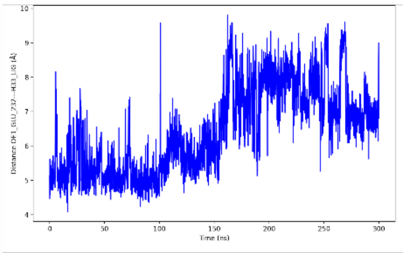
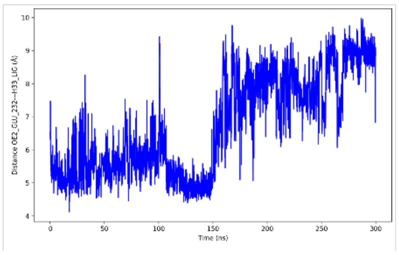
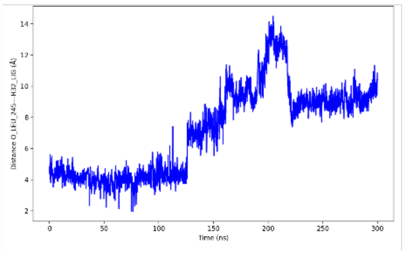

K. Open-to-cytosol 7G8+N246H PfCRT – distance between ZY19489 and Q161

Replicate 1

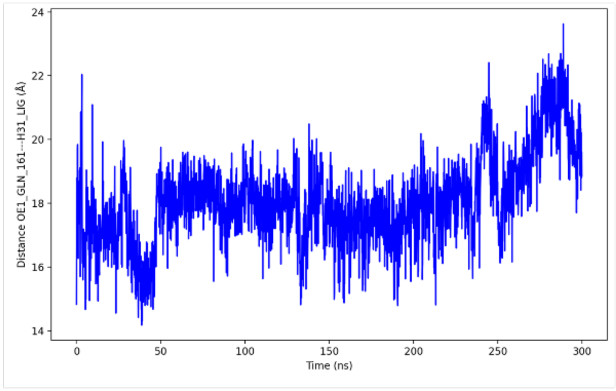

Replicate 2

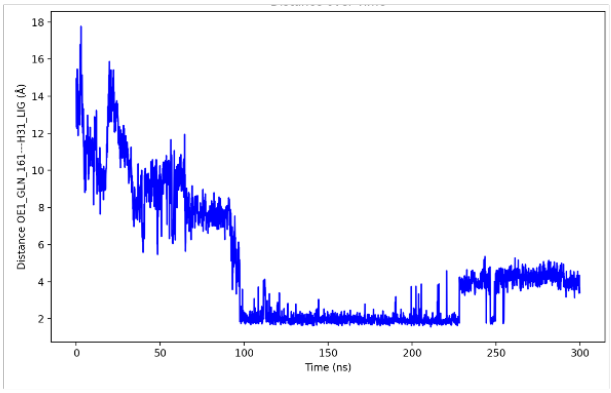

Replicate 3

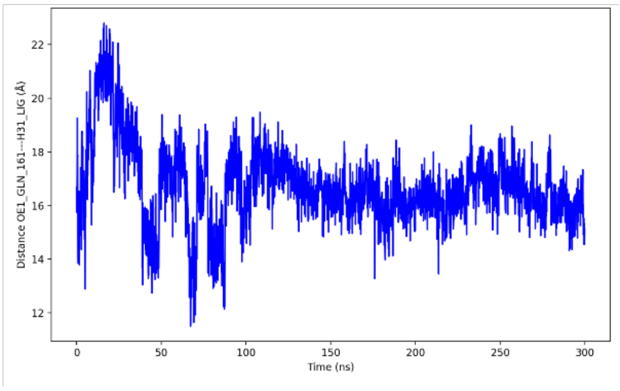

Supplementary Fig. 6: Molecular dynamics simulations with 7G8 and 7G8+N246H PfCRT. (**A-D)** Root Mean Square Deviation (RMSD) of ZY19489 (left) and Root Mean Square Fluctuation (RMSF) of the protein (right) for 7G8 and 7G8+N246H PfCRT in different conformational states over 300 ns of molecular dynamics (MD) simulations. (**A**) 7G8 PfCRT in the open-to-DV conformation; **(B)** 7G8 PfCRT in the open-to-cytosol conformation; **(C)** 7G8+N246H PfCRT in the open-to-DV conformation; and (**D**) 7G8+N246H PfCRT in the open-to-cytosol conformation. (**E**) Distances between ZY19489 and residues D326 and S140 in the open-to-DV conformation of 7G8 PfCRT over 300 ns of MD simulations. (**F-G**) Distance between residues K80 (NZ) and E207 (OE1 and OE2) in the open-to-DV conformation of PfCRT. (**F**) 7G8 PfCRT and (**G**) 7G8+N246H mutant PfCRT over 300 ns of MD simulations. (**H**-I) Distances between residues involved in interactions that contribute to closing the channel in the open-to-DV conformation. The key residues include R371 (NH1 and NH2, TM9), E152 (OE1 and OE2, TM5), K80 (NZ, TM1), Q156 (OE1, TM4), and Y360 (HH, TM9). (**H**) Open-to-DV 7G8 PfCRT; and (**I**) Open-to-DV 7G8+N246H PfCRT, illustrating the bridging of regions between TM1-TM4, TM5-TM9, and TM4-TM9. (**J**) Distances between ZY19489 and key residues E232 and L245 in the open-to-cytosol conformation of 7G8 PfCRT. (**K**) Distance between ZY19489 and Q161 in the open-to-cytosol conformation of 7G8 PfCRT. Results are shown for three independent replicates per MD simulation.

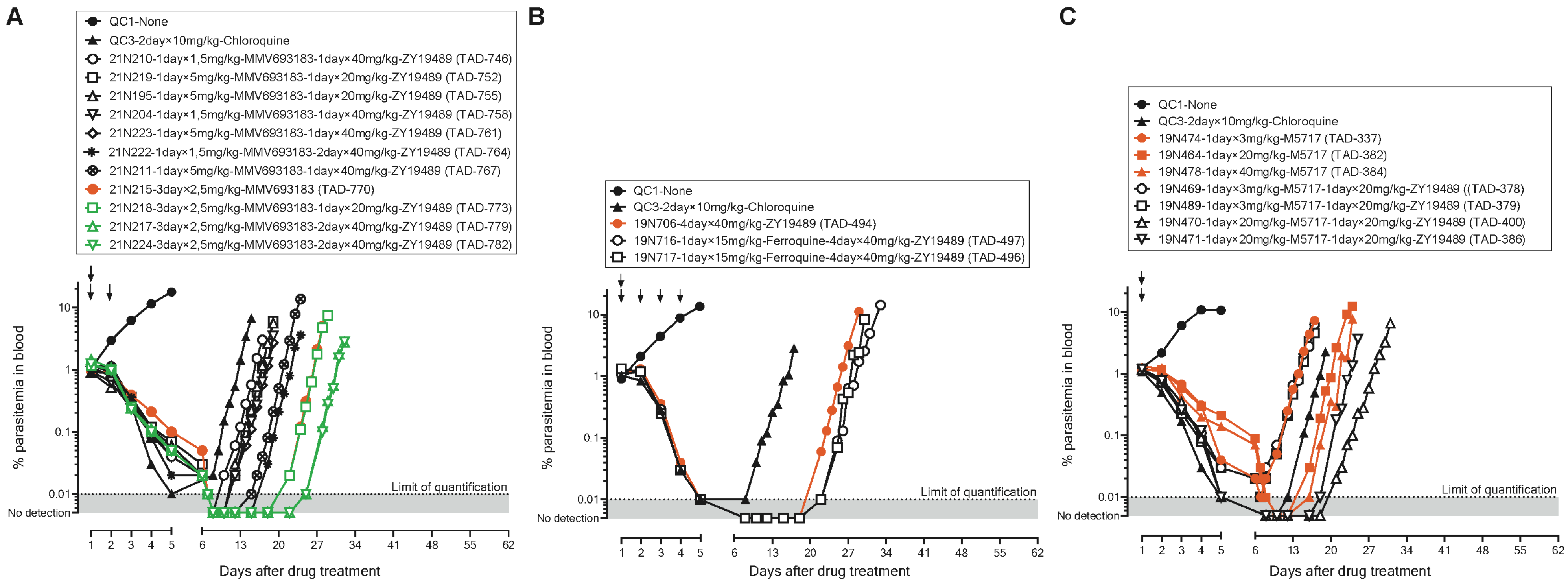

Supplementary Fig. 7: Parasitemia profiles in NSG mice engrafted with human erythrocytes (TADhuMouse®) treated with ZY19489-based combinations. (A-C) Percentage of total erythrocytes infected with asexual *Plasmodium falciparum* stages (parasitemia) measured in peripheral blood of NSG mice engrafted with human erythrocytes (TADhuMouse®), expressed relative to the total erythrocyte population (murine + human), from three independent therapeutic studies. The *P. falciparum* strain used was Pf3D7$^{0087/N9}$, a reference strain routinely used for antimalarial evaluation in the TADhuMouse® model. Mice with ~1.2% total parasitemia at treatment initiation received once-daily oral dosing (q24h; 10 mL/kg) of ZY19489 co-administered with (**A**) MMV693183, (**B**) ferroquine, or (**C**) M5717. Each combination was co-formulated as a single suspension in double-distilled water containing 1% methylcellulose and 0.1% Tween-80. The black arrows in the plots indicate the days of drug administration. Animals included in these panels were selected for parasite genomic analysis and represent subsets of individuals exhibiting pronounced recrudescence (days of recrudescence, DoR) within larger efficacy studies (11 out of 15, 3 out of 19 and, 7 out of 16, from each combination studies, respectively) . The efficacy studies were intentionally designed not to achieve cure to enable the assessment of mutations emerging in recrudescent parasites. Each plot includes a chloroquine-treated and an untreated mouse used as quality controls for inter-assay comparison in each study. Whole-genome sequencing was performed for the corresponding recrudescent parasite sample from each individual (indicated in parentheses), and a subset was additionally evaluated for compound susceptibility *in vitro* (data shown in **Table 2**).

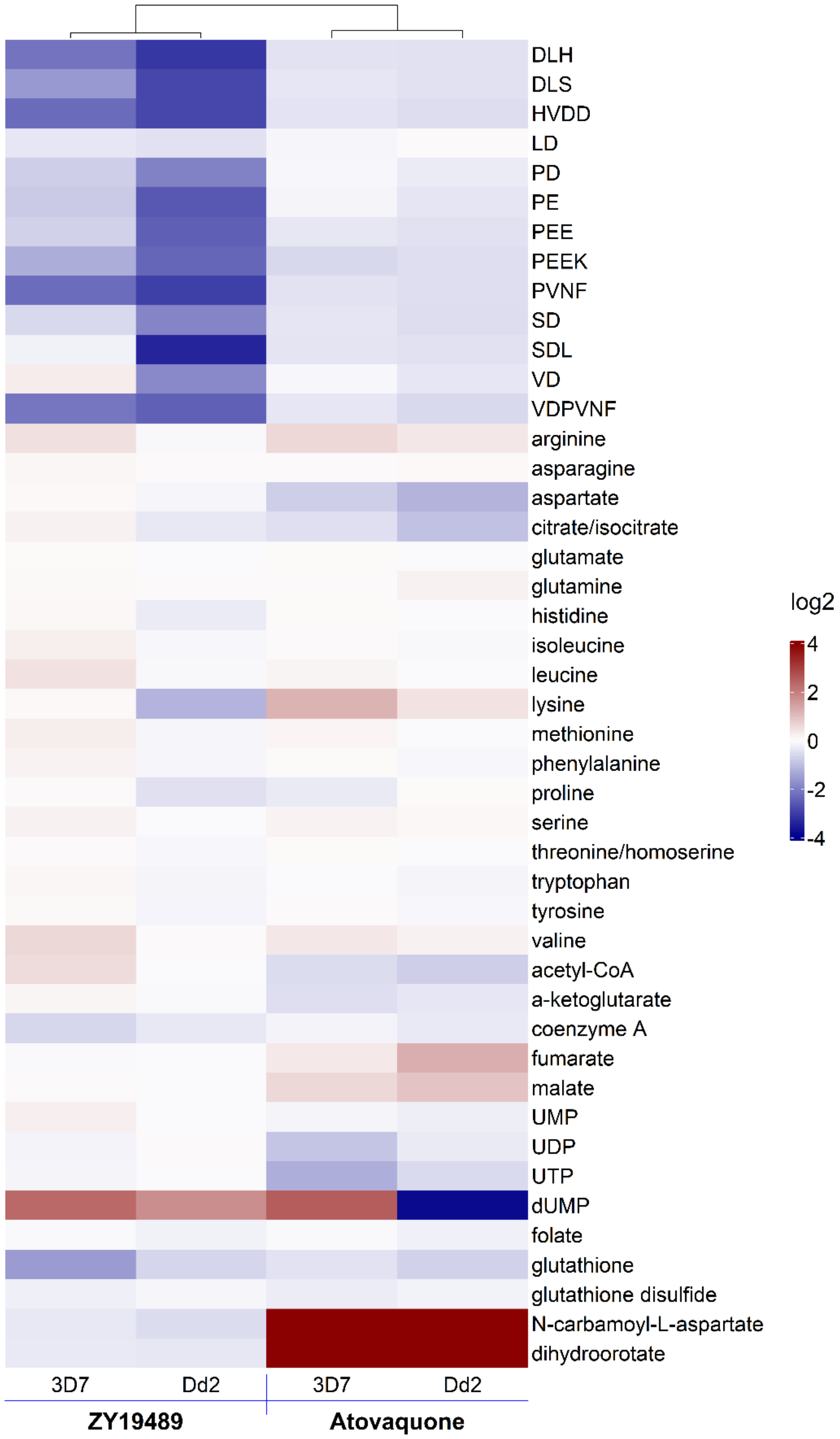

Supplementary Fig. 8: Heatmap of log2 fold changes of 3D7 and Dd2 treated with ZY19489 or atovaquone. The data were filtered to only include mitochondrial metabolites, peptides, amino acids, and metabolites from folate biosynthesis and central carbon metabolism. The complete list of all metabolites detected is presented in **Table S12**.

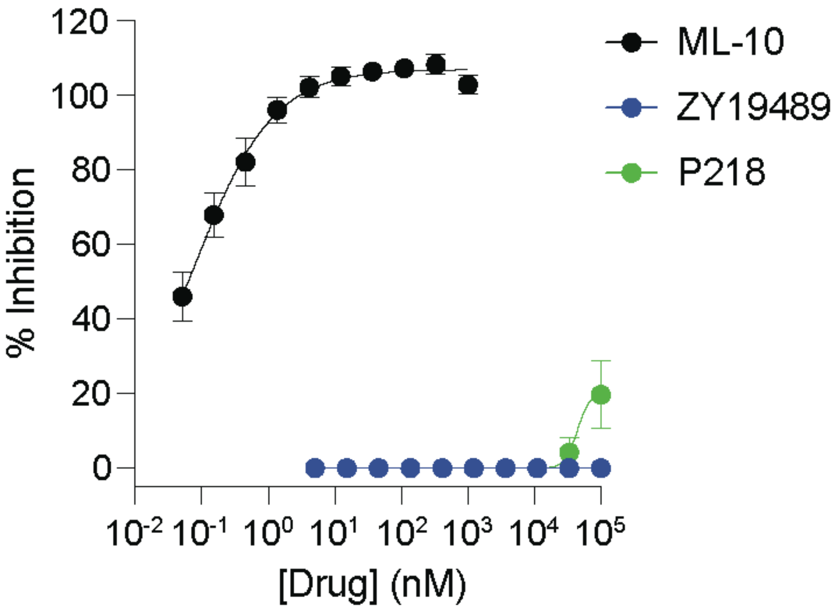

Compound	Mean IC_{50} ± SEM (nM)	Mean IC_{90} ± SEM (nM)
ZY19489	>100, 000	NA
ML-10	0.09 ± 0.015	0.913 ± 0.35
P218	>100, 000	NA

Supplementary Fig. 9: Inhibition of recombinant *P. falciparum* cGMP-dependent protein kinase (PfPKG). Measurements of inhibition were based on the ADP-Glo Kinase Assay (Promega) that measures ADP formation. A Spectromax plate reader was used to measure luminescent signals from PfPKG protein incubated with the specific inhibitor ML-10 (black), ZY19489 (blue), or the parasite DHFR inhibitor P218 (green). Data were normalized based on the 100% activity controls (1% DMSO only) and the 100% inhibition controls (10 µM kinase inhibitor Staurosporine). Mean IC_{50} and IC_{90} values were calculated from 3 independent experiments with 4 technical repeats (*N*, n = *3*, 4). NA, not applicable.

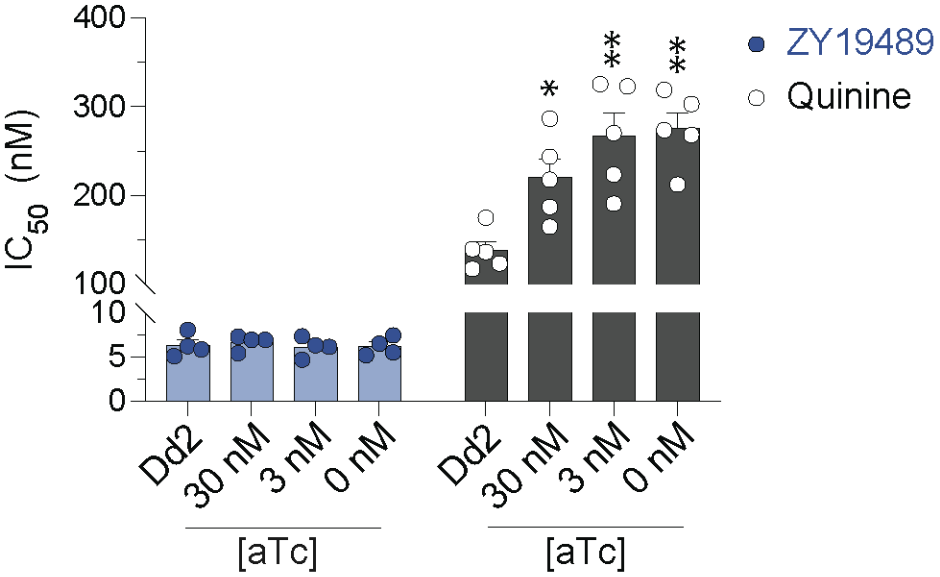

Supplementary Fig. 10: PfHDP expression levels do not impact ZY19489 activity against *P. falciparum* asexual blood stages. Prior data have suggested that PfHDP (*P. falciparum* hemozoin depolymerization protein) might impact production of hemozoin and by corollary, the activity of antiplasmodial compounds that interfere with heme detoxification. Using a strategy similar to that employed for PfV1-D, we generate a conditional knockdown of PfHDP (cKD PfDHP) in Dd2 parasites. These parasites were then cultured in 30 nM, 3 nM or 0 nM aTC to generate different levels of protein knockdown. 72-hour dose-response assays were performed on 4 to 5 separate occasions with technical duplicates. Results are shown as means ± SEM. The data showed no change in the ZY19489 IC_{50} value across a range of PfHDP expression levels, consistent with this protein not impacting ZY19489 potency. Quinine showed a statistically significant increase in IC_{50} values as PfHDP levels were decreased, suggesting that PfHDP expression might contribute to its activity.

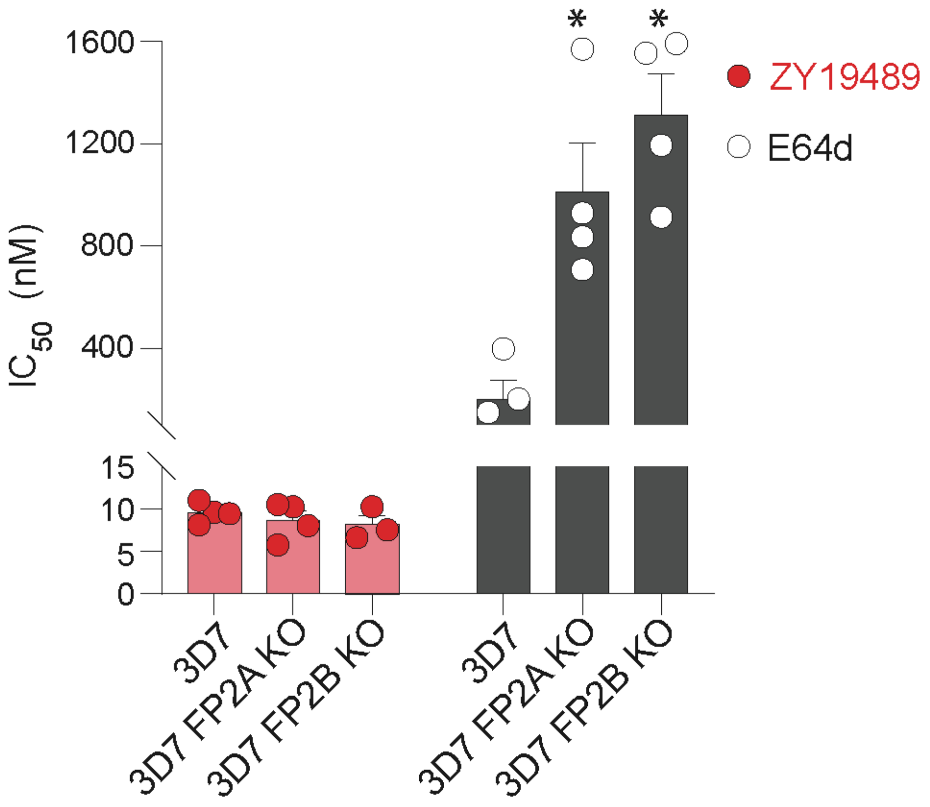

Supplementary Fig. 11: Evidence that falcipain 2A or 2B do not modulate parasite susceptibility to ZY19489. Data show mean ± SEM IC_{50} values for ZY19489 tested against the 3D7 parental line or isogenic lines in which falcipain 2A (FP2A) or 2B (FP2B) were deleted. As a comparator, we tested E64d, whose mode of action is thought to include falcipain 2. Here, lines deleted in FP2A or FP2B showed a gain of low-level resistance. Statistical significance was determined using Mann-Whitney *U* tests. $^{*}p < 0.05$.

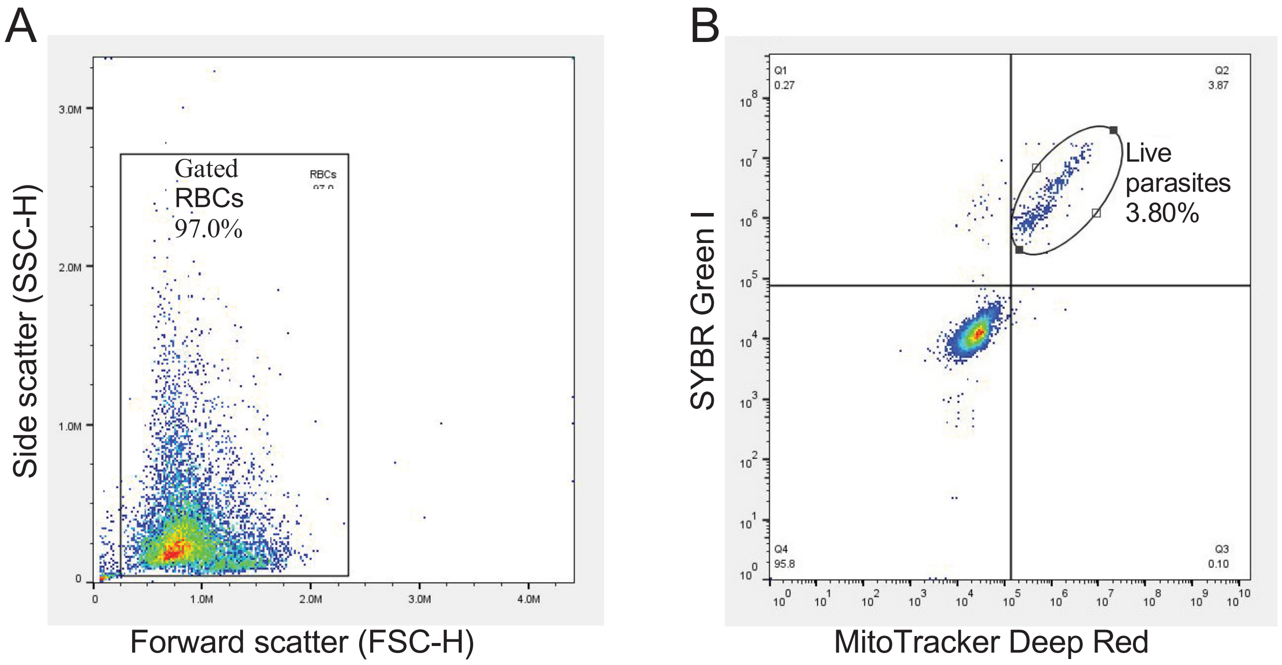

Supplementary Fig. 12: Gating strategy for flow cytometry-based quantification of *P. falciparum* asexual blood stage parasite proliferation in drug susceptibility and growth rate assays. Intra-erythrocytic parasites were seeded at 0.3% - 0.8% parasitemia and 1% hematocrit for 72 hours in the presence of a range of drug concentrations that had been 2-fold serially diluted in duplicates along with drug-free controls. Cells were then labeled with 1× SYBR Green (Invitrogen) and 100 nM MitoTracker Deep Red FM (Invitrogen) as nuclear stain and vital dyes, respectively. Parasite survival was assessed by flow cytometry on an Intellicyt iQue3 (Essen Bioscience). Between 9,000 and 15,000 events were counted per sample. Data shows control gating of untreated parasite cultures from a representative dose-response assay. (**A**) Forward (FSC-H) and side scatter (SSC-H) gating of the total red blood cell (RBC) population, representing 97.0% of the total events counted. (**B**) Gating strategy used to quantify viable intracellular parasites, which are positively stained by both MitoTracker Deep Red and SYBR Green I and appear as events in the upper right gate (live parasites, 3.80% parasitemia). Uninfected erythrocytes appear in the lower left gate as MitoTracker Deep Red and SYBR Green I negative populations.

Supplementary Table 1: Haplotypes of known drug resistance genes of the edited and control *P. falciparum* lines used in this study.

			PfCRT haplotype												PfMDR1 haplotype		PfDHFR haplotype	*Pfpm2/3* copies
**Parasite line**	**Origin**	**Edited**	**72**	**74**	**75**	**76**	**145**	**220**	**246**	**271**	**326**	**343**	**356**	**371**	**86·184·1246**	**Gene copies**	**16·51·59·108·164**	
Dd2	S.E. Asia	No	C	**I**	**E**	**T**	F	**S**	N	**E**	**S**	M	**T**	**I**	**Y**YD	2	A**IRN**I	1
Dd2-Polδ		Yes	C	**I**	**E**	**T**	F	**S**	N	**E**	**S**	M	**T**	**I**	**Y**YD	2	A**IRN**I	1
Dd2$^{\text{Dd2crt}}$		Yes	C	**I**	**E**	**T**	F	**S**	N	**E**	**S**	M	**T**	**I**	**Y**YD	2	A**IRN**I	1
Dd2$^{\text{Dd2crt+N246H}}$		Yes	C	**I**	**E**	**T**	F	**S**	**H**	**E**	**S**	M	**T**	**I**	**Y**YD	2	A**IRN**I	1
Dd2$^{\text{Dd2crt+M343L}}$		Yes	C	**I**	**E**	**T**	F	**S**	N	**E**	**S**	**L**	**T**	**I**	**Y**YD	2	A**IRN**I	1
Dd2$^{\text{GB4crt}}$		Yes	C	**I**	**E**	**T**	F	**S**	N	**E**	N	M	I	**I**	**Y**YD	2	A**IRN**I	1
Dd2$^{\text{GB4crt+N246H}}$		Yes	C	**I**	**E**	**T**	F	**S**	**H**	**E**	**S**	M	**T**	**I**	**Y**YD	2	A**IRN**I	1
Dd2^{FCBcrt}		Yes	C	**I**	**E**	**T**	F	**S**	N	**E**	**S**	M	I	**I**	**Y**YD	2	A**IRN**I	1
Dd2$^{\text{FCBcrt+N246H}}$		Yes	C	**I**	**E**	**T**	F	**S**	**H**	**E**	**S**	M	**I**	**I**	**Y**YD	2	A**IRN**I	1
Dd2$^{\text{Dd2crt+apc10D233N}}$		Yes	C	**I**	**E**	**T**	F	**S**	N	**E**	**S**	M	**T**	**I**	**Y**YD	2	A**IRN**I	1
Dd2$^{\text{HDP cKD}}$		Yes	C	**I**	**E**	**T**	F	**S**	N	**E**	**S**	M	**T**	**I**	**Y**YD	2	A**IRN**I	1
FCB	S.E. Asia	No	C	**I**	**E**	**T**	F	**S**	N	**E**	**S**	M	I	**I**	**SN**D	2	**T**NC**T**I	1
FCB-KD$^{\text{mdr1}}$		Yes	C	**I**	**E**	**T**	F	**S**	N	**E**	**S**	M	I	**I**	**SN**D	1	**T**NC**T**I	1
3D7	Africa	No	C	M	N	K	F	A	N	Q	N	M	I	R	NYD	1	ANCSI	1
3D7$^{\text{0087/N9}}$		Yes	C	M	N	K	F	A	N	Q	N	M	I	R	NYD	1	ANCSI	1
NF54	Africa	No	C	M	N	K	F	A	N	Q	N	M	I	R	NYD	1	ANCSI	1
NF54$^{\text{V1-D cKD}}$		Yes	C	M	N	K	F	A	N	Q	N	M	I	R	NYD	1	ANCSI	1
RF7	S.E. Asia	No	C	**I**	**E**	**T**	F	**S**	N	**E**	**S**	**L**	**T**	**I**	N**F**D	1	A**IRNL**	3
7G8	S. America	No	**S**	M	N	**T**	F	**S**	N	Q	**D**	M	**L**	R	N**FY**	1	A**I**C**N**I	1
7G8$^{\text{7G8crt+F145I}}$		Yes	**S**	M	N	**T**	**I**	**S**	N	Q	**D**	M	**L**	R	N**FY**	1	A**I**C**N**I	1
Cam3.II	S.E. Asia	No	C	**I**	**E**	**T**	F	**S**	N	**E**	**S**	M	**T**	**I**	N**F**D	2	A**IRN**I	1
NF10	S.E. Asia	No	C	**I**	**E**	**T**	F	**S**	N	**E**	**S**	M	**T**	**I**	**YF**D	1	A**I**C**N**I	1

Mutant residues are highlighted in bold and shaded in gray.

Supplementary Table 2: *In vitro* activity of ZY19489 against PfV1-D cKD lines. These lines express normal or reduced levels of subunit D of the *P. falciparum* vacuolar proton-transporting V-type ATPase complex (referred to as V1-D) upon culturing with 50 nM, 6 nM or 0 nM aTc.

Compound	cKD line	Mean IC_{50} ± SEM		IC_{50} fold shift	Comment
		(with aTc)	(low/no aTc)		
ZY19489	V1-D	7.2 ± 0.2	7.7 ± 0.1	No shift	No interaction
	control	7.1 ± 1.3	6.7 ± 0.6	No shift	
Bafilomycin A	V1-D	4.1 ± 0.1	1.7 ± 0.1	2.4×	Hypersensitive

Experiments were conducted on 2 independent occasions with technical duplicates (*N*, n = *2*, 2).

Supplementary Table 3: ***In vitro* 72-hour susceptibility of drug-selected bulk parasites and clones tested against ZY19489.**

**Parasite Line**	**ZY19489**	
	**IC_{50} (nM)**	**IC_{90} (nM)**
Dd2-Polδ (parent)	8.2 ± 0.5	18.4 ± 3.2
Dd2-Polδ Flask 2 (bulk)	96.4 ± 5.8**	201.1 ± 12.4**
Flask 2 clone C8 (FL2-C8)	110.6 ± 6.3**	236.3 ± 9.9**
Flask 2 clone E3 (FL2-E3)	107.8 ± 4.4**	213.6 ± 12.1**
Flask 2 clone F7 (FL2-F7)	106.8 ± 3.7**	220.4 ± 9.7**
Flask 2 clone G2 (FL2-G2)	104.8 ± 3.4**	207.8 ± 13.1**

Data show mean ± SEM IC_{50} values, calculated from 5 to 6 independent experiments with technical duplicates. Values for the bulk resistant culture and the subsequent clones were compared against the Dd2-Polδ parent, with statistical significance determined using two-tailed Mann-Whitney *U* tests. ***p* = 0.0043.

Supplementary Table 4: **Genes located within the ~80kb amplified region of chromosome 5 in clone C8.**

Gene ID	PlasmoDB annotation	FL2_C8 factor
PF3D7_0521900	conserved *Plasmodium* protein, unknown function	1.53
PF3D7_0522000	conserved *Plasmodium* protein, unknown function	1.53
PF3D7_0522100	conserved *Plasmodium* protein, unknown function	1.53
PF3D7_0522200	transcription initiation factor TFIID subunit 10, putative	1.53
PF3D7_0522300	18S rRNA (guanine-N(7))-methyltransferase, putative	1.53
PF3D7_0522400	conserved Plasmodium protein, unknown function	1.53
PF3D7_0522500	50S ribosomal protein L17, apicoplast, putative	1.53
PF3D7_0522600	magnesium transporter NIPA, putative	1.53
PF3D7_0522700	iron-sulfur cluster assembly protein SufA	1.53
PF3D7_0522800	pre-mRNA-splicing factor BUD31, putative	1.53
PF3D7_0522900	zinc finger protein, putative	1.53
**PF3D7_0523000**	**multidrug resistance protein 1**	**1.53**
PF3D7_0523100	mitochondrial-processing peptidase subunit alpha, putative	1.53
PF3D7_0523200	heptatricopeptide repeat-containing protein, putative	1.53
PF3D7_0523300	cytochrome c oxidase subunit ApiCOX18, putative	1.53

This chromosome 5 segment is duplicated in Dd2-Polδ compared with the reference strain 3D7. The 1.53-fold amplification denoted herein is consistent with this region now being present as three tandem copies, compared with the presence of two tandem copies in parental Dd2-Polδ.

Supplementary Table 5: ***In vitro*** **72-hour susceptibility of edited parasites clones against ZY19489.**

Parasite line/clone	ZY19489	
	IC_{50} (nM)	IC_{90} (nM)
$Dd2^{Dd2crt}$	8.5 ± 0.4	16.9 ± 4.4
$Dd2^{Dd2crt+N246H}$ clone A7	90.9 ± 4.1**	178.1 ± 11.2**
$Dd2^{Dd2crt+N246H}$ clone B9	89.1 ± 5.0**	189.3 ± 9.3**
$Dd2^{GB4crt}$	8.6 ± 0.7	18.4 ± 2.1
$Dd2^{GB4crt+N246H}$	68.9 ± 7.1**	138.7 ± 9.4**
$Dd2^{FCBcrt}$	18.5 ± 1.6	39.7 ± 6.7
$Dd2^{FCBcrt+N246H}$	90.3 ± 8.8*	179.7 ± 22.2*
$Dd2^{Dd2apc10+D233N}$ clone C3	7.7 ± 0.7	17.6 ± 3.6
$Dd2^{Dd2apc10+D233N}$ clone G7	8.8 ± 1.0	19.1 ± 1.4

Data show means ± SEM IC_{50} values, calculated from 4 to 6 independent experiments with technical duplicates. Significance was determined using two-tailed Mann-Whitney *U* tests; *$p<0.05$ and **$p<0.01$.

Supplementary Table 6: ***In vitro*** **72-hour potency of APC10 and ubiquitin ligase inhibitors and chloroquine as a control inhibitor against $Dd2^{Dd2}$ and $Dd2^{Dd2crt+apc10\ D233N}$ parasites.**

Parasite line	Nutlin-3		Thalidomide		pro-TAME		Chloroquine	
	IC_{50} (nM)	IC_{90} (nM)	IC_{50} (nM)	IC_{90} (nM)	IC_{50} (nM)	IC_{90} (nM)	IC_{50} (nM)	IC_{90} (nM)
$Dd2^{Dd2crt}$	5656 ± 511	9129 ± 714	85218 ± 5061	115735 ± 1111	58427 ± 7096	91019 ± 11341	94.6 ± 6.6	177.3 ± 24.1
$Dd2^{Dd2crt+apc10\ D233N}$	5396 ± 451	8519 ± 866	88416 ± 3271	118591 ± 1263	55693 ± 2791	94629 ± 8497	84.9 ± 8.4	171.8 ± 31.3

Data show means ± SEM, calculated from 4 to 5 independent experiments with technical duplicates. Statistical significance was tested using two-tailed Mann-Whitney *U* tests.

Supplementary Table 7: *In vitro* 72-hour susceptibility of FCB and FCB-KD^{mdr1} parasites against ZY19489, mefloquine (MFQ) and lumefantrine (LMF).

	ZY19489		MFQ		LMF	
**Parasite line/clone**	**IC_{50} (nM)**	**IC_{90} (nM)**	**IC_{50} (nM**	**IC_{90} (nM)**	**IC_{50} (nM)**	**IC_{90} (nM)**
FCB	8.7 ± 0.6	15.7 ± 0.6	10.0 ± 0.6	23.9 ± 3.4	1.9 ± 0.2	3.1 ± 0.4
FCB-KD^{mdr1}	8.5 ± 0.8	17.4 ± 2.8	5.1 ± 0.4**	10.7 ± 1.1**	1.0 ± 0.1*	1.9 ± 0.1*

Data show means ± SEM IC_{50} values, calculated from 4 to 6 independent experiments with technical duplicates. Significance was determined using two-tailed Mann-Whitney *U* tests; *p = 0.0286, **p = 0.0079.

FCB has two 100-kb amplicons that each contain 1 copy of *pfmdr1* whereas FCB-KD^{mdr1} expresses only one due to targeted disruption of the second copy.

Supplementary Table 8 (p1). List of 34 genes in the chromosome 7 QTL peak harboring non-synonymous mutations between RF7 and NF54

Gene	AA_change	Codon change	Description	pos	ref	alt
PF3D7_0703900	T3212N	aCt/aAt	conserved Plasmodium membrane protein, unknown function	156762	G	T
PF3D7_0703900	V3173I	Gtt/Att	conserved Plasmodium membrane protein, unknown function	156880	C	T
PF3D7_0703900	S2890N	aGt/aAt	conserved Plasmodium membrane protein, unknown function	157728	C	T
PF3D7_0703900	A2175S	Gct/Tct	conserved Plasmodium membrane protein, unknown function	159874	C	A
PF3D7_0703900	R2174S	agA/agT	conserved Plasmodium membrane protein, unknown function	159875	T	A
PF3D7_0703900	N2157H	Aat/Cat	conserved Plasmodium membrane protein, unknown function	159928	T	G
PF3D7_0703900	H2102N	Cat/Aat	conserved Plasmodium membrane protein, unknown function	160093	G	T
PF3D7_0703900	L1955F	ttA/ttC	conserved Plasmodium membrane protein, unknown function	160532	T	G
PF3D7_0703900	M1880V	Atg/Gtg	conserved Plasmodium membrane protein, unknown function	160759	T	C
PF3D7_0703900	Q1410H	caA/caT	conserved Plasmodium membrane protein, unknown function	162167	T	A
PF3D7_0703900	T848I	aCt/aTt	conserved Plasmodium membrane protein, unknown function	163854	G	A
PF3D7_0703900	N377K	aaC/aaA	conserved Plasmodium membrane protein, unknown function	165266	G	T
PF3D7_0703900	E248K	Gaa/Aaa	conserved Plasmodium membrane protein, unknown function	165655	C	T
PF3D7_0704000	K2678E	Aag/Gag	conserved Plasmodium membrane protein, unknown function	169264	T	C
PF3D7_0704000	I2662M	atA/atG	conserved Plasmodium membrane protein, unknown function	169310	T	C
PF3D7_0704000	Q2464K	Caa/Aaa	conserved Plasmodium membrane protein, unknown function	169906	G	T
PF3D7_0704000	S1975C	Agt/Tgt	conserved Plasmodium membrane protein, unknown function	171373	T	A
PF3D7_0704000	M1744I	atG/atC	conserved Plasmodium membrane protein, unknown function	172064	C	G
PF3D7_0704000	K1661N	aaA/aaT	conserved Plasmodium membrane protein, unknown function	172313	T	A
PF3D7_0704000	G422D	gGt/gAt	conserved Plasmodium membrane protein, unknown function	176031	C	T
PF3D7_0704100	L66I	Cta/Ata	basal complex transmembrane protein 2	180231	C	A
PF3D7_0704100	E152K	Gaa/Aaa	basal complex transmembrane protein 2	180489	G	A
PF3D7_0704100	G804V	gGt/gTt	basal complex transmembrane protein 2	182446	G	T
PF3D7_0704100	S1084C	Agt/Tgt	basal complex transmembrane protein 2	183285	A	T
PF3D7_0704100	I1373N	aTt/aAt	basal complex transmembrane protein 2	184153	T	A
PF3D7_0704100	N1377I	aAt/aTt	basal complex transmembrane protein 2	184165	A	T
PF3D7_0704100	K2486N	aaG/aaT	basal complex transmembrane protein 2	187493	G	T
PF3D7_0704200	D1069H	Gat/Cat	tRNA m5C-methyltransferase, putative	191686	C	G
PF3D7_0704200	Y950C	tAt/tGt	tRNA m5C-methyltransferase, putative	192042	T	C
PF3D7_0704200	S713N	aGt/aAt	tRNA m5C-methyltransferase, putative	192753	C	T
PF3D7_0704200	L700I	Tta/Ata	tRNA m5C-methyltransferase, putative	192793	A	T
PF3D7_0704300	I1482K	aTa/aAa	conserved Plasmodium membrane protein, unknown function	202135	T	A
PF3D7_0704300	I1487K	aTa/aAa	conserved Plasmodium membrane protein, unknown function	202150	T	A
PF3D7_0704300	I1512K	aTa/aAa	conserved Plasmodium membrane protein, unknown function	202225	T	A
PF3D7_0704300	K1527E	Aaa/Gaa	conserved Plasmodium membrane protein, unknown function	202269	A	G
PF3D7_0704300	G1530D	gGt/gAt	conserved Plasmodium membrane protein, unknown function	202279	G	A
PF3D7_0704400	K454N	aaG/aaT	phosphoinositide-binding protein, putative	206079	G	T
PF3D7_0704500	G799V	gGa/gTa	serine/threonine protein kinase, putative	211106	C	A
PF3D7_0704500	D784H	Gat/Cat	serine/threonine protein kinase, putative	211152	C	G
PF3D7_0704500	S500R	agT/agA	serine/threonine protein kinase, putative	212002	A	T
PF3D7_0704600	H3608P	cAt/cCt	HECT-type E3 ubiquitin ligase UT	218066	T	G
PF3D7_0704600	N2311K	aaT/aaG	HECT-type E3 ubiquitin ligase UT	221956	A	C
PF3D7_0704600	N2125D	Aat/Gat	HECT-type E3 ubiquitin ligase UT	222516	T	C
PF3D7_0704600	P1667H	cCt/cAt	HECT-type E3 ubiquitin ligase UT	223889	G	T
PF3D7_0704600	K1587E	Aaa/Gaa	HECT-type E3 ubiquitin ligase UT	224130	T	C
PF3D7_0704600	E1585K	Gaa/Aaa	HECT-type E3 ubiquitin ligase UT	224136	C	T
PF3D7_0704600	D1419N	Gat/Aat	HECT-type E3 ubiquitin ligase UT	224634	C	T
PF3D7_0704600	Y1387F	tAt/tTt	HECT-type E3 ubiquitin ligase UT	224729	T	A
PF3D7_0704600	P1384A	Cct/Gct	HECT-type E3 ubiquitin ligase UT	224739	G	C
PF3D7_0704600	N1375S	aAt/aGt	HECT-type E3 ubiquitin ligase UT	224765	T	C
PF3D7_0704600	Y1232C	tAt/tGt	HECT-type E3 ubiquitin ligase UT	225194	T	C
PF3D7_0704600	R735K	aGg/aAg	HECT-type E3 ubiquitin ligase UT	226685	C	T
PF3D7_0704600	P535L	cCa/cTa	HECT-type E3 ubiquitin ligase UT	227285	G	A
PF3D7_0704600	C490S	Tgt/Agt	HECT-type E3 ubiquitin ligase UT	227421	A	T
PF3D7_0704700	A804V	gCt/gTt	phosphopantetheine adenylyltransferase, putative	235811	C	T
PF3D7_0704700	R1037S	agG/agT	phosphopantetheine adenylyltransferase, putative	236511	G	T
PF3D7_0704800	E1059Q	Gaa/Caa	protein phosphatase PPM12, putative	238878	C	G
PF3D7_0704800	N422D	Aac/Gac	protein phosphatase PPM12, putative	240789	T	C
PF3D7_0704800	M421L	Atg/Ttg	protein phosphatase PPM12, putative	240792	T	A
PF3D7_0704800	A419S	Gca/Tca	protein phosphatase PPM12, putative	240798	C	A
PF3D7_0704800	D417N	Gac/Aac	protein phosphatase PPM12, putative	240804	C	T
PF3D7_0704800	L416M	Ttg/Atg	protein phosphatase PPM12, putative	240807	A	T

Supplementary Table 8 (p2). List of 34 genes in the chromosome 7 QTL peak harboring non-synonymous mutations between RF7 and NF54

Gene	AA_change	Codon change	Description	pos	ref	alt
PF3D7_0704800	H415N	Cat/Aat	protein phosphatase PPM12, putative	240810	G	T
PF3D7_0704800	A414E	gCa/gAa	protein phosphatase PPM12, putative	240812	G	T
PF3D7_0704800	D412N	Gac/Aac	protein phosphatase PPM12, putative	240819	C	T
PF3D7_0705000	I260N	aTt/aAt	mRNA cap guanine-N7 methyltransferase, putative	249374	A	T
PF3D7_0705100	I29M	atC/atG	conserved Plasmodium protein, unknown function	252368	C	G
PF3D7_0705100	R564S	agG/agT	conserved Plasmodium protein, unknown function	253973	G	T
PF3D7_0705100	N581K	aaT/aaA	conserved Plasmodium protein, unknown function	254024	T	A
PF3D7_0705100	N585D	Aac/Gac	conserved Plasmodium protein, unknown function	254034	A	G
PF3D7_0705100	G722D	gGc/gAc	conserved Plasmodium protein, unknown function	254446	G	A
PF3D7_0705200	I594V	Ata/Gta	conserved Plasmodium protein, unknown function	257865	A	G
PF3D7_0705200	K643Q	Aaa/Caa	conserved Plasmodium protein, unknown function	258012	A	C
PF3D7_0705200	T717A	Aca/Gca	conserved Plasmodium protein, unknown function	258234	A	G
PF3D7_0705300	A101T	Gcc/Acc	origin recognition complex subunit 2	262277	G	A
PF3D7_0705500	G2736D	gGc/gAc	inositol-phosphate phosphatase, putative	270260	C	T
PF3D7_0705500	H2584Y	Cat/Tat	inositol-phosphate phosphatase, putative	270957	G	A
PF3D7_0705500	Y1206D	Tat/Gat	inositol-phosphate phosphatase, putative	275091	A	C
PF3D7_0705500	M443I	atG/atA	inositol-phosphate phosphatase, putative	277378	C	T
PF3D7_0706000	K1135N	aaA/aaT	importin-7, putative	296804	A	T
PF3D7_0706100	S1334R	Agc/Cgc	conserved Plasmodium protein, unknown function	298651	T	G
PF3D7_0706100	N934Y	Aat/Tat	conserved Plasmodium protein, unknown function	299971	T	A
PF3D7_0706500	N1364K	aaT/aaG	conserved Plasmodium protein, unknown function	310931	A	C
PF3D7_0706500	Y1358N	Tat/Aat	conserved Plasmodium protein, unknown function	310951	A	T
PF3D7_0706500	N436S	aAt/aGt	conserved Plasmodium protein, unknown function	313716	T	C
PF3D7_0706500	S406N	aGt/aAt	conserved Plasmodium protein, unknown function	313806	C	T
PF3D7_0706600	N81H	Aat/Cat	conserved Plasmodium protein, unknown function	320488	A	C
PF3D7_0707200	I1025N	aTt/aAt	conserved Plasmodium protein, unknown function	333252	A	T
PF3D7_0707200	I1007N	aTt/aAt	conserved Plasmodium protein, unknown function	333306	A	T
PF3D7_0707200	D985G	gAt/gGt	conserved Plasmodium protein, unknown function	333372	T	C
PF3D7_0707200	H983D	Cat/Gat	conserved Plasmodium protein, unknown function	333379	G	C
PF3D7_0707200	C982S	Tgt/Agt	conserved Plasmodium protein, unknown function	333382	A	T
PF3D7_0707200	F973L	ttT/ttA	conserved Plasmodium protein, unknown function	333407	A	T
PF3D7_0707200	I942M	atT/atG	conserved Plasmodium protein, unknown function	333500	A	C
PF3D7_0707500	I979M	atT/atG	conserved Plasmodium protein, unknown function	351213	T	G
PF3D7_0707500	V1420I	Gta/Ata	conserved Plasmodium protein, unknown function	352534	G	A
PF3D7_0707700	R237K	aGa/aAa	E3 ubiquitin-protein ligase, putative	358409	G	A
PF3D7_0707700	S577G	Agt/Ggt	E3 ubiquitin-protein ligase, putative	359428	A	G
PF3D7_0708200	E795D	gaA/gaT	conserved Plasmodium protein, unknown function	374723	T	A
PF3D7_0708200	S526G	Agt/Ggt	conserved Plasmodium protein, unknown function	375818	T	C
PF3D7_0708200	R289K	aGa/aAa	conserved Plasmodium protein, unknown function	376528	C	T
PF3D7_0708500	D801N	Gat/Aat	heat shock protein 86 family protein	385921	C	T
PF3D7_0708500	E466K	Gaa/Aaa	heat shock protein 86 family protein	386926	C	T
PF3D7_0708500	K154N	aaG/aaT	heat shock protein 86 family protein	387860	C	A
PF3D7_0708700	D200E	gaT/gaA	Cg8 protein	391589	A	T
PF3D7_0709000	M74I	atG/atT	chloroquine resistance transporter	403620	G	T
PF3D7_0709000	N75D	Aat/Gat	chloroquine resistance transporter	403621	A	G
PF3D7_0709000	N75K	aaT/aaA	chloroquine resistance transporter	403623	T	A
PF3D7_0709000	K76T	aAa/aCa	chloroquine resistance transporter	403625	A	C
PF3D7_0709000	A220S	Gcc/Tcc	chloroquine resistance transporter	404407	G	T
PF3D7_0709000	Q271E	Caa/Gaa	chloroquine resistance transporter	404836	C	G
PF3D7_0709000	N326S	aAc/aGc	chloroquine resistance transporter	405362	A	G
PF3D7_0709000	M343L	Atg/Ttg	chloroquine resistance transporter	405560	A	T
PF3D7_0709000	I356T	aTa/aCa	chloroquine resistance transporter	405600	T	C
PF3D7_0709000	R371I	aGa/aTa	chloroquine resistance transporter	405838	G	T
PF3D7_0709100	E161D	gaA/gaC	Cg1 protein	408697	A	C
PF3D7_0709100	V177I	Gtc/Atc	Cg1 protein	408743	G	A
PF3D7_0709100	N608D	Aat/Gat	Cg1 protein	410036	A	G
PF3D7_0709100	P676S	Cca/Tca	Cg1 protein	410240	C	T
PF3D7_0709100	T732R	aCg/aGg	Cg1 protein	410409	C	G
PF3D7_0709100	S740P	Tca/Cca	Cg1 protein	410432	T	C
PF3D7_0709100	M828I	atG/atA	Cg1 protein	410698	G	A
PF3D7_0709100	K1121N	aaA/aaC	Cg1 protein	411577	A	C
PF3D7_0709100	N1122D	Aac/Gac	Cg1 protein	411578	A	G

Supplementary Table 8 (p3). List of 34 genes in the chromosome 7 QTL peak harboring non-synonymous mutations between RF7 and NF54

Gene	AA change	Codon change	Description	pos	ref	alt
PF3D7_0709100	E1128V	gAa/gTa	Cg1 protein	411597	A	T
PF3D7_0709100	E1128D	gaA/gaT	Cg1 protein	411598	A	T
PF3D7_0709200	L124V	Ctt/Gtt	glutaredoxin-like protein	412792	G	C
PF3D7_0709200	G114E	gGg/gAg	glutaredoxin-like protein	412821	C	T
PF3D7_0709300	S2483N	aGt/aAt	Cg2 protein	414302	C	T
PF3D7_0709300	L2379M	Ttg/Atg	Cg2 protein	414615	A	T
PF3D7_0709300	G2334C	Ggt/Tgt	Cg2 protein	414750	C	A
PF3D7_0709300	M2325I	atG/atA	Cg2 protein	414775	C	T
PF3D7_0709300	M2325K	aTg/aAg	Cg2 protein	414776	A	T
PF3D7_0709300	K2324N	aaA/aaT	Cg2 protein	414778	T	A
PF3D7_0709300	M2319I	atG/atA	Cg2 protein	414793	C	T
PF3D7_0709300	M2313I	atG/atA	Cg2 protein	414811	C	T
PF3D7_0709300	D2205N	Gat/Aat	Cg2 protein	415137	C	T
PF3D7_0709300	H2169Y	Cat/Tat	Cg2 protein	415245	G	A
PF3D7_0709300	N2167S	aAt/aGt	Cg2 protein	415250	T	C
PF3D7_0709300	H2074P	cAt/cCt	Cg2 protein	415529	T	G
PF3D7_0709300	Q2069H	caA/caC	Cg2 protein	415543	T	G
PF3D7_0709300	V2042G	gTt/gGt	Cg2 protein	415625	A	C
PF3D7_0709300	S1992N	aGt/aAt	Cg2 protein	415775	C	T
PF3D7_0709300	L1883V	Cta/Gta	Cg2 protein	416103	G	C
PF3D7_0709300	S1426C	Agt/Tgt	Cg2 protein	417474	T	A
PF3D7_0709300	M1350I	atG/atA	Cg2 protein	417700	C	T
PF3D7_0709300	F989L	ttC/ttA	Cg2 protein	418783	G	T
PF3D7_0709300	Y814N	Tat/Aat	Cg2 protein	419310	A	T
PF3D7_0709300	Y716F	tAt/tTt	Cg2 protein	419603	T	A
PF3D7_0709300	G281A	gGa/gCa	Cg2 protein	420908	C	G
PF3D7_0709300	H275Q	caC/caG	Cg2 protein	420925	G	C
PF3D7_0709400	Y172H	Tat/Cat	Cg7 protein	426721	A	G
PF3D7_0709400	V161E	gTg/gAg	Cg7 protein	426753	A	T
PF3D7_0709600	K325N	aaA/aaT	ribonucleases P/MRP protein subunit POP1, putative	432784	T	A
PF3D7_0709600	D80E	gaT/gaA	ribonucleases P/MRP protein subunit POP1, putative	433519	A	T
PF3D7_0709700	M261I	atG/atA	prodrug activation and resistance esterase	435413	C	T
PF3D7_0709900	T368S	Act/Tct	conserved protein, unknown function	444780	T	A
PF3D7_0709900	T362I	aCa/aTa	conserved protein, unknown function	444797	G	A
PF3D7_0709900	H338Y	Cat/Tat	conserved protein, unknown function	444870	G	A
PF3D7_0709900	S336C	tCc/tGc	conserved protein, unknown function	444875	G	C
PF3D7_0709900	V267G	gTa/gGa	conserved protein, unknown function	445082	A	C
PF3D7_0710000	D3098E	gaC/gaG	conserved Plasmodium protein, unknown function	448025	G	C
PF3D7_0710000	D3077E	gaC/gaG	conserved Plasmodium protein, unknown function	448088	G	C
PF3D7_0710000	T2583I	aCa/aTa	conserved Plasmodium protein, unknown function	449571	G	A
PF3D7_0710000	L2544F	ttA/ttT	conserved Plasmodium protein, unknown function	449687	T	A
PF3D7_0710000	I2543L	Ata/Tta	conserved Plasmodium protein, unknown function	449692	T	A
PF3D7_0710000	D2510V	gAt/gTt	conserved Plasmodium protein, unknown function	449790	T	A
PF3D7_0710000	Y2435H	Tat/Cat	conserved Plasmodium protein, unknown function	450016	A	G
PF3D7_0710000	H2299N	Cat/Aat	conserved Plasmodium protein, unknown function	450424	G	T
PF3D7_0710000	S2065G	Agt/Ggt	conserved Plasmodium protein, unknown function	451126	T	C
PF3D7_0710000	I1962L	Ata/Tta	conserved Plasmodium protein, unknown function	451435	T	A
PF3D7_0710000	K1893N	aaA/aaC	conserved Plasmodium protein, unknown function	451640	T	G
PF3D7_0710000	D1881G	gAc/gGc	conserved Plasmodium protein, unknown function	451677	T	C
PF3D7_0710000	Y1782N	Tat/Aat	conserved Plasmodium protein, unknown function	451975	A	T
PF3D7_0710000	S1663L	tCg/tTg	conserved Plasmodium protein, unknown function	452331	G	A
PF3D7_0710000	T1658K	aCa/aAa	conserved Plasmodium protein, unknown function	452346	G	T
PF3D7_0710000	E1519Q	Gag/Cag	conserved Plasmodium protein, unknown function	452764	C	G
PF3D7_0710000	I909L	Ata/Tta	conserved Plasmodium protein, unknown function	455550	T	A
PF3D7_0710000	M883I	atG/atT	conserved Plasmodium protein, unknown function	455626	C	A
PF3D7_0710000	M755I	atG/atC	conserved Plasmodium protein, unknown function	456232	C	G
PF3D7_0710000	Q655H	caA/caC	conserved Plasmodium protein, unknown function	456532	T	G

The following progeny harbor the **RF7 parental** sequence: HapN (msX1_msX1LR_RF7_HapN); HapG (msX1_msX1LR_46056_25_C3_HapG); HapI (msX1_nxX1_46056_4_F2_A8_HapI); HapO (msX1_nxX1_46056_0618_mdCQ_B7_HapO); HapQ (msX1_nxX1_46056_1117_PPQ_1F2_HapQ); HapR (msX1_nxX1_46056_1117_PPQ_1F3_HapR); HapS (msX1_nxX1_46056_1117_PPQ_1F9_HapS2); HapU (msX1_nxX1_46056_1117_PPQ_2E9_HapU); HapV (msX1_nxX1_46056_1117_PPQ_2G8_HapV); HapW (msX1_nxX1_46056_1117_PYM_A4_HapW); HapX (msX1_nxX1_46056_1117_PYM_B4_HapX); HapZ (msX1_nxX1_46056_1117_PYM_C3_HapZ); Hap8 (msX1_nxX1_46056_1117_mdCQ_1G6_Hap8); Hap9 (msX1_nxX1_46056_1117_mdCQ_2A6_Hap9); Hap7 (msX1_nxX1_46056_1117_mdCQ_2E3_Hap7); Hap10 (msX1_nxX1_46056_1117_mdCQ_3D2_Hap10).

The following progeny harbor the **NF54 parental** sequence: HapA (msX1_msX1LR_NF54_HapA); HapH (msX1_msX1LR_45915_5_F5_HapH); HapF (msX1_msX1LR_46033_3_D4_HapF); HapJ (msX1_nsX1LR_10B3_HapJ); HapL (msX1_nsX1LR_7G4_HapL); HapB (msX1_nxX1_46033_1_E6_HapB); HapE (msX1_nxX1_46033_5_D9_G7_HapE); HapK (msX1_nxX1_46033_mdCQ_5_HapK); HapD ()msX1_nxX1_46056_8_D11_G3_HapD); HapM (msX1_nxX1_46056_9_E12_E5_HapM); HapC (msX1_nxX1_46056_DHAx2_4_6_HapC); HapP (msX1_nxX1_46056_0618_mdCQ_G8_HapP); HapT (msX1_nxX1_46056_1117_PPQ_1D8_HapT); HapY (msX1_nxX1_46056_1117_PYM_B6_HapY); Hap1 (msX1_nxX1_46056_1117_PYM_C6_Hap1); Hap2 (msX1_nxX1_46056_1117_PYM_E3_Hap2); Hap3 (msX1_nxX1_46056_1117_PYM_F3_Hap3); Hap4 (msX1_nxX1_46056_1117_PYM_F4_Hap4); Hap5 (msX1_nxX1_46056_1117_PYM_G11_Hap5); Hap6 (msX1_nxX1_46056_1117_PYM_G7_Hap6).

Supplementary Table 9: Chemical Pairwise Linear Potential (ChemPLP) docking scores for ZY19489 and solvent-accessible surface area (SASA) values. These were calculated for representative structures of parental (7G8) and mutant (7G8+N246H) proteins in the open-to-DV and open-to-cytosol conformational states in complex with ZY19489.

	**7G8 parental**	**7G8+N246H mutant *pfcrt***
ChemPLP Open-to-DV	40.1	54.5
ChemPLP Open-to-cytosol	58.3	50.4
SASA Open-to-DV (Å²)	19851	20369
SASA Open-to-cytosol (Å²)	23418	22519

Supplementary Table 10: *In vitro* 72-hour susceptibility of $Dd2^{Dd2crt}$, $Dd2^{Dd2crt+N246H}$, $Dd2^{GB4crt}$ and $Dd2^{GB4crt+N246H}$ against 7 clinically important antimalarials. These are ferroquine (FQ), chloroquine (CQ), piperaquine (PPQ), mono-desethylamodiaquine (DEAQ), lumefantrine (LMF), atovaquone (ATQ) and dihydroartemisinin (DHA).

Parasite line	FQ IC_{50}	FQ IC_{90}	CQ IC_{50}	CQ IC_{90}	PPQ IC_{50}	PPQ IC_{90}	DEAQ IC_{50}	DEAQ IC_{90}	LMF IC_{50}	LMF IC_{90}	ATQ IC_{50}	ATQ IC_{90}	DHA IC_{50}	DHA IC_{90}
$Dd2^{Dd2crt}$	18.4 ± 1.2	39.1 ± 2.3	103.5 ± 5.7	217.4 ± 11.3	12.0 ± 1.0	22.3 ± 4.6	43.8 ± 5.9	78.7 ± 8.8	6.0 ± 1.2	11.2 ± 1.8	2.5 ± 0.4	5.3 ± 0.7	2.6 ± 0.2	4.7 ± 0.6
$Dd2^{Dd2crt+N246H}$	6.6 ± 0.8*	17.2 ± 1.4	10.9 ± 2.1*	23.8 ± 1.7	3.7 ± 0.5*	9.3 ± 1.4	7.7 ± 0.5**	16.2 ± 1.7	4.9 ± 0.7	9.3 ± 1.2	2.6 ± 0.2	4.4 ± 0.4	3.0 ± 0.2	6.2 ± 0.5
$Dd2^{GB4crt}$	21.1 ± 2.1	37.8 ± 4.4	79.8 ± 5.6	137.4 ± 9.8	9.4 ± 1.2	16.3 ± 2.9	36.9 ± 4.0	67.9 ± 9.4	3.4 ± 0.5	7.2 ± 1.2	1.9 ± 0.3	4.1 ± 0.6	2.2 ± 0.2	3.9 ± 0.3
$Dd2^{GB4crt+N246H}$	7.3 ± 0.8*	13.9 ± 2.7	7.2 ± 0.7*	15.3 ± 1.4	2.9 ± 0.4*	4.7 ± 0.8	5.6 ± 0.7**	11.82 ± 1.3	2.8 ± 0.4	5.3 ± 0.9	2.3 ± 0.3	3.9 ± 0.8	1.7 ± 0.3	3.5 ± 0.6

Data show means ± SEM IC_{50} values, calculated from 4 to 6 independent experiments with technical duplicates. Significance was determined using two-tailed Mann- Whitney U tests; *p = 0.0286, **p = 0.0079.

Supplementary Table 11: *In vitro* 72-hour susceptibility of edited and drug-selected PfCRT and PfMDR1 mutants tested against ZY19489. The mutations studied herein represent some of the currently circulating alleles in the field.

	ZY19489	
**Parasite line/clone**	**IC_{50} (nM)**	**IC_{90} (nM)**
$Dd2^{Dd2crt}$	10.2 ± 0.8	19.8 ± 1.1
$Dd2^{3D7crt}$	7.1 ± 0.8	15.8 ± 1.2
$Dd2^{Dd2crt+T93S}$	9.5 ± 0.7	20.5 ± 1.5
$Dd2^{Dd2crt+H97Y}$	9.4 ± 1.0	21.7 ± 1.6
$Dd2^{Dd2crt+F145I}$	11.5 ± 1.1	22.3 ± 2.3
$Dd2^{Dd2crt+I218F}$	8.3 ± 0.6	18.5 ± 2.1
$Dd2^{Dd2crt+M343L}$	9.4 ± 0.7	21.4 ± 1.7
$Dd2^{Dd2crt+G353V}$	13.7 ± 0.9	20.7 ± 1.7
$7G8^{7G8crt}$	13.6 ± 0.7	18.6 ± 1.4
$7G8^{7G8crt+C350R}$	14.0 ± 0.7	17.9 ± 1.3
Cam3.II C580Y	15.9 ± 1.3	34.4 ± 1.4
Cam3.II $C580Y^{S784Lmdr1}$	17.0 ± 1.4	35.9 ± 3.2
Dd2 (B2 clone)	10.2 ± 0.8	22.8 ± 1.7
$Dd2^{F1072Lmdr1}$	31.3 ± 1.5**	49.4 ± 2.3
$Dd2^{S1075Imdr1}$	29.6 ± 1.7**	48.0 ± 2.7
NF10	19.0 ± 1.3	39.5 ± 2.4
$NF10^{A750Tmdr1}$	18.4 ± 1.8	39.9 ± 2.7
NF54	8.3 ± 0.6	21.2 ± 1.1
$NF54^{M841I+M924Imdr1}$	9.9 ± 0.7	22.0 ± 1.8

Data show means ± SEM, calculated from 4 to 6 independent experiments with technical duplicates. Significance was determined using two-tailed Mann-Whitney *U* tests where $^{**}p = 0.0079$.

Supplementary Table 12: 72-hour *in vitro* activity of ZY19489 and M5717 against M5717-resistant *Pf*eEF2 mutant parasites recovered from humanized NSG mice.

Parasite line	M5717 treatment	N	*Pf*eEF2 genotype (% mutant)	IC_{50} ± SEM (nM)	IC_{90} ± SEM (nM)	IC_{50} FC	IC_{90} FC
				**ZY19489**			
**TAD_022**	-	3	Wildtype	7.2 ± 0.9	17.1 ± 1.1	1.0	1.0
**TAD_470**	1 × 6 mg/kg	3	I183M (80%)	8.7 ± 1.1	18.2 ± 1.4	1.2	1.1
**TAD_462**	1 × 12 mg/kg	3	P754S (100%)	8.5 ± 0.7	18.7 ± 0.9	1.2	1.1
**TAD_464**	1 × 40 mg/kg	3	I182T (100%)	7.9 ± 0.4	17.5 ± 1.3	1.1	1.0
				**M5717**			
**TAD_022**	-	4	Wildtype	0.4 ± 0.01	0.8 ± 0.1	1.0	1.0
**TAD_470**	1 × 6 mg/kg	4	I183M (80%)	1.7 ± 0.2	6.4 ± 0.3	4.5***	8.4****
**TAD_462**	1 × 12 mg/kg	4	P754S (100%)	56.4 ± 2.3	198.9 ± 8.2	150.5****	261.7****
**TAD_464**	1 × 40 mg/kg	4	I182T (100%)	97.3 ± 7.8	321.5 ± 19.1	259.4****	423.0****

IC_{50} and IC_{90} values were measured against the untreated, drug-sensitive *P. falciparum* $3D7^{0087/N9}$ parental line (TAD_022) and recrudescent lines (TAD_470, TAD_462, TAD_464) from $3D7^{0087/N9}$-infected mice treated with a single M5717 dose of 6, 12, or 40 mg/kg, respectively. *PfeEF2* genotypes and estimated variant allele frequencies (shown in parentheses) were determined by targeted amplicon sequencing of the *PfeEF2* (PF3D7_1451100) locus. Fold-changes (FC) in IC_{50} and IC_{90} values for each recrudescent line relative to the parental strain are also reported IC_{50} and IC_{90} values represent means ± SEM from 3 (ZY19489) to 4 (M5717) independent experiments (N), each performed in duplicate. Statistical significance was assessed relative to the TAD_022 control using two-tailed unpaired Students t-tests (GraphPad Prism 9) where ***$p < 0.001$ and ****$p < 0.0001$.

Supplementary Table 13: Stage-specific activity against 3D7 parasites of ZY19489 and control antimalarial compounds, chloroquine, MMV390048 and dihydroartemisinin after 12-hour incubation intervals and after 72 hours.

Compound	Rings (0-12hrs)		Trophozoites (16-30hrs)		Schizonts (30-42hrs)		Overall (72hrs)		Peak activity
	IC_{50}	IC_{90}	IC_{50}	IC_{90}	IC_{50}	IC_{90}	IC_{50}	IC_{90}	
ZY19489	9.0 ± 0.6	15.3 ± 0.9	28.3 ± 1.8	85.5 ± 4.3	12.8 ± 1.4	32.9 ± 2.9	6.0 ± 0.5	16.5 ± 0.6	Rings and schizonts
Dihydroartemisinin	1.7 ± 0.3	3.1 ± 0.4	2.9 ± 0.5	4.6 ± 0.4	10.7 ± 1.2	23.1 ± 2.0	1.2 ± 0.2	2.7 ± 0.4	Rings and trophozoites
Chloroquine	16.1 ± 1.9	31.6 ± 2.5	12.3 ± 1.6	24.8 ± 2.2	54.6 ± 2.9	101.8 ± 5.4	9.4 ± 1.1	17.9 ± 1.3	Rings and trophozoites
MMV390048	96.7 ± 4.1	154.3 ± 8.9	65.6 ± 4.3	117.1 ± 4.7	24.4 ± 3.0	43.6 ± 3.0	25.1 ± 2.4	46.3 ± 3.7	Schizonts

Data show means ± SEM, calculated from 4-5 independent experiments with technical duplicates. 3D7 parasites were tightly synchronized and drugs washed off after every stage-specific 12 hours of incubation. Gray shadings highlight the most potent *in vitro* activity window of each compound.

Supplementary Table 14: Averaged log_2 fold change of the baseline metabolite levels in synchronized trophozoite stage 3D7 and Dd2 parasites treated for 2.5 hours with 10× atovaquone or 10× ZY19489 IC_{50} compared to untreated controls. The heatmap shows increases and decreases of metabolites in drug- treated versus untreated controls in shades of red and blue, respectively.

Metabolic product (Dd2)	Dd2		Metabolic product (3D7)	3D7	
	ATQ-treated	ZY19489-treated		ATQ-treated	ZY19489-treated
1_3-Bisphosphoglyceric acid	-0.3272	-0.0963	2_3-Diphosphoglyceric acid	-0.0951	-0.3226
2-Hydroxy-2-methylbutanedioic acid	-0.4708	-0.0226	2-dehydro-D-gluconate	-0.0558	0.0708
3-phosphoglycerate	-0.3441	-0.0436	3-phosphoglycerate	-0.1427	-0.0291
3-phospho-serine	-0.2172	-0.4865	3-phospho-serine	-0.1177	-0.3124
4-aminobutyrate	0.2720	0.1718	4-aminobutyrate	-0.1012	-0.0738
4-phosphopantetheine	-0.4340	-0.5138	4-phosphopantetheine	-0.0486	-0.2079
4-phosphopantothenate	-0.3974	0.0002	4-phosphopantothenate	-0.4316	-0.0821
4-Pyridoxic acid	-0.1898	-0.1843	4-Pyridoxic acid	-0.3992	-0.3104
6-phospho-D-gluconate	0.6896	-0.1172	5-phosphoribosyl-1-pyrophosphate	-0.5018	-0.0986
acetyl-aspartate	-0.3376	-0.1279	6-phospho-D-gluconate	-0.0787	-0.1876
acetyl-CoA	-0.7570	-0.0134	acetyl-aspartate	-0.3469	-0.0153
Acetyl lysine	-0.2491	-2.4881	acetyl-CoA	-0.5165	-0.2056
acetyl phosphate	-0.7704	-0.2035	acetyl phosphate	0.3325	0.0576
Aconitate	-0.8591	-0.1055	aconitate	-0.8859	0.0070
ADP-D-glucose	-0.1690	-0.0640	ADP-D-glucose	-0.1547	-0.1794
a-ketoglutarate	-0.3756	-0.0880	a-ketoglutarate	-0.2010	0.0548
alanine/sarcosine	-0.2520	-1.2736	alanine/sarcosine	-0.4831	-0.1614
Arginine	0.3590	-0.1009	arginine	-0.0165	0.1299
Asparagine	0.0900	0.0195	asparagine	-0.1198	0.0895
Aspartate	-1.1864	-0.1320	aspartate	-0.7996	-0.0147
cCMP	-0.2489	0.1433	CDP	-0.4866	0.3909
CDP	-0.1422	0.1397	CDP-choline	-0.1001	-0.1720
CDP-choline	-0.1064	-0.2612	CDP-ethanolamine	-0.3218	-0.2372
CDP-ethanolamine	-0.3325	-0.0093	citrate/isocitrate	-0.9798	0.0831
cGMP	-0.3311	-2.9972	CMP	0.0484	0.0946
Citraconic acid	-0.5210	-0.1302	coenzyme A	-0.3216	-0.3346
citrate/isocitrate	-0.9566	-0.3548	creatine	-0.6825	-0.2860
CMP	0.2013	-0.0056	CTP	-0.7666	-0.1376
coenzyme A	-0.3348	-0.3528	cyclic-AMP	-0.4402	-0.4176
Creatine	-0.4211	0.0945	cytidine	-0.0568	0.0819
CTP	-0.3631	0.1043	dAMP	0.1941	0.1277
cyclic-AMP	-0.1580	-0.2333	dATP	0.2711	-0.2869
Cytidine	-0.0105	0.0984	dCMP	0.1698	0.2900
**DAL**	-1.0114	-2.7374	dCTP	-0.3735	-0.1201
dAMP	-0.5029	-0.1569	Decanoyl-CoA	0.0892	0.3054
dATP	-0.3282	-0.0475	D-erythrose-4-phosphate	0.1589	-0.3197
dCDP	0.0598	-0.1657	dGDP/ADP	0.1487	0.1094
dCMP	-0.0901	-0.1988	dGMP/AMP	0.1448	0.2427
dCTP	-0.2409	0.0218	dGTP/ATP	-0.0731	-0.2634
Decanoyl-CoA	0.3139	-0.1432	**dihydroorotate**	4.6234	-0.5563
D-erythrose-4-phosphate	0.8567	0.1837	dihydroxy-acetone-phosphate	-0.0700	-0.3236
dGDP/ADP	-0.0296	-0.0062	**DLH**	-0.3769	-1.2731

D-gluconate	-0.2042	-0.1517
D-glucosamine-1/6-phosphate	-1.1396	-0.3518
dGMP/AMP	0.1348	-0.0038
dGTP/ATP	-0.2069	-0.0939
**dihydroorotate**	5.6624	-0.3708
dihydroxy-acetone-phosphate	-0.0851	0.0129
**DLH**	-0.4598	-3.1293
DL-Pipecolic Acid	-0.1460	-1.3477
**DLS**	-0.4635	-2.8879
D-sedoheptulose-1/7-phosphate	0.3576	0.0697
dTDP	-0.5650	-0.0658
dTMP	-0.0109	-0.1897
dTTP	-0.7274	-0.0829
dUMP	-3.8344	1.7642
FAD	-0.0841	-0.1184
FMN	-0.6773	-0.0180
folate	-0.2235	-0.2118
fructose-1-6-bisphosphate	-0.3183	-0.0303
fumarate	1.2540	-0.0155
GDP	-0.0283	0.0024
glucono-lactone	-0.3946	-0.1772
glucose-1-phosphate	0.0392	-0.0760
glutamate	-0.0163	-0.0140
glutamine	0.2159	0.0342
glutathione	-0.7243	-0.6486
glutathione disulfide	-0.1892	-0.1351
GMP	0.0950	-0.0287
GTP	-0.0315	0.0026
guanine	0.0773	-0.0097
Hexose	-0.0225	-0.1435
hexose-phosphate	-0.1953	0.0102
histidine	-0.0452	-0.3009
**HVDD**	-0.5244	-2.8921
hydroxyproline/Aminolevulinate	-0.0480	0.0427
hypoxanthine	-0.1274	-0.1451
IMP	-0.2290	-0.1346
Indole	-0.1437	-0.2422
isoleucine	-0.1050	-0.1176
isovaleryl/2-methylbutyryl-CoA	-1.2656	-0.0693
lactate	0.5245	0.6554
L-arginino-succinate	0.3572	0.3078
**LD**	0.0234	-0.4516
leucine	-0.0189	-0.0991
lysine	0.4461	-1.1746
malate	0.9375	-0.0132
maleic acid	0.9315	-0.0157
methionine	-0.0510	-0.1369
myo-inositol	-0.2575	-0.1349
Myristoyl/tetradecanoyl-CoA	-0.3392	0.1435
N-acetyl-glucosamine-1/6-phosphate	-0.0202	-0.1338
N-acetyl-glutamate	-0.4040	-0.1251
N-acetyl-glutamine	-0.1070	0.3276
N-Acetyl-L-alanine	-0.1720	-0.2203
N-acetyl-L-ornithine	-0.2575	-2.4330
NAD+	-0.2848	-0.1136
NADH	0.5491	-0.0753
NADP+	-0.2020	-0.1602

D-sedoheptulose-1/7-phosphate	-0.0350	-0.0600
dTDP	-0.8208	0.3405
dTMP	0.2509	0.6598
dTTP	-1.3274	-0.1930
FAD	-0.0394	-0.1680
FMN	-0.4187	-0.3229
folate	-0.1144	-0.0951
fructose-1-6-bisphosphate	-0.1621	-0.0103
fumarate	-0.0714	0.1781
GDP	0.3518	0.1869
glucono-lactone	-0.2295	-0.1109
glucose-1-phosphate	-0.1576	-0.3136
glutamate	-0.0015	-0.1519
glutamine	-0.1971	0.0015
glutathione	-0.4977	-1.0004
glutathione disulfide	-0.2493	-0.3142
GMP	0.3089	-0.0372
GTP	0.1364	-0.2495
guanine	0.0368	0.1063
Hexose	-0.1782	-0.0410
hexose-phosphate	-0.2317	-0.1161
histidine	-0.2672	-0.2136
**HVDD**	-0.6157	-1.1691
hydroxyproline/Aminolevulinate	-0.2287	-0.0147
Hypoxanthine	0.0014	-0.1271
IDP	-0.2450	-0.0158
IDS	-0.4989	-1.2237
IMP	0.0209	-0.1892
Isoleucine	-0.2336	-0.0046
L-arginino-succinate	0.2240	0.3623
**LD**	-0.3057	-1.0124
Leucine	-0.1934	0.0351
Lysine	0.7929	-0.0404
Malate	-0.0575	-0.1384
maleic acid	-0.0418	-0.1292
Methionine	-0.0829	0.0835
myo-inositol	0.1228	0.6391
Myristoyl/tetradecanoyl-CoA	-0.2599	-0.4436
N-acetyl-glucosamine-1/6-phosphate	0.3104	-0.3262
N-acetyl-glutamate	-0.1090	-0.0997
N-Acetyl-L-alanine	0.0349	0.1224
NAD+	-0.0874	-0.1673
NADH	-0.1828	-0.2288
NADP+	-0.2019	-0.2017
NADPH	-0.4285	-0.3220
**N-carbamoyl-L-aspartate**	3.8307	-0.3967
Nicotinate	0.1609	0.0067
Nicotinic acid mononucleotide	-0.1612	-0.2264
Octulose 8/1P	-0.7437	-0.4702
Octulose Bisphosphate	-0.2573	0.3247
**PD**	-0.0288	-0.5647
**PE**	-0.0968	-0.5701
**PEE**	-0.5147	-0.4867
**PEEK**	-0.5594	-0.6722
phenylalanine	0.0233	-0.0237
phosphoenolpyruvate	-0.1055	-0.1070
**PVNF**	-0.6692	-1.5201

NADPH	-0.2528	-0.0370
**N-carbamoyl-L-aspartate**	5.5000	-0.5314
nicotinate	0.1273	0.1468
Nicotinic acid mononucleotide	-0.3029	-0.0728
Octulose Bisphosphate	-0.1682	0.0682
Ornithine	0.0410	-0.0706
**PD**	-0.3040	-1.9694
**PE**	-0.3940	-2.6082
**PEE**	-0.4550	-2.5010
**PEEK**	-0.5019	-2.4020
phenylalanine	-0.1135	-0.1396
phosphoenolpyruvate	-0.5260	-0.0435
Proline	0.0595	-0.4741
**PTT**	-0.4503	-3.7081
**PVNF**	-0.5072	-3.0000
Pyridoxine	-0.1496	-0.1892
Pyroglutamic acid	-0.1453	-0.1297
Pyrophosphate	0.0457	0.1068
Riboflavin	-0.3562	-0.1536
Ribose	-0.0438	-0.1587
Ribose-5-phosphate	-0.0834	-0.2265
ribose-phosphate	-0.0611	-0.1582
ribulose-5-phosphate	-0.2718	-0.1123
S-adenosyl-L-homocysteine	0.2087	0.2147
**SD**	-0.5277	-1.9277
**SDL**	-0.4644	-3.4225
Sedoheptulose bisphosphate	0.0022	0.0281
Serine	0.1141	-0.0145
S-methyl-5'-thioadenosine	-0.0968	-0.1983
sn-glycerol-3-phosphate	0.4525	-0.1163
succinate/Methylmalonic acid	0.1629	-0.1356
Taurine	-0.6973	-0.1420
Thiamine	-0.2124	-0.1570
Thiamine pyrophosphate	-0.0202	0.0054
threonine/homoserine	-0.0344	-0.1271
Tryptophan	-0.1665	-0.1670
Tyrosine	-0.1113	-0.1571
UDP	-0.3196	0.0327
UDP-D-glucose	-0.2140	-0.0170
UDP-D-glucuronate	-0.2204	-0.1414
UDP-N-acetyl-glucosamine	-0.2189	-0.0746
UMP	-0.2589	-0.0250
Uridine	0.1114	0.4098
UTP	-0.5680	-0.0005
Valine	0.2112	0.0148
**VD**	-0.3822	-1.8524
**VDPVNF**	-0.5923	-2.4857
Xanthosine	-0.8788	0.0718
xanthosine-5-phosphate	-0.2870	-0.0487

pyridoxine	-0.6954	0.0237
Pyroglutamic acid	0.0421	-0.0951
Pyrophosphate	-0.2375	-0.2430
Riboflavin	0.0613	-0.0886
Ribose	-0.3286	-0.0894
Ribose-5-phosphate	-0.1510	-0.3203
ribose-phosphate	-0.2025	-0.1906
ribulose-5-phosphate	-0.0263	-0.1485
S-adenosyl-L-homocysteine	-0.0198	-0.0610
**SD**	-0.4686	-0.7035
Sedoheptulose bisphosphate	-0.1832	0.4685
Serine	-0.1533	-0.0647
SID	-0.7511	-0.4964
sn-glycerol-3-phosphate	0.0799	-0.2584
succinate/Methylmalonic acid	-0.6777	-0.1980
succinyl-CoA/methylmalonyl-CoA	-0.5613	-0.1313
Thiamine	-0.1359	-0.1947
Thiamine pyrophosphate	-0.1160	-0.1722
threonine/homoserine	-0.2862	-0.2238
Tryptophan	-0.0198	-0.1234
Tyrosine	-0.1378	-0.0488
UDP	-1.1218	0.2583
UDP-D-glucose	-0.1496	-0.1005
UDP-D-glucuronate	-0.1360	-0.1302
UDP-N-acetyl-glucosamine	-0.3075	-0.1333
UMP	-0.5236	0.0523
UTP	-1.6485	-0.2821
Valine	-0.3153	-0.5067
**VD**	-0.2839	-0.1492
**VDPVNF**	-0.6914	-1.5966
xanthosine-5-phosphate	-0.2363	0.0646

Supplementary Table 15: **Characteristic features of the short hemoglobin (Hb)-derived peptides identified from synchronized trophozoite stage 3D7 and Dd2 parasites treated for 2.5 hours with 10× ZY19489 IC_{50} (100 nM).**

Strain	Peptide ID	Amino acid length	Net charge		Isoelectric point
			pH 5.5	pH 7.4	
3D7 and Dd2	DLH	3	-0.22	-0.97	5.08
3D7 and Dd2	SD	2	-0.98	-1.00	4.30
3D7 and Dd2	VDPVNF	6	-0.98	-1.00	4.05
3D7 and Dd2	PVNF	4	0.00	-0.01	5.95
3D7 and Dd2	PEEK	4	-0.89	-1.00	4.53
3D7 and Dd2	PE	2	-0.95	-1.00	4.60
3D7 and Dd2	PD	2	-0.98	-1.00	4.30
3D7 and Dd2	LD	2	-0.98	-1.00	4.30
3D7 and Dd2	HVDD	4	-1.19	-1.97	4.51
3D7 and Dd2	VD	2	-0.98	-1.00	4.30
3D7 and Dd2	PEE	3	-1.89	-2.00	4.24
Dd2	SDL	3	-0.98	-1.00	4.05
Dd2	DLS	3	-0.98	-1.00	4.05
Dd2	DAL	3	-0.98	-1.00	4.05
Dd2	PTT	3	0.00	-0.01	5.95

Supplementary Table 16: Hemoglobin, 'free' heme and hemozoin levels in synchronized NF54 parasites treated for 30 hours with various concentrations of ZY19489, chloroquine (CQ) and pyrimethamine (PYR).

ZY19489 treatment	Amount of hemoglobin (fg/cell)	Amount of free heme (fg/cell)	Amount of hemozoin (fg/cell)
No drug control	0.91 ± 0.1	5.63 ± 0.2	65.86 ± 1.0
0.5× IC_{50} (4 nM)	1.09 ± 0.1	5.96 ± 0.2	64.46 ± 1.7
1.0× IC_{50} (8 nM)	1.95 ± 0.2	6.10 ± 0.2	62.68 ± 1.1
1.5× IC_{50} (12 nM)	2.04 ± 0.3*	6.01 ± 0.2	62.40 ± 1.4*
2.0× IC_{50} (16 nM)	1.97 ± 0.3*	5.94 ± 0.3	60.92 ± 1.7*
2.5× IC_{50} (20 nM)	2.46 ± 0.4*	5.96 ± 0.1	55.71 ± 0.9***

CQ treatment	Amount of hemoglobin (fg/cell)	Amount of free heme (fg/cell)	Amount of hemozoin (fg/cell)
No drug control	1.23 ± 0.1	5.55 ± 0.2	65.38 ± 1.9
0.5× IC_{50} (5 nM)	1.15 ± 0.1	5.92 ± 0.2	57.55 ± 1.7
1.0× IC_{50} (10 nM)	1.31 ± 0.1	6.88 ± 0.3*	55.92 ± 2.3
1.5× IC_{50} (15 nM)	2.05 ± 0.2**	7.34 ± 0.2*	53.38 ± 1.6*
2.0× IC_{50} (20 nM)	1.85 ± 0.2*	8.17 ± 0.2*	51.81 ± 1.9*
2.5× IC_{50} (25 nM)	0.91 ± 0.1**	8.32 ± 0.3*	49.60 ± 0.5*

PYR treatment	Amount of hemoglobin (fg/cell)	Amount of free heme (fg/cell)	Amount of hemozoin (fg/cell)
No drug control	1.55 ± 0.1	4.40 ± 0.3	66.98 ± 5.1
0.5× IC_{50} (4 nM)	1.89 ± 0.2	4.91 ± 0.3	56.12 ± 2.1
1.0× IC_{50} (8 nM)	1.70 ± 0.3	3.12 ± 0.4	61.06 ± 2.4
1.5× IC_{50} (12 nM)	2.23 ± 0.2	4.41 ± 0.2	62.22 ± 2.2
2.0× IC_{50} (16 nM)	2.05 ± 0.2	3.46 ± 0.3	61.75 ± 3.1
2.5× IC_{50} (20 nM)	1.92 ± 0.3	5.72 ± 0.2	65.36 ± 2.5

Mean ± SEM amounts of hemoglobin, free heme and hemozoin are represented as fg/cell. The amounts of different heme species were determined by the heme fractionation assay (see Methods). NF54 parasites were treated for 30 hours with increasing concentrations of ZY19489, CQ or PYR (at different multiples of their IC_{50} values) and hemoglobin, free heme and hemozoin amounts measured in mature harvested trophozoites. Data were calculated from 4 to 8 values per concentration. Statistical comparisons of the drug-treated lines to their untreated controls were performed using two-tailed Mann-Whitney *U* tests where $*p = 0.0286$, $**p = 0.0011$ and $***p = 0.0002$.

Supplementary Table 17: Names and sequences of oligonucleotides used in the editing of *pfcrt* N246H and *pfapc10* D233N mutations

Name	Lab code	Oligonucleotide sequence	Description
p1	p9142	ATAAGATTGACATTTTAAGATTAcATGCTATGGTATCCTTTTTCC	PfCRT Dd2 N246H SDM Fwd
p2	p9182	GGAAAAAGGATACCATAGCATgTAATCTTAAAATGTCAATCTTAT	PfCRT Dd2 N246H SDM Rev
p3	p3315	CTCGAGATGGTTGGTTCGCTAAACTGC	hdhfr (+1-21) Xho1 Fwd
p4	p3403	TTGACCCTTATATATTCCACCCA	PfCRT 3' UTR (+1285-1308)
p5	p3404	CTTGGGCCCAAGTTGTACTGCTTCTAAGC	PfCRT 5′ UTR seq primer Fwd
p6	p3265	CTTATCGATAAGCAGAAGAACATATTAATAGGAATACTTAATTG	PfCRT exon 3 Rev
p7	p3264	CTTGAATTCGACCTTAACAGATGGCTCAC	PfCRT exon 2 EcoRI Fwd
p8	p1640	AACCATGGATTTATTGTGTAATAATTGAATCGACG	PfCRT exon 13 seq primer Rev
p9	p8868	TGAACAGTTGGATATACTTGACTCT	PfAPC10 seq primer Fwd
p10	p8869	TGTAGTTGCCTTATTCGTGTGTC	PfAPC10 3' UTR seq primer Rev

SDM; site-directed mutagenesis, Fwd; forward, Rev; reverse, seq; sequencing, UTR; untranslated region, hdhfr; human dihydrofolate reductase, PfCRT; *P. falciparum* chloroquine resistance transporter, PfAPC10; *P. falciparum* anaphase promoting complex 10.
