# Supplementary material for: Collateral hypersensitivity between ZY19489 and piperaquine neutralizes PfCRT-mediated drug efflux and Plasmodium falciparum resistance
Source: Nat Commun. 2026 Apr 1;17:5441. doi: 10.1038/s41467-026-70914-1 (PMC13280021; doi:10.1038/s41467-026-70914-1)
Supplement: Supplementary file 2 — Reporting summary [file 41467_2026_70914_MOESM2_ESM.pdf]

nature portfolio

Corresponding author(s): David A. Fidock

Last updated by author(s): Mar 2, 2026

Reporting Summary

Nature Portfolio wishes to improve the reproducibility of the work that we publish. This form provides structure for consistency and transparency in reporting. For further information on Nature Portfolio policies, see our Editorial Policies and the Editorial Policy Checklist.

Statistics

For all statistical analyses, confirm that the following items are present in the figure legend, table legend, main text, or Methods section.

n/a	Confirmed	
☐	☒	The exact sample size (n) for each experimental group/condition, given as a discrete number and unit of measurement
☒	☐	A statement on whether measurements were taken from distinct samples or whether the same sample was measured repeatedly
☐	☒	The statistical test(s) used AND whether they are one- or two-sided<br>*Only common tests should be described solely by name; describe more complex techniques in the Methods section.*
☒	☐	A description of all covariates tested
☒	☐	A description of any assumptions or corrections, such as tests of normality and adjustment for multiple comparisons
☐	☒	A full description of the statistical parameters including central tendency (e.g. means) or other basic estimates (e.g. regression coefficient) AND variation (e.g. standard deviation) or associated estimates of uncertainty (e.g. confidence intervals)
☐	☒	For null hypothesis testing, the test statistic (e.g. F, t, r) with confidence intervals, effect sizes, degrees of freedom and P value noted<br>*Give P values as exact values whenever suitable.*
☒	☐	For Bayesian analysis, information on the choice of priors and Markov chain Monte Carlo settings
☒	☐	For hierarchical and complex designs, identification of the appropriate level for tests and full reporting of outcomes
☒	☐	Estimates of effect sizes (e.g. Cohen's d, Pearson's r), indicating how they were calculated

Our web collection on statistics for biologists contains articles on many of the points above.

Software and code

Policy information about availability of computer code

Data collection	Metabolomics data acquired from the mass spectrometry were imported into the el-MAVEN software package for peak picking. No codes were used during data collection. Flow cytometry data was collected using Intellicyt iQue3 (Essen Bioscience)
Data analysis	GATK HaplotypeCaller (version 3.8) was used to identify all variants in resistant clones. Molecular docking was performed using GOLD software and molecular dynamics simulations analyzed using CPPTRAJ and MDAnalysis (release 2.10.0). No codes were used.

For manuscripts utilizing custom algorithms or software that are central to the research but not yet described in published literature, software must be made available to editors and reviewers. We strongly encourage code deposition in a community repository (e.g. GitHub). See the Nature Portfolio guidelines for submitting code & software for further information.

Data

Policy information about availability of data

All manuscripts must include a data availability statement. This statement should provide the following information, where applicable:

- Accession codes, unique identifiers, or web links for publicly available datasets
- A description of any restrictions on data availability
- For clinical datasets or third party data, please ensure that the statement adheres to our policy

The metabolomic data generated in this study are publicly accessible through the NCBI Metabolomics Workbench under the Project ID: PR002514. The raw reads of WGS data generated in this study have been deposited in the NCBI Sequenced Read Archive (SRA) under BioProject accession number PRJNA1376518 and are

accessible via the link https://www.ncbi.nlm.nih.gov/bioproject/1376518. Other datasets analyzed during the current study are provided with this paper as Supplementary Material (Supplementary Figures 1 - 12 and Supplementary Tables 1 – 17) and Source Data File.

Research involving human participants, their data, or biological material

Policy information about studies with human participants or human data. See also policy information about sex, gender (identity/presentation), and sexual orientation and race, ethnicity and racism.

Reporting on sex and gender	For routine P. falciparum culturing, RBCs were purchased from the Interstate Blood Bank (Memphis, TN) as pooled, de-identified, anonymized blood that was washed to remove any residual leukocytes. Anonymized pooled serum for parasite culture was obtained from Interstate Blood Bank Inc or New York Blood Center. Due to this anonymization, there was no information on sex and gender.
Reporting on race, ethnicity, or other socially relevant groupings	No information available as the donors of the human blood and serum were anonymous.
Population characteristics	No information available as the donors of the human blood and serum were anonymous.
Recruitment	No information available as the donors of the human blood and serum were anonymous.
Ethics oversight	For the studies performed at The Art of Discovery using NSG mice, which were engrafted with human erythrocytes obtained from Centro Vasco de Transfusiones y Tejidos Humanos (Galdakao, Basque Country, Spain), Centro de Transfusiones de la Comunidad de Castilla y León, (Valladolid, Spain), Centro de Transfusión de la Comunindad de Madrid, (Madrid, Spain) and the Banc de Sang I Teixits (Barcelona, Spain). The human biological samples were sourced ethically, and their research use was in accord with the terms of the informed consents. For routine P. falciparum culturing, human RBCs and serum were purchased from the Interstate Blood Bank (Memphis, TN) or New York Blood Center as pooled, de-identified, anonymized blood or serum that was washed to remove any residual leukocytes. Approval for this protocol (AAAU3761) was provided on 28 October 2022 by the Columbia University Institutional Review Board, which deemed this work to be Not Human Subjects Research under 45 CFR 46.

Note that full information on the approval of the study protocol must also be provided in the manuscript.

Field-specific reporting

Please select the one below that is the best fit for your research. If you are not sure, read the appropriate sections before making your selection.

☒ Life sciences ☐ Behavioural & social sciences ☐ Ecological, evolutionary & environmental sciences

For a reference copy of the document with all sections, see nature.com/documents/nr-reporting-summary-flat.pdf

Life sciences study design

All studies must disclose on these points even when the disclosure is negative.

Sample size	No sample size calculations were done for this study since this was an in vitro characterization of resistant clones from known Plasmodium falciparum strains.
Data exclusions	No data was excluded from any of the analyses
Replication	Reproducibility was attained through multiple independent biological and technical repetition of the data. In most instances, we repeated our experiements on at least 4 different occassions t ascertain reproducibility.
Randomization	Randomization was only done when assigning the mice into treatment versus untreated groups. For these experiments, controlling for covariates was not relevant because random assignment ensured that both observable and unobservable baseline characteristics are balanced or statistically equivalent between the treated and untreated groups of mice.
Blinding	Investigators were blinded to the in vivo experiements since the technician administering the treatment differed from the scientist who analyzed the treatment outcomes in the mice.

Reporting for specific materials, systems and methods

We require information from authors about some types of materials, experimental systems and methods used in many studies. Here, indicate whether each material, system or method listed is relevant to your study. If you are not sure if a list item applies to your research, read the appropriate section before selecting a response.

Materials & experimental systems

n/a	Involved in the study	
☐	☒	Antibodies
☐	☒	Eukaryotic cell lines
☒	☐	Palaeontology and archaeology
☐	☒	Animals and other organisms
☒	☐	Clinical data
☒	☐	Dual use research of concern
☒	☐	Plants

Methods

n/a	Involved in the study	
☒	☐	ChIP-seq
☐	☒	Flow cytometry
☒	☐	MRI-based neuroimaging

Antibodies

Antibodies used	1 Mouse anti-HA primary antibody from Sigma-Aldrich; catalog number H3663; HA-7 clone. 2. Rabbit anti-GAPDH primary antibody from Sigma-Aldrich; catalog number G9545.
Validation	Western blot validation.

Eukaryotic cell lines

Policy information about cell lines and Sex and Gender in Research

Cell line source(s)	Plasmodium falciparum Dd2 strain originally donated by Dr. Thomas Wellems
Authentication	Whole genome sequencing
Mycoplasma contamination	No mycoplasma contamination
Commonly misidentified lines (See ICLAC register)	Not applicable

Animals and other research organisms

Policy information about studies involving animals; ARRIVE guidelines recommended for reporting animal research, and Sex and Gender in Research

Laboratory animals	The in vivo efficacy studies were performed in a standardized commercial model of P. falciparum infection in NOD-SCID IL-2Rynull (NSG) mice engrafted with human erythrocytes (TADhuMouse®-Pfalc). The NSG mice (Charles River, France) were engrafted with human erythrocytes obtained from Centro Vasco de Transfusiones y Tejidos Humanos (Galdakao, Basque Country, Spain), Centro de Transfusiones de la Comunidad de Castilla y León, (Valladolid, Spain), Centro de Transfusión de la Comunindad de Madrid, (Madrid, Spain) and the Banc de Sang I Teixits (Barcelona, Spain).
Wild animals	This study did not involve wild animals
Reporting on sex	For the in vivo efficacy studies, we used female NOD-SCID IL-2Rynull (NSG) mice engrafted with human erythrocytes
Field-collected samples	This study did not involve any field-collected samples
Ethics oversight	The studies were approved by The Art of Discovery Institutional Animal Care and Use Committee (TAD-IACUC), certified by the Biscay County Government (Bizkaiko Foru Aldundia, Basque Country, Spain) to evaluate animal research projects from Spanish institutions according to point 43.3 from Royal Decree 53/2013, from the 1st of February (BOE-A-2013–1337). All experiments were carried out in accordance with European Directive 2010/63/EU. Animal experiment results were reported following ARRIVE guidelines (https://www.nc3rs.org.uk/arrive-guidelines), except for disclosure of business trade confidential information.

Note that full information on the approval of the study protocol must also be provided in the manuscript.

Plants

Seed stocks	Not applicable since this study did not involve any plants
Novel plant genotypes	Not applicable since this study did not involve any plants
Authentication	Not applicable since this study did not involve any plants

Flow Cytometry

Plots

Confirm that:

☒ The axis labels state the marker and fluorochrome used (e.g. CD4-FITC).

☒ The axis scales are clearly visible. Include numbers along axes only for bottom left plot of group (a 'group' is an analysis of identical markers).

☒ All plots are contour plots with outliers or pseudocolor plots.

☒ A numerical value for number of cells or percentage (with statistics) is provided.

Methodology

Sample preparation	To detect surviving P. falciparum parasites from drug assays and to quantify parasitemia from the growth rate experiments, SYBR Green I (Invitrogen) and MitoTracker Deep Red FM (Invitrogen) were used as stains for DNA and cell viability, respectively.
Instrument	Parasite survival was assessed by flow cytometry on an Intellicyt iQue3 (Essen Bioscience)
Software	FlowJo version 10 (FlowJo LLC) was used for analysis of parasite proliferation
Cell population abundance	Flow cytometry was used to quantify parasite survival in the presence of various drugs at a range of different concentrations and to quantify parasite growth rates. The parasitemia (% of red blood cells containing live parasites) ranged from 0% to 6% in different samples. Cells were not sorted for these assays.
Gating strategy	The gating strategy used in our study has been described previously by Straimer et al.,2015. Briefly this involved first gating red blood cells (unifected and infected) using forward scatter (FSC) and side scatter (SSC) channels. Red blood cells infected with parasites were observed as positive events in the FL1 (SYBR Green I) and FL4 (Mito Tracker Deep Red) channels in the upper right quadrant gate of the plot.

☒ Tick this box to confirm that a figure exemplifying the gating strategy is provided in the Supplementary Information.
