# Supplementary material for: Collateral hypersensitivity between ZY19489 and piperaquine neutralizes PfCRT-mediated drug efflux and Plasmodium falciparum resistance
Source: Nat Commun. 2026 Apr 1;17:5441. doi: 10.1038/s41467-026-70914-1 (PMC13280021; doi:10.1038/s41467-026-70914-1)
Supplement: Supplementary file 3 — Transparent Peer Review file [file 41467_2026_70914_MOESM3_ESM.pdf]

nature portfolio

Peer Review File

Collateral hypersensitivity between ZY19489 and piperaquine neutralizes PfCRT-mediated drug efflux and Plasmodium falciparum resistance

Corresponding Author: Professor David Fidock

This file contains all reviewer reports in order by version, followed by all author rebuttals in order by version.

Version 0:

Reviewer comments:

Reviewer #1

(Remarks to the Author)
The authors have investigated the mechanism of resistance and the mode of action of the antimalarial clinical development candidate ZY19489. A comprehensive set of experiments was conducted, including in vivo selection of resistant parasites, whole-genome sequencing, QTL analysis of a genetic cross, and in vivo mutagenesis. These independent approaches all converge on PfCRT as the principal determinant of resistance.
While the identification of PfCRT as a resistance determinant is not inherently surprising—given its known structural flexibility and polyspecific substrate recognition—what makes this study noteworthy is the discovery that the PfCRT variant conferring resistance to ZY19489 loses the ability to transport chloroquine (CQ) and piperaquine (PPQ). This finding of inverse susceptibility is of considerable interest, as it raises the possibility of an evolutionary trap, in which the use of two drugs with opposing PfCRT susceptibilities could limit the evolution of cross-resistance.

Points to Be Addressed

1. Quality of the structural models (Figure 2)
Additional information is required to assess the robustness of the molecular models presented in Figure 2. Specifically, please include:
i) graphical representations of RMSD for both the protein backbone and the ligand across all simulated conformations;
ii) the docking scores for each pose;
iii) solvent-accessible surface area (SASA) values; and
iv) plots showing the distances between the ligand and key interacting residues as a function of simulation time.
2. Clarification regarding hydrogen bond persistence (Figure 2)
The figure legend states that "a significant hydrogen bond persisted for more than 30% of the simulation time." This implies that the interaction was absent for approximately two-thirds of the trajectory. Please clarify the interpretation: does this mean the hydrogen bond is transient and thus not functionally significant?
3. Molecular dynamics simulations for ZY19489 versus CQ and PPQ
To better understand the mechanistic basis of the observed inverse susceptibility, additional MD simulations should be performed comparing ZY19489, CQ, and PPQ. Do these ligands occupy distinct regions of the substrate-binding cavity, or are their binding poses overlapping? Is there evidence of steric hindrance or mutual exclusion between them?
4. Verification of metabolites in P. falciparum extracts
Please clarify whether the proposed metabolites were experimentally verified in the context of P. falciparum extracts. This is an important control, as many metabolites display altered migration or detection profiles due to matrix effects from parasite extracts.
5. Experimental testing of the evolutionary trap hypothesis
Have the authors attempted to test the evolutionary trap hypothesis experimentally—for example, by exposing parasites to combinations of ZY19489 with CQ or PPQ? Such data would significantly strengthen the manuscript's central claim.
6. Discussion of literature on subverted inverse susceptibility
There are documented cases where inverse susceptibility was eventually overcome by cross-resistance, especially in proteins with high mutational plasticity such as PfCRT. The authors should acknowledge and discuss this possibility, citing relevant work (e.g., Mandt et al., eLife 2023, DOI: 10.7554/eLife.85023; Nichol et al., Nat Commun 2019, DOI:

10.1038/s41467-018-08098-6).

Overall Assessment

This is a well-designed and timely study that contributes valuable insights into the mechanisms of antimalarial resistance and the structural dynamics of PfCRT. The concept of an evolutionary trap mediated by inverse susceptibility is particularly intriguing and potentially impactful. Addressing the points outlined above—especially those concerning the molecular modeling data and experimental verification of key hypotheses—would substantially strengthen the manuscript and broaden its relevance.

Reviewer #2

(Remarks to the Author)
Okombo et al describe the characterisation of the mode of resistance and the mode of action of ZY19489, a new antimalarial compound currently in phase II clinical trials. By generating resistant parasites in vitro they discover a novel point mutation N246H in the well-studied PfCRT protein. They convincingly show by multiple approaches including the generation of genome edited parasite lines in different genomic backgrounds that this mutation confers low-level resistance shifting the IC50 by about 12-fold. QTL analysis confirms that PfCRT mutations can modulate ZY19489 activity. No resistance was obtained upon infection and treatment of humanised mice. Based on the available cryo-EM structure they model the consequence of the N246H mutation on PfCRT and the binding of ZY19489. Most interestingly, they observe that the N246H mutation confers sensitivity to aminoquinoline antimalarials and show that ZY19489 competes with chloroquine and piperaquine in CRT drug efflux. The N246H mutation results in a severe growth phenotype and ZY19489 retains potency against many of the crt and mdr1 alleles present in the field, features that are very much in favour for a new potential antimalarial compound and opens interesting perspectives of potential drug combinations with counterbalancing effects of resistance selection.
The authors address the mode of action of ZY19489 through metabolomic analysis of treated parasites, identify several pathways and focus on Hb catabolism, given the resistance mechanism conferred by CRT. The results are complex. No direct action on candidate proteins like PfHDP or falcipain 2A or 2B could be detected. Hb peptides are increased but ZY19489 seems not to block beta-hematin formation nor increase free heme levels while there is some augmentation in Hb levels. The authors suggest a possible additional mode of action.

This is an excellently performed, controlled, described and presented study. Important new insights into PfCRT activity in resistance to different antimalarial compounds are identified and validated in a large number of genetic backgrounds confirming the complexity of genetic resistance to certain antimalarials and to ZY19489.

Minor comments:
- P4 line 91: please give a value of the previously observed low-level resistance
- P5 line 108: indicate how many clones were generated and sequenced
- P8 line 180ff: The conclusions here might need some more explanation. How can an occluded form facilitate drug transport? Is there a possibility that ZY19489 could enter the DV through CRT? Is the open-to-cytosol conformation just another term for closed-to-DV? If the mutation reduces ZY19489 interactions close to the cytosol side, do the authors conclude that ligand release into the cytosol is facilitated and would be the reason for resistance through faster transport?
- P10 Fig 3A: is this a bloated FV phenotype indicative of reduced Hb degradation?
- P10 line 245: has the N246H mutation been found in the 10,000 Pf field genomes?
- P11 in vivo experiments: There is no method section for these experiments. How many mice were treated? How many showed recrudescence? Which combinations at which concentrations were done? Perhaps a supp figure similar to S2 for the in vitro selection could help. What is the 3D7-0087/N9 strain? Why was 3D7 used here?
- P13 stage-dependent activity. I acknowledge the courage to present the observed relatively lower susceptibility of trophozoites to the drug. This observation is somewhat in contradiction to the predicted mode of action of ZY19489 through an effect on Hb degradation or possibly thymidine production that should be predominant in trophozoites. At the current state of knowledge this might be difficult to explain but these conflicting observations should as such briefly been mentioned in the discussion, P18 line 460.
- P21 resistance selection: please refer to Fig S2.
- P21 line 544: how many clones?
- P27 line 694: how many hours between Percoll and sorbitol treatment?
- P41 figure legend 4D: it would help understanding to indicate in the legend that rings were treated for 30h with drugs before analysis.
- Fig 4: I suggest to use the same scale (5-9 fg/cell) for free heme in panel 4H than in 4E to facilitate comparison. In 4H green data points could be connected by a dotted line for visibility.
- Supp Fig S1: panel B: how many hours after aTc? The decrease in GAPDH and HA might actually be similar. The loading control actually shows that the experiment didn't work, no parasite material in the -aTc sample.
- Supp Fig S3: for curiosity, N246H is located in which crt exon?

Reviewer #3

(Remarks to the Author)
The manuscript investigates the mechanism of action and resistance profile of the novel, long-acting antimalarial ZY19489. Using a combination of growth inhibition assays, metabolomics, heme-fractionation, microscopy, and genetic approaches, the authors show that ZY19489 rapidly kills Plasmodium falciparum asexual stages and affects hemoglobin catabolism in the digestive vacuole rather than directly inhibiting heme detoxification or folate/pyrimidine pathways.
In vitro resistance selection (particularly in a hypermutable Dd2-Polδ background) identifies a key mutation in the chloroquine resistance transporter PfCRT (N246H) that confers reduced susceptibility to ZY19489. This mutation, however, comes with a substantial fitness cost and creates strong collateral hypersensitivity to piperaquine and related aminoquinolines. Proteoliposome transport assays support a model in which ZY19489 is a PfCRT substrate and N246H alters its transport properties. In vivo experiments in humanized NSG mouse model show robust efficacy of ZY19489 and suggest a relatively high barrier to resistance under the dosing regimens tested. Based on the collateral sensitivity profile, the authors propose that pairing ZY19489 with piperaquine could act as an “evolutionary trap” and form a non-ACT, resistance-refractory combination therapy.

The link from PfCRT-mediated resistance to ZY19489, through to collateral hypersensitivity to piperaquine and other aminoquinolines, is logically developed and supported by both genetic and transport data. This provides a coherent mechanistic explanation and a plausible rationale for combination design.
This work directly addresses two critical issues for antimalarial development: (i) mechanism of action of a promising clinical candidate, and (ii) how resistance is likely to emerge and how it might be constrained by rational partner drug choice. The in vivo efficacy and resistance-selection data in humanized mouse models enhance the translational impact.

While the data indicate perturbation of hemoglobin uptake/catabolism and implicate PfCRT in resistance, the direct molecular target(s) of ZY19489 remain undefined. Some key mechanistic conclusions rely on correlative metabolomic and heme-fractionation patterns rather than direct measurements of endocytic or proteolytic steps.
The principal resistance mutation (N246H) arises in a hypermutator line under strong selection and carries a large fitness cost and pronounced collateral sensitivity. It is not yet clear how likely such a pathway would be to emerge and spread in natural parasite populations, and no epidemiological data are available.
The proposal to use ZY19489 with piperaquine as a resistance-refractory combination is mostly based on in vitro susceptibility and transport data. Dedicated in vivo combination and resistance-selection experiments for this specific pair are limited or absent, so the “trap” remains somewhat theoretical at this stage.
However, given its rigorous multidisciplinary experimentation, clear mechanistic and evolutionary insights into a clinically relevant antimalarial candidate, and strong translational implications for rational, resistance-limiting combination therapy design, I endorse this manuscript for publication in Nature Communications pending satisfactory revision.

Major point
The metabolomics data and heme-fractionation experiments are interpreted as showing that ZY19489 interferes with Hb endocytosis/catabolism rather than heme detoxification. However, the evidence is still somewhat indirect:
a) The decrease in short Hb-derived peptides after 2.5 h of treatment could reflect reduced peptide production, altered peptide export, or broader DV dysfunction, not necessarily a specific upstream step in Hb uptake or digestion.
b) The heme fractionation experiments show increased Hb and relatively unchanged “free” heme and hemozoin at relevant concentrations, but this pattern could also arise from changes in growth rate or DV morphology rather than a primary MoA.
c) Knockdown/KO of PfHDP and falcipains 2A/2B rules out those components, but the identity of the primary molecular target remains undefined.
Can the authors provide more direct experimental evidence that ZY19489’s primary antiparasitic activity is mediated through disruption of hemoglobin uptake/catabolism (e.g., live imaging of Hb endocytosis, measurement of DV endocytic flux, or genetic/chemical epistasis with known Hb-pathway mutants), or else temper the mechanistic claim accordingly?

The manuscript proposes PfCRT N246H as a key resistance determinant but also notes a 60–75% asexual blood-stage fitness cost and strong collateral sensitivity to multiple aminoquinolines:
a) N246H was selected in a hypermutable Dd2-Polδ line under prolonged high-dose pressure, which may not reflect natural selection conditions.
b) There is no current field surveillance evidence for N246H or closely related mutations in clinical isolates.
c) The claim that such mutants would be “rapidly outcompeted” in the field is plausible but not quantitatively supported.
Can the authors provide additional data or analysis addressing the in vivo relevance of PfCRT N246H (e.g., competition assays at lower starting frequencies, modeling of within-host or between-host dynamics, or a deeper scan of existing genomic datasets for N246H or compensatory mutations)? If not, the discussion could better distinguish between experimentally observed resistance pathways and their likely epidemiological probability.

The manuscript suggests that ZY19489 + PPQ could constitute an “evolutionary trap” and a resistance-refractory non-ACT combination, based mainly on:
a) In vitro collateral hypersensitivity of PfCRT N246H mutants to CQ/PPQ, and
b) Proteoliposome transport data showing ZY19489 inhibition of mutant PfCRT-mediated CQ/PPQ efflux.
However:
a) There are no in vivo combination efficacy or resistance-selection experiments specifically with ZY19489 + PPQ (only ferroquine, MMV693183, and M5717).

b) Potential safety concerns and known PPQ-associated resistance and cardiotoxicity issues are not discussed in detail.
c) It is not shown that long-term co-exposure cannot select other pfcrt or pfmdr1 variants that might erode this "trap".
Given that no in vivo data are presented for ZY19489 + PPQ, can the authors either (i) provide preliminary combination data (e.g., mouse efficacy or resistance-selection experiments, PK/PD compatibility) or (ii) clearly frame the ZY19489–PPQ combination as a theoretical proposal based on in vitro transport and susceptibility data, rather than a fully supported resistance-refractory strategy?

Minor points
a) In Table 1 the compound name appears as "ZY91489" rather than "ZY19489", which looks like a typographical error.
b) For several key datasets (metabolomics, heme fractionation, stage-specific activity, MD analyses), the exact number of biological replicates and the multiple-testing strategy are not always fully explicit in the main text.
c) Consider softening causal language (e.g., "suggests" rather than "demonstrates") where the link between metabolite pattern and mechanism is primarily correlative.
d) The claim of a "high in vivo barrier to resistance" is largely based on the absence of ZY19489-specific mutations in a limited number of recrudescent lines and combinations. It might be more precise to phrase this as "no overt resistance observed under the regimens tested" and acknowledge that longer selection or different dosing might still identify additional resistance pathways.
e) Ensure that all axes are clearly labeled with units (e.g., nM vs µM; fg/cell) and that symbols/colors in the legends match those in the panels (for example, some multi-compound curves in Fig. 4c and the supra-hex plots benefit from more explicit color labels in the legend).
f) Metabolomics data are stated to be deposited in Metabolomics Workbench; it would be helpful to also explicitly state where WGS data for selected lines and the NSG recrudescent lines are deposited (e.g., SRA/BioProject accession), or to commit to deposition.
g) A careful copy-editing pass would help with small inconsistencies (e.g., spacing around units, occasional missing hyphens such as "drug-selected", and minor grammatical issues). These do not affect scientific content but will improve readability.
h) In the Materials and Methods section, the notation for concentration units is inconsistent (e.g., use of both "w/w" and "wt/wt", and both "v/v" and "vol/vol"). Please standardize to a single format throughout (e.g., "% w/w" and "% v/v") in accordance with the journal's style.
i) Mitochondrial bc1 complex should be written as "mitochondrial bc1 complex".
j) Line 410, "in vivo" should be written in italic.
k) At 2.5× IC_{50}, ZY19489 treatment is shoen to cause a significant decrease in hemoglobin (Hb) levels (Fig 4i). Could the authors clarify why Hb levels are reduced specifically at 2.5× IC_{50} (e.g., stage-specific effects, timing of sampling, or assay sensitivity), and reconcile this with the broader model of DV Hb handling under ZY19489 exposure?
l) Can the authors be consistent in writing E64D, as in the figures, and not E64d in the text?
m) The metabolomics data show alterations in pyrimidine intermediates alongside DV-related changes. Since ZY19489 is proposed primarily to affect DV hemoglobin handling, it would be helpful if the authors could clarify whether the pyrimidine perturbations are interpreted as a secondary consequence of DV dysfuncton(e.g., impaired amino acid supply/energy status) rather than a direct effect on pyrimidine biosynthesis, and briefly outline the mechanistic link they envisage between DV damage and pyrimidine metabolism.

Version 1:

Reviewer comments:

Reviewer #1

(Remarks to the Author)
The authors have addressed my previous queries in a professional and objective manner, leading to improvements in clarity, content, and overall scope of the manuscript. However, an oversimplified—and in parts potentially incorrect—statement has been introduced in the revised version in response to my Comment 3 concerning ligand occupation of the substrate-binding cavity of PfCRT.
To address Comment 3, the authors added the following passage to the Discussion:

"In our simulations, ZY19489 consistently samples the same T76-adjacent region of the cavity, indicating that this molecule, CQ and PPQ engage largely overlapping binding volumes rather than distinct or spatially separated pockets. This interpretation aligns with our proteoliposome data where transport of CQ and PPQ by PfCRT variants that mediate resistance to these drugs was potently inhibited in the presence of ZY19489. This pattern suggests mutual exclusion which, in the context of PfCRT, does not imply steric hindrance but rather the intrinsic single-substrate nature of the transporter in which only one ligand can occupy the central cavity at a time and preclude binding or transport of another. The competition pattern observed experimentally is therefore consistent with all three compounds accessing the same substrate-binding cavity."

This paragraph raises several significant concerns:
1. Overgeneralization across PfCRT variants. Conclusions drawn primarily from simulations and uptake studies performed with PfCRTGB4 are extended broadly to other geographically distinct PfCRT variants without sufficient justification.

2. Failure to acknowledge structural and functional differences between PfCRT variants. The paragraph does not account for evidence indicating that PfCRTGB4 and PfCRTDd2 possess distinct substrate-binding cavity architectures. While PfCRTGB4 appears to accommodate only a single ligand at a time (either CQ or PPQ), as suggested by the authors' molecular dynamics simulations, PfCRTDd2 has been shown to bind both substrates simultaneously under a partial noncompetitive inhibition model. For PfCRTDd2, this interpretation is supported by extensive transport kinetics and complementary computational studies, which collectively suggest the presence of at least two distinct interaction sites within the binding cavity (PMID: 37285379; PMID: 25378409; PMID: 40140375; https://hdl.handle.net/1885/733780662).
3. Overinterpretation of transport data. The CQ and PPQ uptake data presented in Figures 2D and 2E are insufficient to support the conclusion that CQ, PPQ, and potentially ZY19489 act as mutually competitive inhibitors on PfCRTGB4. Demonstrating true competitive inhibition—and, by extension, a single binding site—would require more detailed transport kinetic analyses, such as those described in the studies cited above.
4. Conflation of findings from different PfCRT variants. The manuscript uses PfCRTDd2 for resistance selection experiments and PfCRTGB4 for molecular dynamics simulations and uptake assays, yet treats these variants as functionally equivalent. This is problematic, given their known differences in polymorphisms, transport kinetics, and inferred binding-site organization.
The intent of these comments is not to create undue difficulty for the authors, but rather to help prevent overstatements or inaccuracies that may undermine the manuscript at a later stage. I therefore recommend that the authors rework the cited paragraph in the Discussion to adopt a more nuanced interpretation, explicitly acknowledge the limitations arising from the use of PfCRT variants with distinct polymorphisms and transport properties, and avoid generalizing beyond what the data directly support. I am not requesting additional experimental work.

Reviewer #3

(Remarks to the Author)
The authors have thoroughly addressed all previous comments and suggestions.
I have no further remarks and would like to congratulate the authors on an excellent piece of work.

Open Access This Peer Review File is licensed under a Creative Commons Attribution 4.0 International License, which permits use, sharing, adaptation, distribution and reproduction in any medium or format, as long as you give appropriate credit to the original author(s) and the source, provide a link to the Creative Commons license, and indicate if changes were made.
In cases where reviewers are anonymous, credit should be given to 'Anonymous Referee' and the source.
The images or other third party material in this Peer Review File are included in the article's Creative Commons license, unless indicated otherwise in a credit line to the material. If material is not included in the article's Creative Commons license and your intended use is not permitted by statutory regulation or exceeds the permitted use, you will need to obtain permission directly from the copyright holder.
To view a copy of this license, visit https://creativecommons.org/licenses/by/4.0/

Okombo *et al.* Collateral hypersensitivity between ZY19489 and piperaquine neutralizes PfCRT-mediated drug efflux and *Plasmodium falciparum* resistance.

Manuscript# NCOMMS-25-76372, under consideration at *Nature Communications*.

RESPONSE TO REVIEWER COMMENTS

Note – page and line numbering relates to the PDF version of the resubmission with changes highlighted. New text is indicated below in blue, with in some instances the prior text indicated in red. New text is highlighted in yellow in the revised manuscript. A clean copy is also included as a Word document.

Part I: Summary

Reviewer #1: The authors have investigated the mechanism of resistance and the mode of action of the antimalarial clinical development candidate ZY19489. A comprehensive set of experiments was conducted, including in vivo selection of resistant parasites, whole-genome sequencing, QTL analysis of a genetic cross, and in vivo mutagenesis. These independent approaches all converge on PfCRT as the principal determinant of resistance. While the identification of PfCRT as a resistance determinant is not inherently surprising—given its known structural flexibility and poly-specific substrate recognition—what makes this study noteworthy is the discovery that the PfCRT variant conferring resistance to ZY19489 loses the ability to transport chloroquine (CQ) and piperaquine (PPQ). This finding of inverse susceptibility is of considerable interest, as it raises the possibility of an evolutionary trap, in which the use of two drugs with opposing PfCRT susceptibilities could limit the evolution of cross-resistance.

Reviewer #2: Okombo et al describe the characterisation of the mode of resistance and the mode of action of ZY19489, a new antimalarial compound currently in phase II clinical trials. By generating resistant parasites in vitro they discover a novel point mutation N246H in the well-studied PfCRT protein. They convincingly show by multiple approaches including the generation of genome edited parasite lines in different genomic backgrounds that this mutation confers low-level resistance shifting the IC50 by about 12-fold. QTL analysis confirms that PfCRT mutations can modulate ZY19489 activity. No resistance was obtained upon infection and treatment of humanised mice. Based on the available cryo-EM structure they model the consequence of the N246H mutation on PfCRT and the binding of ZY19489. Most interestingly, they observe that the N246H mutation confers sensitivity to aminoquinoline antimalarials and show that ZY19489 competes with chloroquine and piperaquine in CRT drug efflux. The N246H mutation results in a severe growth phenotype and ZY19489 retains potency against many of the crt and mdr1 alleles present in the field, features that are very much in favour for a new potential antimalarial compound and opens interesting perspectives of potential drug combinations with counterbalancing effects of resistance selection.

The authors address the mode of action of ZY19489 through metabolomic analysis of treated parasites, identify several pathways and focus on Hb catabolism, given the resistance mechanism conferred by CRT. The results are complex. No direct action on candidate proteins like PfHDP or falcipain 2A or 2B could be detected. Hb peptides are increased but ZY19489 seems not to block beta-hematin formation nor increase free heme levels while there is some augmentation in Hb levels. The authors suggest a possible additional mode of action. This is an excellently performed, controlled, described and presented study. Important new insights into PfCRT activity in resistance to different antimalarial compounds are identified and validated in a large number of genetic backgrounds confirming the complexity of genetic resistance to certain antimalarials and to ZY19489.

Reviewer #3: The manuscript investigates the mechanism of action and resistance profile of the novel, long-acting antimalarial ZY19489. Using a combination of growth inhibition assays, metabolomics, heme-fractionation, microscopy, and genetic approaches, the authors show that ZY19489 rapidly kills Plasmodium falciparum asexual stages and affects hemoglobin catabolism in the digestive vacuole rather than directly inhibiting heme detoxification or folate/pyrimidine pathways.

In vitro resistance selection (particularly in a hypermutable Dd2-Polδ background) identifies a key mutation in the chloroquine resistance transporter PfCRT (N246H) that confers reduced susceptibility to ZY19489. This mutation, however, comes with a substantial fitness cost and creates strong collateral hypersensitivity to piperaquine and related aminoquinolines. Proteoliposome transport assays support a model in which ZY19489 is a PfCRT substrate and N246H alters its transport properties. In vivo experiments in humanized NSG mouse model show robust efficacy of ZY19489 and suggest a relatively high barrier to resistance under the dosing regimens tested. Based on the collateral sensitivity profile, the authors propose that pairing ZY19489 with piperaquine could act as an “evolutionary trap” and form a non-ACT, resistance-refractory combination therapy.

The link from PfCRT-mediated resistance to ZY19489, through to collateral hypersensitivity to piperaquine and other aminoquinolines, is logically developed and supported by both genetic and transport data. This provides a coherent mechanistic explanation and a plausible rationale for combination design. This work directly addresses two critical issues for antimalarial development: (i) mechanism of action of a promising clinical candidate, and (ii) how resistance is likely to emerge and how it might be constrained by rational partner drug choice. The in vivo efficacy and resistance-selection data in humanized mouse models enhance the translational impact.

While the data indicate perturbation of hemoglobin uptake/catabolism and implicate PfCRT in resistance, the direct molecular target(s) of ZY19489 remain undefined. Some key mechanistic conclusions rely on correlative metabolomic and heme-fractionation patterns rather than direct measurements of endocytic or proteolytic steps.

The principal resistance mutation (N246H) arises in a hypermutator line under strong selection and carries a large fitness cost and pronounced collateral sensitivity. It is not yet clear how likely such a pathway would be to emerge and spread in natural parasite populations, and no epidemiological data are available.

The proposal to use ZY19489 with piperaquine as a resistance-refractory combination is mostly based on in vitro susceptibility and transport data. Dedicated in vivo combination and resistance-selection experiments for this specific pair are limited or absent, so the “trap” remains somewhat theoretical at this stage. However, given its rigorous multidisciplinary experimentation, clear mechanistic and evolutionary insights into a clinically relevant antimalarial candidate, and strong translational implications for rational, resistance-limiting combination therapy design, I endorse this manuscript for publication in Nature Communications pending satisfactory revision.

Reviewer #1

Comment #1. Quality of the structural models (**Figure 2).** Additional information is required to assess the robustness of the molecular models presented in **Figure 2**. Specifically, please include: i) graphical representations of RMSD for both the protein backbone and the ligand across all simulated conformations; ii) the docking scores for each pose; iii) solvent-accessible surface area (SASA) values; and iv) plots showing the distances between the ligand and key interacting residues as a function of simulation time.

Reply: We thank the reviewer for these suggestions. All analyses used to evaluate model robustness were performed during our molecular dynamics (MD) simulations. These data have now been added to the revised version of the Supplementary Information file (please refer to **Supplementary Table 9** and **Supplementary Fig. 6**). These include a table with ChemPLP docking scores and solvent-accessible surface area values. Additionally, we have included figures showing the time evolution of ligand stability within the binding cavity, residue-level flexibility profiles of the protein, and the distances between ZY19489 and the key interacting residues. These results confirm that the modeled conformations remained stable throughout the trajectories and that the ligand maintains a consistent binding mode across replicates.

Comment #2. Clarification regarding hydrogen bond persistence (**Figure 2**). The figure legend states that “a significant hydrogen bond persisted for more than 30% of the simulation time.” This

implies that the interaction was absent for approximately two-thirds of the trajectory. Please clarify the interpretation: does this mean the hydrogen bond is transient and thus not functionally significant?

Reply: We thank the reviewer for highlighting this point. The interaction with the Q161 residue in the N246H mutant demonstrated approximately 30% hydrogen-bond occupancy in only one of the three independent MD replicates. This interaction was not detected in the remaining two replicates.

Hydrogen-bond persistence values in the range of 20 to 50% are widely considered indicative of meaningful but dynamic stabilization in MD simulations, particularly in protein–ligand systems where conformational fluctuations and solvent competition naturally weaken average occupancy. These medium-occupancy hydrogen bonds contribute intermittently but repeatedly to ligand residence energetics and might play a role in the overall binding thermodynamics and recognition process. However, while a ~30% occupancy supports the notion that this interaction is possible and energetically favorable under certain conformational states, it was only observed in one of the three independent trajectories of the 7G8+N246H isoform. The absence of the hydrogen bond in the other two replicates suggests that this contact is not consistently sustained in the mutated protein, which is in line with our mechanistic interpretation. Our modeling suggests that the N246H mutation changes the environment of the open-to-cytosol cavity, increasing solvent exposure and disrupting the stabilizing interactions observed in the parental 7G8 transporter. As a result, ZY19489 forms fewer and less persistent molecular contacts in the 7G8+N246H mutant, leading to weaker binding stabilization and facilitating efflux, which aligns with both our putative structural observations and the functional data indicating resistance. To prevent misunderstanding, we have revised the figure caption and main text. The revised sentence now reads as follows:

Results (page 8, lines 187 -190): "In the 7G8+N246H variant, we observed only one hydrogen bond interaction with Q161 that persisted for more than 30% of the simulation period. This hydrogen bond was not consistently detected in all trajectories; appearing exclusively in one of the three independent molecular dynamics replicate simulations."

The revised **Figure legend** now reads as follow:
"**Figure 2** (starting page 42 line 1139): **B** Hydrogen bonding patterns between ZY19489 and 7G8 PfCRT in the open-to-cytosol conformation implicate the side chain of E232 and the backbone carbonyl group of L245 as important for stabilizing drug-transporter interactions. Bonding pattern with 7G8+N246H PfCRT, illustrating a single significant hydrogen bond between the carbonyl oxygen of the Q161 side chain and the ligand that persisted more than 30% of the simulation in one of the three molecular dynamics simulations replicates. Hydrogen bonds are represented as dashed blue lines."

Comment #3. Molecular dynamics simulations for ZY19489 versus CQ and PPQ. To better understand the mechanistic basis of the observed inverse susceptibility, additional MD simulations should be performed comparing ZY19489, CQ, and PPQ. Do these ligands occupy distinct regions of the substrate-binding cavity, or are their binding poses overlapping? Is there evidence of steric hindrance or mutual exclusion between them?

Reply: The available structural and computational data allow us to explore the relationship between ZY19489, CQ, and PPQ within the PfCRT transport cavity. Kim *et al.*, 2019 (reference #16) used cryo-EM and MD studies of chloroquine (CQ) bound to wildtype (WT) PfCRT to propose that this drug occupies the central substrate-binding cavity, interacting with residues surrounding the canonical resistance locus, including residues 72, 75, 76, and 97. In our simulations, ZY19489 consistently sampled the same T76-adjacent region of the cavity in the 7G8 protein, indicating that ZY19489 and CQ engage largely overlapping binding volumes rather than distinct or spatially separated pockets.

Given the structural similarities between CQ and piperaquine (PPQ), and prior models of PPQ interaction with PfCRT, PPQ is also expected to occupy the same central cavity. In line with this, the results collected in the proteoliposome assays support this interpretation. ZY19489 was observed to

strongly inhibit the transport of radiolabeled CQ and PPQ, and reciprocally CQ and PPQ inhibited ZY19489 transport. This pattern indicates mutual exclusion, which in the context of PfCRT does not imply steric hindrance but rather the intrinsic single-substrate nature of the transporter, in which only one ligand can occupy the central cavity at a time. Any ligand already bound to that region would prevent binding or transport of another. The competition pattern observed experimentally is therefore consistent with all three compounds accessing the same substrate-binding cavity. The published structural data for CQ, the expected PPQ cavity engagement based on structural similarity, and our simulations of ZY19489 in WT PfCRT combine to indicate that the three ligands interact with a shared central binding region. We have included parts of this response into the **Discussion** to help readers to better understand the mechanistic basis of basis of the observed inverse susceptibility and the occupancy of ZY19489 in the cavity.

Discussion (starting page 17, line 437): "Curiously, parasites expressing the N246H mutation were sensitized to DV-acting antimalarials including PPQ and CQ. In our simulations, ZY19489 consistently samples the same T76-adjacent region of the cavity, indicating that this molecule, CQ and PPQ engage largely overlapping binding volumes rather than distinct or spatially separated pockets. This interpretation aligns with our proteoliposome data where transport of CQ and PPQ by PfCRT variants that mediate resistance to these drugs was potently inhibited in the presence of ZY19489. This pattern suggests mutual exclusion which, in the context of PfCRT, does not imply steric hindrance but rather the intrinsic single-substrate nature of the transporter in which only one ligand can occupy the central cavity at a time and preclude binding or transport of another. The competition pattern observed experimentally is therefore consistent with all three compounds accessing the same substrate-binding cavity."

Comment #4. Verification of metabolites in *P. falciparum* extracts. Please clarify whether the proposed metabolites were experimentally verified in the context of *P. falciparum* extracts. This is an important control, as many metabolites display altered migration or detection profiles due to matrix effects from parasite extracts.

Reply: We agree with the reviewer that this is an important query. The metabolites were confirmed by comparing their retention times with those of pure molecular standards run on the same mass spectrometer. Matrix effects are challenging to control for, but with replicative and reproducible experiments compared to our molecular standards and control compound effects on the parasite, we report reproducible metabolite measurements for comparative fingerprint profiling. We have added this confirmation in the **Materials and Methods** section which now reads as follows:

Materials and Methods (page 32, lines 816 - 817): "The metabolites were confirmed by comparing their retention times with those of pure molecular standards run on the same mass spectrometer."

Comment #5. Experimental testing of the evolutionary trap hypothesis. Have the authors attempted to test the evolutionary trap hypothesis experimentally—for example, by exposing parasites to combinations of ZY19489 with CQ or PPQ? Such data would significantly strengthen the manuscript's central claim.

Reply: The reviewer raises an important point, and we would be very keen to experimentally test this specific hypothesis. Theoretically, one could select for resistance to one compound tested alone and then run parallel selections with that compound plus a range of sublethal concentrations of the second compound to show that parasites could not evolve dual resistance. However, the rarity of selecting for resistance to any of these compounds makes this infeasible. For ZY19489, resistance selection required pressuring hypermutable Dd2-Polδ parasites with ZY19489 for five months of consecutive culture to obtain a mutant line from one of three flasks with 10^9 parasites each (corresponding to 100 to 200 mL of culture per flask). This line is about 10-fold more mutable then standard Dd2 as shown by Kumpornsin *et al., Nature Commun* 2023 (reference #12). Repeating this experiment would require liters of culture per day over that time period, combining ZY19489 with varying concentrations of PPQ or CQ. This would be required in order to show that instead of obtaining resistance in one (or perhaps two) flasks, we obtained no resistance in any (i.e. reducing the frequency from 1 or 2 to 0). This vast culturing effort would therefore not yield any statistically

robust effort. We also note that in the malaria literature there are only two reports where CQ or PPQ resistance have ever been generated, both with tailored situations where only a single mutation was required (Fidock *et al.*, *Molec Cell* 2000, PMID 11090624; Eastman *et al.*, *Antimicrob Agents Chemother*, 2011, PMID 21576453), adding to the complexity of this experiment. These combination experiments have only been possible with compounds (such as PfDHODH inhibitors) for which resistance is readily acquired (for example with numbers in the range of 10^6 to 10^7 parasites; Lukens *et al.*, 2014, *PNAS*, PMID: 24381157).

In response to the reviewer, we have toned down our discussion of this evolutionary trap as follows:
Abstract (page 2, lines 35 - 39):
From "Uptake studies in PfCRT-containing proteoliposomes provide evidence that ZY19489 can block mutant PfCRT-mediated efflux of piperaquine and chloroquine, ~~creating~~ a scenario of an evolutionary trap whereby resistance to ZY19489 ~~blocks PfCRT-mediated resistance and~~ restores susceptibility to piperaquine and chloroquine".
To "Uptake studies in proteoliposomes loaded with recombinant purified PfCRT provided evidence that ZY19489 can block transport of piperaquine and chloroquine mediated by drug-specific PfCRT variants. In parasites, these PfCRT variants are thought to mediate resistance by effluxing these drugs out of the digestive vacuole. Our findings evoke a scenario of an evolutionary trap whereby resistance to ZY19489 can block piperaquine and chloroquine efflux and thereby restore their activity.".

Discussion (page 18, lines 449 - 454):
From "~~In such a scenario, our data suggest that two drugs could theoretically constitute an effective combination that might force *pfcrt* into an "evolutionary trap" and prevent the emergence of high-grade resistance to either. This concept has been demonstrated for other antimalarials, including PfDHODH, Pf20S proteasome, and mitochondrial cytochrome bc_1 complex inhibitors[11]…~~"
To "In such a scenario, our data suggest that two drugs could theoretically constitute an effective combination that would force *pfcrt* into an "evolutionary trap" and prevent the emergence of high-grade resistance to either. This concept remains theoretical, as the rarity of selecting for resistance to these compounds alone or in combination precludes experimental validation. Nevertheless, this hypothesis has been validated for other antimalarials, including PfDHODH, Pf20S proteasome and mitochondrial cytochrome bc_1 complex inhibitors[10]…"

Comment #6. Discussion of literature on subverted inverse susceptibility. There are documented cases where inverse susceptibility was eventually overcome by cross-resistance, especially in proteins with high mutational plasticity such as PfCRT. The authors should acknowledge and discuss this possibility, citing relevant work (e.g., Mandt et al., eLife 2023, DOI: 10.7554/eLife.85023; Nichol et al., Nat Commun 2019, DOI: 10.1038/s41467-018-08098-6).

Reply: We agree with the reviewer on this point. We have subsequently modified the **Discussion** to acknowledge this and included the suggested references. The additional sentences we have included in the **Discussion** are as follows:

Discussion (starting page 18, line 460):
From "~~This is instructive to the ongoing Phase II trials in Gabon where mutant *pfcrt*, which could provide a potential background for N246H to emerge, constituted an estimated 53% of all samples in a recent report[45]. Nonetheless, the high resistance barrier of ZY19489 and steep fitness cost associated with PfCRT N246H suggest that clinical resistance to this antimalarial is unlikely to arise as mutant parasites would likely be rapidly outcompeted in the field.~~"
To "This is instructive to the ongoing Phase Ib trials in Gabon where mutant *pfcrt*, which could provide a potential background for N246H to emerge, constituted an estimated 53% of all samples in a recent report[44]. It is important to point out that this inverse susceptibility phenomenon could still be overcome by cross-resistance, especially in proteins with high mutational plasticity like PfCRT and PfDHODH. For instance, *in vitro* selection experiments with DSM265 in combination with TCMDC-125334 (active against all PfDHODH mutant lines) yielded parasites with a PfDHODH V532A mutation that were cross-resistant to both compounds and as fit as the wildtype parent[45]. Additionally, a mathematical quantification and experimental evolution study in *Escherichia coli* highlighted that a

second drug can stochastically select for increased susceptibility or increased resistance when following a first[46]. Nonetheless, the absence of overt resistance under the ZY19489-containing regimens tested and the steep fitness cost associated with PfCRT N246H suggest that clinical resistance to this antimalarial is unlikely to arise as mutant parasites would likely be rapidly outcompeted in the field. We, however, urge caution in this interpretation since we acknowledge that longer selection or different dosing might still identify additional resistance pathways. We note that this mutation has not been observed in any of the ~20,000 genomes present in the MalariaGEN Pf7 data."

Overall Assessment
This is a well-designed and timely study that contributes valuable insights into the mechanisms of antimalarial resistance and the structural dynamics of PfCRT. The concept of an evolutionary trap mediated by inverse susceptibility is particularly intriguing and potentially impactful. Addressing the points outlined above—especially those concerning the molecular modeling data and experimental verification of key hypotheses—would substantially strengthen the manuscript and broaden its relevance.

Reply: We thank the reviewer for this positive assessment

Reviewer #2:

Comment #1. - P4 line 91: please give a value of the previously observed low-level resistance.

Reply: We have deleted this line. In its place, we have now provided more context in the Introduction section and quantified the level of resistance referred to by this phrase. The revised lines now read as follows:
Introduction (starting page 3, line 69):
From "~~In a previous report, low-level resistance to this molecule was associated with a G29V mutation in subunit D of the P. falciparum vacuolar proton-transporting V-type ATPase complex…~~"
To "In prior *in vitro* selection experiments using several triaminopyrimidines structurally analogous to ZY19489, one derivative (compound **6**; Fig. 1a) yielded resistant parasites that exhibited a 3-fold increase in the half-maximal growth inhibition concentration (IC_{50}) compared to parental Dd2[7]. This low-level resistance was associated with a G29V mutation in..."

We have also included the chemical structure of compound **6** in **Figure 1A**.

Results (page 4, lines 91 - 92):
From "~~Applying a ramping in vitro selection method to P. falciparum Dd2 parasites, a previous study observed low-grade resistance after ~200 days of drug pressure[7]. Whole-genome sequence (WGS) analysis associated resistance with a G29V mutation in PfV1-D (subunit D of the *P. falciparum* vacuolar proton-transporting V-type ATPase complex). To test whether this association was causal, we generated…~~"
To "To test whether the prior association between ZY19489 resistance and a mutated form of the *P. falciparum* V-type ATPase complex[7] was causal (see Introduction), we generated a..."

Comment #2. - P5 line 108: indicate how many clones were generated and sequenced.

Reply: We have now indicated the number of clones that were generated, phenotype and sent out for whole genome sequencing. This information is in the revised sentences that now read as follow:
Results (page 5, lines 104 -105):
From "~~Resistance was confirmed in parasites cloned by limiting dilution (Fig. 1b; Supplementary Table 3)"~~
To "Resistance was confirmed in four parasites cloned by limiting dilution (**Fig. 1b; Supplementary Table 3**).

Results (page 5, line 107):
From "~~WGS analysis of the ZY19489-pressured clones identified single nucleotide…~~"
To "Whole-genome sequence (WGS) analysis of all the four ZY19489-pressured clones identified single nucleotide..."

Comment #3. P8 line 180ff: The conclusions here might need some more explanation. How can an occluded form facilitate drug transport? Is there a possibility that ZY19489 could enter the DV through CRT? Is the open-to-cytosol conformation just another term for closed-to-DV? If the mutation reduces ZY19489 interactions close to the cytosol side, do the authors conclude that ligand release into the cytosol is facilitated and would be the reason for resistance through faster transport?

Reply: We have now expanded on our interpretation as follows:

Results (page 8, lines 179 -182):
From: "~~This salt bridge formation alludes to a tendency for the mutant protein to adopt a closed-to-DV (occluded) conformation and suggests that the mutation induces resistance to ZY19489 by favoring this closed state that may facilitate drug transport. Conversely, the 7G8 protein does not adopt this DV-closed conformation, potentially resulting in reduced transport activity (Supplementary Fig. 5)."~~
To: "This salt bridge formation suggests that the mutant protein might transiently adopt a occluded (partially closed-to-DV) conformation and that the N246H mutation might confer resistance to ZY19489 by favoring this occluded state. Conversely, the 7G8 protein did not appear to adopt this occluded conformation (Supplementary Fig. 5; Supplementary Fig. 6)."

Discussion (starting page 17, lines 438): "In our simulations, ZY19489 consistently samples the same T76-adjacent region of the cavity, indicating that this molecule, CQ and PPQ engage largely overlapping binding volumes rather than distinct or spatially separated pockets. This interpretation aligns with our proteoliposome data where transport of CQ and PPQ by PfCRT variants that mediate resistance to these drugs was potently inhibited in the presence of ZY19489. This pattern suggests mutual exclusion which, in the context of PfCRT, does not imply steric hindrance but rather the intrinsic single-substrate nature of the transporter in which only one ligand can occupy the central cavity at a time and preclude binding or transport of another. The competition pattern observed experimentally is therefore consistent with all three compounds accessing the same substrate-binding cavity"

Comment #4. P10 Fig 3A: is this a bloated FV phenotype indicative of reduced Hb degradation?

Reply: From previous evidence of PfCRT mutations and the bloated digestive vacuole phenotype, we are confident that this morphological feature is directly linked to impaired ability of the parasite to optimally degrade host hemoglobin. We have now added a few lines in the **Discussion** section to highlight the possible link between this phenotype, PfCRT mutations and poor multiplication rate in the mutant parasites.

Discussion (page 19, lines 478 - 490):
From "~~Further, aberrant accumulation of peptides has been observed in variant PfCRT isoforms where it is associated with reduced parasite fitness, presumably from deprivation of essential globin-derived amino acids[28,43,44]. Our analysis identified HVDD (a truncated version of HVDDM) and VDPVNF, both of which were demonstrated to be putative PfCRT substrates in [^{3}H]-CQ *cis*-inhibition experiments in Xenopus laevis oocytes[45]~~
To "Further, our results are consistent with an important role of PfCRT mutations in dictating levels of short oligopeptides in parasites. Aberrant accumulation of peptides has been observed in variant PfCRT isoforms and is predicted to impact the pool of essential amino acids available for protein synthesis, osmotic integrity, and numbers of merozoites that can develop within the confines of the infected RBCs[29,47,48]. This suboptimal physiological context agrees with the lower parasite multiplication rate and reduced parasite fitness observed in the PfCRT N246H mutant in our study and the PPQ resistance-conferring F145I variant earlier studied[49,50]. The anomalous peptide accumulation in the N246H mutant also suggests that one of the native roles of PfCRT involves

shuttling of peptides from the DV lumen to the cytosol for subsequent liberation of amino acids and alleviation of osmotic stress from the DV, with this function being impaired in drug-resistant PfCRT mutants. This interpretation is supported by a study of PfCRT isoforms expressed in *Xenopus laevis* oocytes that demonstrated PfCRT-mediated transport of peptides ranging from 4 to 11 amino acids, with the 3D7 isoform (CQ-sensitive) permitting a greater quantity and broader range of peptides than the Dd2 (CQ-resistant) isoform[51]."

Comment #5. P10 line 245: has the N246H mutation been found in the 10,000 Pf field genomes?

Reply: No, it has not. We searched for this mutation, without success, in the Pf7 repository which contains *P. falciparum* genome variation in 20,000 worldwide samples collated from the MalariaGEN Pf3k database, *Plasmodium falciparum* Community Project and GenRe Mekong Project. This is now mentioned in the **Discussion** as follows:
Discussion (page 19, lines 472 - 473): "We note that this mutation has not been observed in any of the ~20,000 genomes present in the MalariaGEN Pf7 data."

Comment #6. P11 in vivo experiments: There is no method section for these experiments. How many mice were treated? How many showed recrudescence? Which combinations at which concentrations were done? Perhaps a supp figure similar to S2 for the in vitro selection could help. What is the 3D7-0087/N9 strain? Why was 3D7 used here?

Reply: We have now provided the method used to generate the *in vivo* data. This information is now available in the **Materials and Methods** section (starting page 29, line 754) under the title "***In vivo* efficacy studies in the TADhuMouse®-Pfalc model**". We agree with the reviewer that a figure would help assist readers follow this part of the manuscript. We have therefore included a supplementary file (**Supplementary Fig. 7**) to show the number of mice that were treated in each category and the number that showed recrudescence. Additionally, information on the doses used in each combination is also included in **Table 2** in the main text as well as the new **Supplementary Fig. 7**. We further describe in detail the $3D7^{0087/N9}$ strain as a standard *P. falciparum* strain adapted to grow reproducibly in humanized NOD-*scid* $IL\text{-}2R\gamma^{null}$ (NSG) mice. This description is reflected in the **Results** section, which reads as follows:

Results (page 11, lines 275 - 278): "To assess whether ZY19489-resistant parasites might emerge *in vivo* following combination treatment, we used a validated mouse model of *P. falciparum* ABS infection of NOD-*scid* $IL\text{-}2R\gamma^{null}$ (NSG) mouse engrafted with human erythrocytes (TADhuMouse®)[22]."

Comment #7. P13 stage-dependent activity. I acknowledge the courage to present the observed relatively lower susceptibility of trophozoites to the drug. This observation is somewhat in contradiction to the predicted mode of action of ZY19489 through an effect on Hb degradation or possibly thymidine production that should be predominant in trophozoites. At the current state of knowledge this might be difficult to explain but these conflicting observations should as such briefly been mentioned in the discussion, P18 line 460.

Reply: We thank the reviewer for this comment and appreciate the possible contradiction in interpreting the mode of action of the molecule. While we agree that Hb degradation peaks in the trophozoite phase, we nonetheless argue that the mode of action of this compound is likely to be in the very early phase of hemoglobin degradation before this process is optimal in trophozoites. Indeed, supported by identification of active hemoglobinases (falcipains 2 and 3) at this stage, Xie *et al.*, 2016 (reference #53) demonstrated that hemoglobin uptake and digestion begins and is active in early rings. Additionally, fluorescence and electron tomography studies have shown that, by the mid-ring stage, parasites have detectable acidified compartments that contain small hemozoin crystals. It is this presence of a hemoglobin degradation machinery in the rings that we presume renders this stage of the parasite susceptible to ZY19489. Furthermore, our cell fractionation data shows a slight concentration-dependent increase in hemoglobin heme but not 'free' heme or hemozoin, consistent with hemoglobin heme species being a product of early degradation of host

hemoglobin. We have included parts of this reasoning in the **Discussion** section, which now reads as follows:

Discussion (page 20, lines 495 - 508):
From "~~The absence of direct interaction between ZY19489 and 'free' heme, or the putative Hz nucleation factor PfHDP, or the hemoglobinases falcipains 2A and 2B, suggests inhibition of other targets within the Hb catabolism pathway. Our metabolomic analysis also revealed an accumulation of deoxyuridine monophosphate, which could imply a potential impact on pyrimidine biosynthesis. This likelihood of an additional MoA could help explain the compound's high barrier for resistance. Moreover, Hb uptake and degradation begins in early rings[46] and pyrimidine biosynthesis peaks during the trophozoite to schizont transition[47], which would align with our observations of stage-specific potency against rings and schizonts.~~"
To "The ZY19489 potency observed against rings and schizonts in the stage-specificity experiments is informative. Fluorescence and electron tomography studies have shown that, by the mid-ring stage, parasites have detectable acidified compartments that contain small Hz crystals[52]. Additionally, Hb uptake and degradation has been shown to begin in early rings[53]. The presence of this active Hb degradation machinery in rings may render this stage highly susceptible to ZY19489. On the other hand, pyrimidine biosynthesis peaks during the trophozoite to schizont transition[54]. Our metabolomic analysis also revealed an accumulation of deoxyuridine monophosphate, implying a potential impact on pyrimidine biosynthesis and corroborating the observed stage-specific potency against schizonts. This likelihood of an additional MoA could help explain the compound's high barrier for resistance in the conditions tested. Our combined molecular dynamics simulations, heme fractionation and metabolomics studies lead us to propose that ZY19489 might act primarily in the parasite cytosol, and that the N246H mutation might enable mutant PfCRT to sequester this compound in the DV, away from its cytosolic site of action. In contrast, evidence suggests that CQ and PPQ act primarily in the DV by inhibiting Hz formation, with mutant PfCRT mediating resistance by effluxing these drugs out of the DV away from their heme target[17]."

Comment #8. P21 resistance selection: please refer to **Fig S2.**

Reply: To guide readers to where more information on how resistance selection with ZY19489 was performed, we have added the words "**Supplementary Fig. 2**" to the first line of the **Materials and Methods** section under which resistance selection methodology falls. This section now reads as follows:
Materials and Methods (page 23, lines 574 - 575): "*In vitro* selection was conducted using a pulsing (intermittent drug pressure) method on Dd2-Polδ (Supplementary Fig. 2)."

Comment #9. P21 line 544: how many clones?

Reply: We have now indicated the number of clones that were obtained from limiting dilution for IC_{50} determination and subsequent whole genome sequencing. The new sentence now reads as follows:
Results (page 5, lines 104 -105):
From "~~Resistance was confirmed in parasites cloned by limiting dilution (Fig. 1b; Supplementary Table 3)"~~
To "Resistance was confirmed in four parasites cloned by limiting dilution (**Fig. 1b; Supplementary Table 3**).

Results (page 5, line 107):
From "~~WGS analysis of the ZY19489-pressured clones identified single nucleotide…~~"
To "Whole-genome sequence (WGS) analysis of the four ZY19489-pressured clones identified single nucleotide..."

Materials and Methods (page 23, lines 590 - 591):
From "~~Clones were obtained from the resistant bulk cultures by limiting dilution.~~"
To "Four clones were obtained from the resistant bulk cultures by limiting dilution."

Comment #10. P27 line 694: how many hours between Percoll and sorbitol treatment?

Reply: We have now indicated the length of time between the two synchronization treatments. The new sentence now reads as follows:

Materials and Methods (page 29, lines 738 - 740):
From "~~Briefly, mature schizonts were obtained via Percoll gradient purification. Upon reinvasion of RBCs, cultures were synchronized by sorbitol treatment to obtain pure early rings.~~"
To "Briefly, highly synchronized early ring-stage parasites were obtained via 70% Percoll gradient purification of mature schizonts followed 5 hours later by sorbitol treatment of young rings upon reinvasion of RBCs."

Comment #11. P41 figure legend 4D: it would help understanding to indicate in the legend that rings were treated for 30h with drugs before analysis.

Reply: We have now added this information to the **Figure legend**. This revised section now reads as follows:
Figure 4 (page 44, lines 1179 - 1180):
From "~~Heme fractionation of CQ- and ZY19489-treated NF54 parasites with different drug concentrations.~~"
To "Heme fractionation of synchronized NF54 parasites treated for 30 hours with different drug concentrations of ZY19489 or CQ."

Comment #12. Fig 4: I suggest to use the same scale (5-9 fg/cell) for free heme in panel 4H than in 4E to facilitate comparison. In 4H green data points could be connected by a dotted line for visibility.

Reply: We thank the reviewer for this suggestion. We have now ensured that the chloroquine (CQ) and ZY19489 plots in this figure are draw to comparative scales in all axes. To be specific, **Figure 4D** and **4G, 4E** and **4H** as well as **4F** and **4I** now use the same scales, respectively. We have also connected the green data points on **Figure 4H** for better visibility and to assist readers see that the 'free' heme levels do not track with parasite death/survival in ZY19489-treated parasites.

Comment #13. Supp Fig S1: panel B: how many hours after aTc? The decrease in GAPDH and HA might actually be similar. The loading control actually shows that the experiment didn't work, no parasite material in the -aTc sample.

Reply: We thank the reviewer for this comment. Knockdown of V1-D exerts a strong inhibitory effect on the parasite growth, as evidenced by clearance following removal of aTc from the culture medium. To ensure consistency with the drug assay knockdown conditions, we repeated the Western blot using a low aTc concentration (6 nM). These blots were prepared from cell lysates collected 72 hours after aTc washout. We have now included in **Supplementary Fig. S1** the repeated image of the Western blot.

Comment #14. Supplementary Fig. S3: for curiosity, N246H is located in which crt exon?

Reply: Codon 246 is located in exon 5 of *pfcrt*. We have now added this information on the sentence where the N246H mutation is first-ever mentioned in the manuscript. The new sentence now reads as indicated below:
Results (page 5, lines 108 - 110):
From "~~Of these, the N246H mutation in *pfcrt* and D233N in the anaphase-promoting complex subunit 10 (*pfapc10*; PF3D7_1217600) were present in all clones.~~"
To "Of these, the N246H mutation in exon 5 of *pfcrt* and D233N in the anaphase-promoting complex subunit 10 (*pfapc10*; PF3D7_1217600) were present in all the clones."

Reviewer #3:

Comment #1. The metabolomics data and heme-fractionation experiments are interpreted as showing that ZY19489 interferes with Hb endocytosis/catabolism rather than heme detoxification. However, the evidence is still somewhat indirect:
a) The decrease in short Hb-derived peptides after 2.5 h of treatment could reflect reduced peptide production, altered peptide export, or broader DV dysfunction, not necessarily a specific upstream step in Hb uptake or digestion.
b) The heme fractionation experiments show increased Hb and relatively unchanged "free" heme and hemozoin at relevant concentrations, but this pattern could also arise from changes in growth rate or DV morphology rather than a primary MoA.
c) Knockdown/KO of PfHDP and falcipains 2A/2B rules out those components, but the identity of the primary molecular target remains undefined.
Can the authors provide more direct experimental evidence that ZY19489's primary antiparasitic activity is mediated through disruption of hemoglobin uptake/catabolism (e.g., live imaging of Hb endocytosis, measurement of DV endocytic flux, or genetic/chemical epistasis with known Hb-pathway mutants), or else temper the mechanistic claim accordingly?

Reply: The reviewer raises very valid points. Indeed, it has proven experimentally extremely difficult to identify the precise MoA of a number of antimalarials that show an effect on Hb catabolism yet do not show a profile of substantial hemozoin inhibition, as exemplified most notably by lumefantrine (Combrinck *et al.*, 2013, *ACS Chem Biol*; PMID: 23043646). The metaprint pattern obtained here with ZY19489 shows a clear effect on peptides, consistent with the earlier report from the Llinas lab showing perturbation of Hb catabolism to be a signature of hemozoin inhibitors (Allman *et al.*, 2016, reference #30). We have been unable to generate more direct evidence that ZY19489's primary MoA is via disruption of Hb catabolism. Instead, as suggested by the Reviewer, we have tempered our mechanistic claims as follows:

Abstract (page 2 lines 39 - 40):
From "Metabolomic studies ~~revealed~~ that ZY19489 significantly reduces intracellular levels of short hemoglobin-derived peptides"
To "Metabolomic studies provide evidence that ZY19489 significantly reduces intracellular levels of short hemoglobin-derived peptides…"

Results (page 14 line 335):
From "~~ZY19489 inhibits parasite growth by interfering with hemoglobin catabolism~~"
To "ZY19489 inhibition of parasite growth is associated with hemoglobin catabolism inhibition"

Results (page 14 line 356):
From "ZY19489 inhibits hemoglobin breakdown but not heme detoxification"
To "Evidence that ZY19489 inhibits hemoglobin breakdown but not heme detoxification"

Comment #2. The manuscript proposes PfCRT N246H as a key resistance determinant but also notes a 60–75% asexual blood-stage fitness cost and strong collateral sensitivity to multiple aminoquinolines:
a) N246H was selected in a hypermutable Dd2-Polδ line under prolonged high-dose pressure, which may not reflect natural selection conditions.
b) There is no current field surveillance evidence for N246H or closely related mutations in clinical isolates.
c) The claim that such mutants would be "rapidly outcompeted" in the field is plausible but not quantitatively supported.
Can the authors provide additional data or analysis addressing the in vivo relevance of PfCRT N246H (e.g., competition assays at lower starting frequencies, modeling of within-host or between-host dynamics, or a deeper scan of existing genomic datasets for N246H or compensatory mutations)? If not, the discussion could better distinguish between experimentally observed resistance pathways and their likely epidemiological probability.

Reply: We completely agree with the reviewer that owing to the high fitness constraint this mutation confers, parasites bearing this allele are unlikely to occur in the field and certainly not at high frequency. We however take note of the fact that ZY19489+ferroquine trials are still ongoing and our recommendation would be that in the event of any recrudescence from treatment failure then *pfcrt* should be among the candidate genes to be sequenced and codon 246 would be of particular interest. We nonetheless acknowledge that this point can be better argued in the **Discussion** section in a manner that distinguishes between experimentally observed resistance pathways and their likely epidemiological probability. We have therefore revised part of our **Discussion** as follows:

Discussion (page 19, line 468 – 473): "Nonetheless, the absence of overt resistance under the ZY19489-containing regimens tested and the steep fitness cost associated with PfCRT N246H suggest that clinical resistance to this antimalarial is unlikely to arise as mutant parasites would likely be rapidly outcompeted in the field. We, however, urge caution in this interpretation since we acknowledge that longer selection or different dosing might still identify additional resistance pathways. We note that this mutation has not been observed in any of the ~20,000 genomes present in the MalariaGEN Pf7 data."

Comment #3. The manuscript suggests that ZY19489 + PPQ could constitute an "evolutionary trap" and a resistance-refractory non-ACT combination, based mainly on:
a) In vitro collateral hypersensitivity of PfCRT N246H mutants to CQ/PPQ, and
b) Proteoliposome transport data showing ZY19489 inhibition of mutant PfCRT-mediated CQ/PPQ efflux.
However:
a) There are no in vivo combination efficacy or resistance-selection experiments specifically with ZY19489 + PPQ (only ferroquine, MMV693183, and M5717).
b) Potential safety concerns and known PPQ-associated resistance and cardiotoxicity issues are not discussed in detail.
c) It is not shown that long-term co-exposure cannot select other pfcrt or pfmdr1 variants that might erode this "trap".
Given that no in vivo data are presented for ZY19489 + PPQ, can the authors either (i) provide preliminary combination data (e.g., mouse efficacy or resistance-selection experiments, PK/PD compatibility) or (ii) clearly frame the ZY19489–PPQ combination as a theoretical proposal based on in vitro transport and susceptibility data, rather than a fully supported resistance-refractory strategy?

Reply: Our manuscript already provides data on ZY19489 combined with three other antimalarials already undergoing evaluation in human clinical trials, notably MMV693183 (an acetyl CoA synthethase inhibitor), ferroquine (used in the partner drug Phase Ib combination with ZY19489, and M5717 (**Table 2**). These studies showed no gain of resistance to ZY19489. These studies were conducted in the humanized RBC mouse model of P. falciparum infection by The Art of Discovery in Bilbao, Spain, financed by MMV. These are extremely expensive studies that we cannot directly fund. Instead, as proposed by the Reviewer, we have now toned down our text to frame this "evolutionary trap" as a theoretical proposal. Our changes are described above in our response to **Reviewer #1 Comment #3**.

Minor points
Comment #*4*. In Table 1 the compound name appears as "ZY91489" rather than "ZY19489", which looks like a typographical error.

Reply: We have now corrected this. The table title now reads as follows (page 45, line 1189):
"Table 1. Mutations identified from whole-genome sequencing of ZY19489-selected Dd2-Polδ clones."

Comment #5. For several key datasets (metabolomics, heme fractionation, stage-specific activity, MD analyses), the exact number of biological replicates and the multiple-testing strategy are not always fully explicit in the main text.

Reply: We have now indicated the number of independent biological repeats conducted for 72-hour IC_{50} testing, heme fractionation, stage specificity assay and metabolomic analysis in the **Materials and Methods** sections. We have also ensured this information appears in the Figure legends where plots relating to these data are depicted. In addition, we have included the statistical tests used in the analyses for these data. In some instances in the main text, this information is provided in parentheses. For each molecular dynamics simulation, we have now also indicated the number of replicates done in the figure legend of the new Supplementary Fig. 6.

Comment #6. Consider softening causal language (e.g., "suggests" rather than "demonstrates") where the link between metabolite pattern and mechanism is primarily correlative.

Reply: We have toned down on this causal language in relation to metabolite/peptide pattern. The one sentence where this strong deduction was made has been tempered down and now reads as follows:
Discussion (page 19, lines 490 - 491):
From "~~Our analysis identified HVDD (a truncated version of HVDDM) and VDPVNF, both of which were demonstrated to be putative PfCRT substrates in [^{3}H]-CQ *cis*-inhibition...~~"
To "Our analysis identified HVDD (a truncated version of HVDDM) and VDPVNF, both of which were suggested to be putative PfCRT substrates in [^{3}H]-CQ *cis*-inhibition..."

Comment #7. The claim of a "high in vivo barrier to resistance" is largely based on the absence of ZY19489-specific mutations in a limited number of recrudescent lines and combinations. It might be more precise to phrase this as "no overt resistance observed under the regimens tested" and acknowledge that longer selection or different dosing might still identify additional resistance pathways.

Reply: We agree with the reviewer on this point. We have therefore rephrased part of the Discussion to reflect this cautious interpretation. The modified segment of the **Discussion** now reads as follows:

Discussion (page 19, lines 468 - 472):
From "~~Nonetheless, the high resistance barrier of ZY19489 and the steep fitness cost associated with PfCRT N246H suggest that clinical resistance to this antimalarial is unlikely to arise as mutant parasites would likely be rapidly outcompeted in the field.~~"
To "Nonetheless, the absence of overt resistance under the ZY19489-containing regimens tested and the steep fitness cost associated with PfCRT N246H suggest that clinical resistance to this antimalarial is unlikely to arise as mutant parasites would likely be rapidly outcompeted in the field. We, however, urge caution in this interpretation since we acknowledge that longer selection or different dosing might still identify additional resistance pathways."

Comment #8. Ensure that all axes are clearly labelled with units (e.g., nM vs μM; fg/cell) and that symbols/colors in the legends match those in the panels (for example, some multi-compound curves in **Fig. 4c** and the supra-hex plots benefit from more explicit color labels in the legend).

Reply: We thank the reviewer for this comment and have thoroughly revised our Figures and Tables to remove these errors.

Comment #9. Metabolomics data are stated to be deposited in Metabolomics Workbench; it would be helpful to also explicitly state where WGS data for selected lines and the NSG recrudescent lines are deposited (e.g., SRA/BioProject accession), or to commit to deposition.

Reply: This has now been done. We have submitted the whole-genome sequencing data for the resistant clones obtained from in vitro selection pressure using ZY19489 as well as recrudescent parasites from the NSG mice experiments. The BioProject ID is PRJNA1376518 and the files are accessible via the link https://www.ncbi.nlm.nih.gov/bioproject/1376518. This information has now been added to the **Data availability** section of the manuscript which now reads as follows:
Data availability (page 35, lines 914 - 917):

From "~~Metabolomic data are publicly accessible through the NCBI Metabolomics Workbench (Project ID: PR002514). Other datasets analyzed during the current study are provided with this paper as Supplementary Material.~~"
To "Metabolomic data are publicly accessible through the NCBI Metabolomics Workbench (Project ID: PR002514). Raw reads of WGS data used in this study have been deposited in NCBI Sequenced Read Archive (SRA) under BioProject accession number PRJNA1376518 and are accessible *via* the link https://www.ncbi.nlm.nih.gov/bioproject/1376518.

Other datasets analyzed during the current study are provided with this paper as **Supplementary Material**.

Comment #10. A careful copy-editing pass would help with small inconsistencies (e.g., spacing around units, occasional missing hyphens such as "drug-selected", and minor grammatical issues). These do not affect scientific content but will improve readability.

Reply: We thank the Reviewer for this comment. We have now carefully gone through the manuscript and corrected some of the typographical and grammatic errors in the original draft. We are convinced that the current version is much-improved, more coherent and reads better.

Comment #11. In the Materials and Methods section, the notation for concentration units is inconsistent (e.g., use of both "w/w" and "wt/wt", and both "v/v" and "vol/vol"). Please standardize to a single format throughout (e.g., "% w/w" and "% v/v") in accordance with the journal's style.

Reply: *Nature Communications* uses the "% v/v and % w/w" style of concentration and volume abbreviation. We have therefore made this correction in the three parts of the manuscript where this was not standard with the rest. This is now written as follows:

Material and Methods (page 35, line 896):
From "~~...hour intervals with 5% (wt/vol) sorbitol…~~"
To "…hour intervals with 5% (w/v) sorbitol…"

Material and Methods (page 35, line 899):
From "...~~trophozoites were isolated with 0.05% (wt/vol) saponin and washed …~~"
To "…trophozoites were isolated with 0.05% (w/v) saponin and washed …"

Material and Methods (page 35, line 903):
From "~~...treated with 4% (wt/vol) SDS and 2.5% (vol/vol) pyridine~~"
To "…treated with 4% (w/v) SDS and 2.5% (v/v) pyridine"

Comment #12. Mitochondrial bc1 complex should be written as "mitochondrial bc1 complex".

Reply: The reference to "mitochondrial bc1 complex" only appears once in our manuscript. However, it is unclear from the reviewer's comment what revision is requested. Nonetheless, we realize that the "mitochondrial bc1 complex" is more accurately referred to and annotated as "mitochondrial cytochrome bc_1 complex". We can only hope that this was the suggestion the Reviewer was making and have subsequently made this change to reflect the more correct annotation. The new sentence now reads as follows:

Discussion (page 18, lines 453 - 455):
From "~~This concept has been demonstrated for other antimalarials, including PfDHODH, Pf20S proteasome and mitochondrial cytochrome bc_1 complex inhibitors[11], and is one of the rationales behind current triple ACT therapies~~"
To "Nevertheless, this hypothesis has been validated for other antimalarials, including PfDHODH, Pf20S proteasome and mitochondrial cytochrome bc_1 complex inhibitors[10], and is one of the rationales behind current triple ACT therapies."

Comment #13. Line 410, “in vivo” should be written in italic.

Reply: This has now been corrected. We have also ensured that this phrase is italicized the other five times it appears in the manuscript. The new line now reflects this correction as follows:

Discussion (page 17, line 413): “…compound also did not select for *in vivo* resistance in combination studies…”

Comment #14. At 2.5× IC_{50}, ZY19489 treatment is shown to cause a significant decrease in hemoglobin (Hb) levels (Fig 4i). Could the authors clarify why Hb levels are reduced specifically at 2.5× IC_{50} (e.g., stage-specific effects, timing of sampling, or assay sensitivity), and reconcile this with the broader model of DV Hb handling under ZY19489 exposure?

Reply: The reviewer raises and important question. We argue that the effect of ZY19489 on the levels of parasite hemoglobin heme is best observed incrementally between 1.0× to 2.0× IC_{50} concentrations (Fig. 4i), suggesting some activity of this compound during earlier steps of hemoglobin catabolism. However, we think it is likely that the significant drop in hemoglobin level observed 2.5× IC_{50} reflects injurious drug concentrations that inhibit the parasite’s ability to import sufficient hemoglobin levels for breakdown inside the digestive vacuole. It is important to note, as the reviewer has alluded, that ZY19489 is highly potent against the ring stage of the parasite which is the same parasite stage used to set up these fractionation experiments. This model fits with our proposed effect of hemoglobin catabolism, albeit in a concentration-sensitive and stage-specific manner. We have added this clarification in the **Results** section which now reads as follows.

Results (page 16, lines 397 - 400): “Nonetheless, treatment with ZY19489 caused significant increases in Hb levels at 1.5× and 2.0× IC_{50} concentrations but not at the more inhibitory concentration of 2.5× IC_{50} (Fig. 4i), suggesting some activity of this compound during earlier steps of Hb catabolism in a concentration- and stage-specific manner.”

Comment #15. Can the authors be consistent in writing E64D, as in the figures, and not E64d in the text?

Reply: This has now been done. We have written this as E64d all through the manuscript and in the Supplementary Figure and its Legend.

Supplementary Figure 11:
From “~~As a comparator, we tested E64D~~
To “As a comparator, we tested E64d”

Comment #16. The metabolomics data show alterations in pyrimidine intermediates alongside DV-related changes. Since ZY19489 is proposed primarily to affect DV hemoglobin handling, it would be helpful if the authors could clarify whether the pyrimidine perturbations are interpreted as a secondary consequence of DV dysfunction (e.g., impaired amino acid supply/energy status) rather than a direct effect on pyrimidine biosynthesis, and briefly outline the mechanistic link they envisage between DV damage and pyrimidine metabolism.

Reply: Our studies with the Llinas lab on a variety of different antimalarials have previously not found this signature of impaired Hb-derived peptide levels and perturbation of pyrimidine metabolism that was observed herein with ZY19489. We feel it is too preliminary to speculate on how these two signals could be related. Nonetheless, we note that ZY19489 and pyrimethamine (an inhibitor of pyrimidine biosynthesis) share a triaminopyrimidine substructure. We now comment on this in our revised Discussion as follows:

Discussion (page 17 lines 415 - 420): “An effect on pyrimidine biosynthesis might relate to the triaminopyrimidine substructure present in ZY19489 that is also observed in the antifolate inhibitor pyrimethamine (Supplementary Fig. 4), which earlier showed partial inhibition of pyrimidine biosynthesis in addition to its dominant effect on antifolate levels[36]. However, we do not know

whether the evidence of an effect of ZY19489 on Hb catabolism and levels of pyrimidine precursors suggests direct inhibition of both pathways or is indirectly related.”

RESPONSE TO REVIEWER COMMENTS

Reviewer #1: Reviewer #1 (Remarks to the Author):
The authors have addressed my previous queries in a professional and objective manner, leading to improvements in clarity, content, and overall scope of the manuscript. However, an oversimplified—and in parts potentially incorrect—statement has been introduced in the revised version in response to my Comment 3 concerning ligand occupation of the substrate-binding cavity of PfCRT. To address Comment 3, the authors added the following passage to the Discussion:
"In our simulations, ZY19489 consistently samples the same T76-adjacent region of the cavity, indicating that this molecule, CQ and PPQ engage largely overlapping binding volumes rather than distinct or spatially separated pockets. This interpretation aligns with our proteoliposome data where transport of CQ and PPQ by PfCRT variants that mediate resistance to these drugs was potently inhibited in the presence of ZY19489. This pattern suggests mutual exclusion which, in the context of PfCRT, does not imply steric hindrance but rather the intrinsic single-substrate nature of the transporter in which only one ligand can occupy the central cavity at a time and preclude binding or transport of another. The competition pattern observed experimentally is therefore consistent with all three compounds accessing the same substrate-binding cavity."

This paragraph raises several significant concerns:
1. Overgeneralization across PfCRT variants. Conclusions drawn primarily from simulations and uptake studies performed with PfCRTGB4 are extended broadly to other geographically distinct PfCRT variants without sufficient justification.

2. Failure to acknowledge structural and functional differences between PfCRT variants. The paragraph does not account for evidence indicating that PfCRTGB4 and PfCRTDd2 possess distinct substrate-binding cavity architectures. While PfCRTGB4 appears to accommodate only a single ligand at a time (either CQ or PPQ), as suggested by the authors' molecular dynamics simulations, PfCRTDd2 has been shown to bind both substrates simultaneously under a partial noncompetitive inhibition model. For PfCRTDd2, this interpretation is supported by extensive transport kinetics and complementary computational studies, which collectively suggest the presence of at least two distinct interaction sites within the binding cavity (PMID: 37285379; PMID: 25378409; PMID: 40140375; https://hdl.handle.net/1885/733780662).

3. Overinterpretation of transport data. The CQ and PPQ uptake data presented in Figures 2D and 2E are insufficient to support the conclusion that CQ, PPQ, and potentially ZY19489 act as mutually competitive inhibitors on PfCRTGB4. Demonstrating true competitive inhibition—and, by extension, a single binding site—would require more detailed transport kinetic analyses, such as those described in the studies cited above.

4. Conflation of findings from different PfCRT variants. The manuscript uses PfCRTDd2 for resistance selection experiments and PfCRTGB4 for molecular dynamics simulations and uptake assays yet treats

these variants as functionally equivalent. This is problematic, given their known differences in polymorphisms, transport kinetics, and inferred binding-site organization.

The intent of these comments is not to create undue difficulty for the authors, but rather to help prevent overstatements or inaccuracies that may undermine the manuscript at a later stage. I therefore recommend that the authors rework the cited paragraph in the Discussion to adopt a more nuanced interpretation, explicitly acknowledge the limitations arising from the use of PfCRT variants with distinct polymorphisms and transport properties, and avoid generalizing beyond what the data directly support. I am not requesting additional experimental work.

Reply: We thank the reviewer for these insightful comments. As a result, we have now amended the manuscript as follows:

1. In the **Results** section, we have made the following changes.

(page 8; line 179): from "Additionally, ZY19489 ~~disrupts~~ the residue interaction network around 76T…" to "Additionally, ZY19489 appeared to disrupt the residue interaction network around 76T…".

(page 8; lines 180 - 181): from "Interestingly, the 7G8+N246H protein ~~forms~~ a salt bridge between K80 and E207…" to "Interestingly, the 7G8+N246H protein was predicted to form a salt bridge between K80 and E207…"

(page 8; lines 182 – 184): from "This salt bridge formation suggests that the mutant protein might transiently adopt an occluded (partially closed-to-DV) conformation ~~and that the N246H mutation might confer resistance to ZY19489 by favoring this occluded state~~" to "This potential salt bridge formation suggests that the mutant protein might transiently adopt an occluded (partially closed-to-DV) conformation, which might contribute to ZY19489 resistance."

2. To further explain our interpretation of the data, our revised **Discussion** now states (page 17; lines 423 - 426): "Using metabolomics and Hb degradation assays, we also observed ZY19489-mediated perturbations of Hb catabolism, without a direct effect on downstream inhibition of heme detoxification (the primary MoA of CQ, PPQ and amodiaquine). These data suggest that a major MoA of ZY194899 is related to inhibition of Hb catabolism, which we propose is indicative of a primary action outside of the DV."

3. In the **Discussion** section, we have revised the paragraph cited above and included the references kindly provided by the reviewer. This paragraph now reads as follows:
(starting page 18; lines 449 - 470): from "In our simulations, ZY19489 consistently samples the same T76-adjacent region of the cavity, indicating that this molecule, CQ and PPQ engage largely overlapping binding volumes rather than distinct or spatially separated pockets. This interpretation aligns with our proteoliposome data where transport of CQ and PPQ by PfCRT variants that mediate resistance to these drugs was potently inhibited in the presence of ZY19489. This pattern suggests mutual exclusion which, in the context of PfCRT, does not imply steric hindrance but rather the intrinsic single-substrate nature of the transporter in which only one ligand can occupy the central cavity at a time and preclude binding or transport of another. The competition pattern observed experimentally is therefore consistent with all three compounds accessing the same substrate-binding cavity" to "In our simulations with the solved 7G8 isoform structure and its 7G8+N246H variant, ZY19489 consistently sampled the same T76-adjacent region of the cavity, suggesting that this molecule, CQ, and PPQ might partially overlap in their binding to these two PfCRT isoforms. This interpretation aligns with our proteoliposome data showing that ZY19489 inhibited transport of CQ and PPQ by their respective drug-resistant 7G8 and 7G8+F145I PfCRT variants. One explanation could be mutual exclusion which, in the context of these PfCRT isoforms, may suggest a single-substrate feature in which only one ligand could occupy

the central cavity at a time and preclude binding or transport of another. Our proteoliposome competition data suggest that all three compounds have at least partially overlapping cavity-binding properties. However, demonstrating true competitive inhibition and, by extension, confirmation of a single binding-site model would require more detailed transport kinetic analyses[40,43,44]. It is important to recall that our simulations and transport studies were conducted on 7G8 isoforms and as such this specific interpretation cannot be extended as a generalization to other geographically distinct PfCRT variants. In fact, variations in CQ and PPQ transport phenotypes have been observed between PfCRT isoforms expressing mutant alleles from different geographic regions[41,42], suggesting disparate interactions of these drugs with residues within the central cavity. Additionally, various PfCRT isoforms appear to possess distinct substrate-binding cavity architectures[43,44]. Whereas evidence to data suggest that 7G8 PfCRT may accommodate only a single ligand (either CQ or PPQ) at a time, transport kinetics and complementary computational studies have shown Dd2 PfCRT to bind both substrates simultaneously under a partial noncompetitive inhibition model[40,43,44], suggesting the presence of at least two distinct interaction sites within the cavity of this isoform. We also note that isogenic parasites expressing Dd2, GB4 and FCB *pfcrt* alleles are known to differ in their CQ resistance profiles[13], which also suggest differences in how these variants interact with antimalarial drugs."
